# Dysregulated Expression of Inflammasome and Extracellular Matrix Genes in *C9orf72*-ALS/FTD Microglia

**DOI:** 10.1080/17590914.2025.2542998

**Published:** 2025-08-07

**Authors:** Louise Thiry, Nisha S. Pulimood, Ye Man Tang, Stefano Stifani

**Affiliations:** Department of Neurology and Neurosurgery, Montreal Neurological Institute-Hospital, McGill University, 3801, rue University, Montreal, QC, Canada

**Keywords:** Amyotrophic lateral sclerosis, C9orf72, extracellular matrix, inflammasome, microglia, RNA sequencing

## Abstract

Hexanucleotide repeat expansion (HRE) in the non-coding region of the gene *C9orf72* is the most prevalent mutation in amyotrophic lateral sclerosis (ALS) and frontotemporal dementia (FTD). The *C9orf72* HRE contributes to neuron degeneration in ALS/FTD through both cell-autonomous mechanisms and non-cell autonomous disease processes involving glial cells such as microglia. The molecular mechanisms underlying the contribution of *C9orf72*-HRE microglia to neuron death in ALS/FTD remain to be fully elucidated. In this study, we generated microglia from human *C9orf72*-HRE and isogenic iPSCs using three different microglia derivation methods. RNA sequencing analysis reveals a cell-autonomous dysregulation of extracellular matrix (ECM) genes and genes involved in pathways underlying inflammasome activation in *C9orf72*-HRE microglia. In agreement with elevated expression of inflammasome components, conditioned media from *C9orf72*-HRE microglia enhance the death of *C9orf72*-HRE motor neurons implicating microglia-secreted molecules in non-cell autonomous mechanisms of *C9orf72* HRE pathology. These findings suggest that aberrant activation of inflammasome-mediated mechanisms in *C9orf72*-HRE microglia results in a pro-inflammatory phenotype that contributes to non-cell autonomous mechanisms of motor neuron degeneration in ALS/FTD.

## Introduction

Amyotrophic lateral sclerosis (ALS) is an incurable motor neuron disease characterized by neuronal death in the cerebral cortex, brain stem, and spinal cord. ALS usually starts during adulthood and causes progressive paralysis leading to the death of most sufferers within 2 to 5 years following diagnosis, mainly due to respiratory failure. ALS also affects several non-motor systems and subcortical structures, and cognitive and behavioral impairments similar to frontotemporal dementia (FTD) symptoms are present in up to 50% of ALS patients (Benatar et al., 2024; Kirola et al., 2022).

The majority of ALS cases are sporadic, implying that the mechanisms triggering motor neuron loss are not exclusively inherited. However, approximately 10%–15% of ALS cases are transmitted in families: these cases are referred to as familial ALS. Multiple deleterious variants in numerous genes either drive motor neuron degeneration, increase susceptibility to the disease, or influence the rate of disease progression. Several ALS-mutated genes have a negative impact on shared cellular processes, including, but not limited to, RNA metabolism and protein homeostasis, nucleocytoplasmic trafficking, endoplasmic reticulum stress, dynamics of ribonucleoprotein bodies, mitochondrial functions, and autophagy (Goutman et al., 2022; Nijs & Van Damme, 2024).

Intronic hexanucleotide (GGGGCC – ‘G_4_C_2_’) repeat expansions (HRE) in the gene *chromosome 9 open reading frame 72* (*C9orf72*) are the most frequent genetic cause of familial ALS/FTD (referred to as C9orf72-ALS hereafter) (DeJesus-Hernandez et al., 2011; Renton et al., 2011). In contrast to healthy individuals, who have, on average, 2-30 hexanucleotide repeats, C9orf72-ALS patients have *C9orf72* expansions with hundreds and even thousands of repeats. There are three (not mutually exclusive) proposed mechanisms to explain the effects of the *C9orf72* HRE: partial loss (haploinsufficiency) of *C9orf72* function caused by decreased expression of the C9orf72 mRNA and protein; toxic gain of function from the accumulation of sense G_4_C_2_ and antisense G_2_C_4_ repeat-containing RNA from bidirectionally transcribed expansions; and toxic gain of function from dipeptide repeat proteins aberrantly generated by repeat-associated non-ATG translation (Balendra & Isaacs, 2018; Gendron & Petrucelli, 2018). The combination of loss- and gain-of-function mechanisms associated with the *C9orf72* HRE have wide-ranging consequences in multiple cell types, in agreement with the broad expression pattern of the *C9orf72* gene and the localization of the C9orf72 protein to multiple cellular compartments. Multiple cellular processes are affected in C9orf72-ALS motor neurons leading to altered mitochondria, oxidative stress, impaired autophagy, and impaired RNA metabolism, among other phenotypes (Chew et al., 2015; de Calbiac et al., 2024; Donnelly et al., 2013; Lopez-Gonzalez et al., 2016; Mehta et al., 2021; Tran et al., 2015).

Like other neurodegenerative diseases, ALS is characterized by extensive neuroinflammation involving astrogliosis, activation of microglia, and infiltration of peripheral immune cells at sites of neuronal degeneration. Glial cells have a significant impact on disease onset and progression by mediating non-cell autonomous disease mechanisms (Appel et al., 2021; Calma et al., 2024; Izrael et al., 2020; Majewski et al., 2025). Intercellular communication between motor neurons and microglia, the resident immune cells of the central nervous system, play an important role in the degeneration of motor circuits in ALS. Microglia have the potential to perform both neuroprotective and neurotoxic roles in ALS, depending on their activation states in response to the local microenvironment. During initial phases of the disease microglia can protect motor neurons by performing scavenging, anti-inflammatory, and neuroprotective functions. During disease progression, however, with rising oxidative stress and injury within motor neurons, microglia can contribute to motor-neuron death by switching to pro-inflammatory and neurotoxic phenotypes (Beers & Appel, 2019; Majewski et al., 2025; Mimic et al., 2023). Much remains to be learned about the mechanisms underlying the dynamic intercellular communication between motor neurons and microglia in ALS.

*C9orf72* is robustly expressed in microglia and other myeloid-lineage cells. Analysis of post-mortem brains from C9orf72-ALS patients revealed morphological and immunological signs suggestive of microglia activation (Lant et al., 2014), as did *ex vivo* diffusion tensor MRI studies of tissues from ALS patients including *C9orf72* cases (Cardenas et al., 2017). Evidence that *C9orf72* is required for the normal function of myeloid cells first came from studies in mice lacking the murine *C9orf72* ortholog in all tissues. *C9orf72*^-/-^ (homozygous knockout) mice develop and age normally but exhibit several immune cell phenotypes, including lysosomal accumulation and altered immune responses in macrophages and microglia, with associated age-related neuroinflammation (Atanasio et al., 2016; Burberry et al., 2016; O’Rourke et al., 2016; Sudria-Lopez et al., 2016). Studies in which the human *C9orf72* HRE was introduced into mice showed signs of pathological expression of *C9orf72* HRE in the cortex including the appearance of hallmarks of microglia activation (Nicholson et al., 2018; Zhang et al., 2014). Transcriptomic analysis of *C9orf72* knockout mouse microglia revealed gene expression changes consistent with an inflammatory phenotype and correlated with increased microglia-mediated synaptic loss, which together imply that loss of *C9orf72* results in a proinflammatory and neurotoxic microglia phenotype (Lall et al., 2021). More recent studies using microglia generated from induced pluripotent stem cells (iPSCs) derived from C9orf72-ALS patients have shown that human C9orf72-ALS microglia exhibit typical hallmarks of *C9orf72* HRE pathology such as G_4_C_2_-repeat RNA foci, expression of dipeptide repeat proteins, and decreased expression level of C9orf72 (Lorenzini et al., 2023; Vahsen et al., 2023). Moreover, lipopolysaccharide (LPS)-primed C9orf72-ALS microglia were observed to have a pro-inflammatory phenotype and to be toxic to motor neurons when compared to healthy and/or isogenic microglia as controls (Banerjee et al., 2023; Vahsen et al., 2023).

Recent studies in both animal and human cell models have shown that the *C9orf72* HRE is associated with activation of the Nucleotide-Binding-Oligomerization Domain (NOD)-and Leucine-Rich Repeat (LRR)-containing (NLR) family, Pyrin-Domain-Containing 3 (NLRP3) inflammasome in microglia (Banerjee et al., 2023; Fu et al., 2023; Rivers-Auty et al., 2024; Shu et al., 2023; Trageser et al., 2023). The NLRP3 inflammasome is a cellular immune sensor that mediates pathological inflammation in autoimmune, metabolic, malignant, and neurodegenerative conditions (Barnett et al., 2023; Fu & Wu, 2023; Sharma & Kanneganti, 2021). It detects stress signals, such as tissue damage or pathogen invasion, via immune cell components referred to as pattern recognition receptors (PRRs). For instance, the PRR Toll-like Receptor 4 (TLR4) can detect ‘danger signals’ and respond by enlisting Nuclear Factor-kappaB (NF-κB) to activate the transcription of genes encoding proteins involved in the NLRP3 inflammasome, including *NLRP3* itself. The NLRP3 protein assembles with other components, such as Apoptosis-associated Speck-like protein containing a Caspase recruitment domain (ASC), to form the active inflammasome complex. The NLRP3 complex activates Caspase-1 (CASP1) which in turn induces the release of inflammatory cytokines like Interleukin-1 beta (IL1B) and IL18 and the activation of pyroptosis, a highly inflammatory form of lytic cell death (Barnett et al., 2023; Fu & Wu, 2023; Sharma & Kanneganti, 2021). Little is known, however, about the molecular mechanisms underlying NLRP3 inflammasome activation in C9orf72-ALS microglia.

In this study, we characterized the transcriptomic and secretome changes associated with *C9orf72* amplification in microglia derived from C9orf72-ALS iPSCs, and isogenic control iPSCs, using three different microglia differentiation methods. We specifically compared derivation protocols that generate microglia with distinct intrinsic inflammatory profiles to alleviate the possibility that differentiation protocol-specific basal activation of the induced microglia might confound the study of the impact of the *C9orf72* HRE on inflammatory mechanisms. Here we show a consistent dysregulation of several genes involved in pathways underlying NLRP3 inflammasome activation in C9orf72-ALS microglia. Numerous dysregulated genes encode extracellular matrix (ECM) proteins, suggesting a link between ECM remodelling and inflammasome activation in C9orf72-ALS microglia. We show further that conditioned media from C9orf72-ALS microglia enhance the death of C9orf72-ALS motor neurons *in vitro*. We hypothesize that cell-autonomous alterations in gene expression associated with inflammasome activation in microglia contribute to neuronal death in C9orf72-ALS. The transcriptomic data generated by this study would serve as an objective source of hypothesis-generating data that may reveal novel mechanisms of microglial involvement in C9orf72-ALS.

## Results

### Generation of C9orf72-ALS iPSC-Derived Microglia Using Three Different Differentiation Protocols

Activation of the NLRP3 inflammasome in microglia was observed in both mouse and human cells harbouring the *C9orf72* HRE (Banerjee et al., 2023; Fu et al., 2023; Rivers-Auty et al., 2024; Shu et al., 2023; Trageser et al., 2023). Little information is available, however, about the gene networks that are dysregulated as part of the NLRP3 inflammasome activation in C9orf72-ALS microglia. To address this gap in knowledge, we implemented a comprehensive RNA sequencing (RNAseq) study of human microglia derived from C9orf72-ALS iPSC lines, and matching isogenic iPSCs. Four previously studied (Chi et al., 2023; Gao et al., 2024; Ho et al., 2021; Thiry et al., 2024) iPSC lines were used for microglia derivation: the C9orf72-ALS iPSC lines CS52iALS-C9n6 (CS52-C9n6-M hereafter) and CS29iALS-C9n1 (CS29-C9n1-M), and their matching isogenic (ISO) control lines CS52iALS-C9n6.ISOxx (CS52-C9n6-ISO) and CS29iALS-C9n1.ISOxx (CS29-C9n1-ISO). Previous studies have demonstrated the presence of *C9orf72* HRE RNA foci and dipeptide repeat expression in neuromuscular organoids generated using these C9orf72-ALS iPSC lines (Gao et al., 2024), providing evidence that they can recapitulate *C9orf72* HRE-associated pathological features *in vitro*.

**Figure 1.**
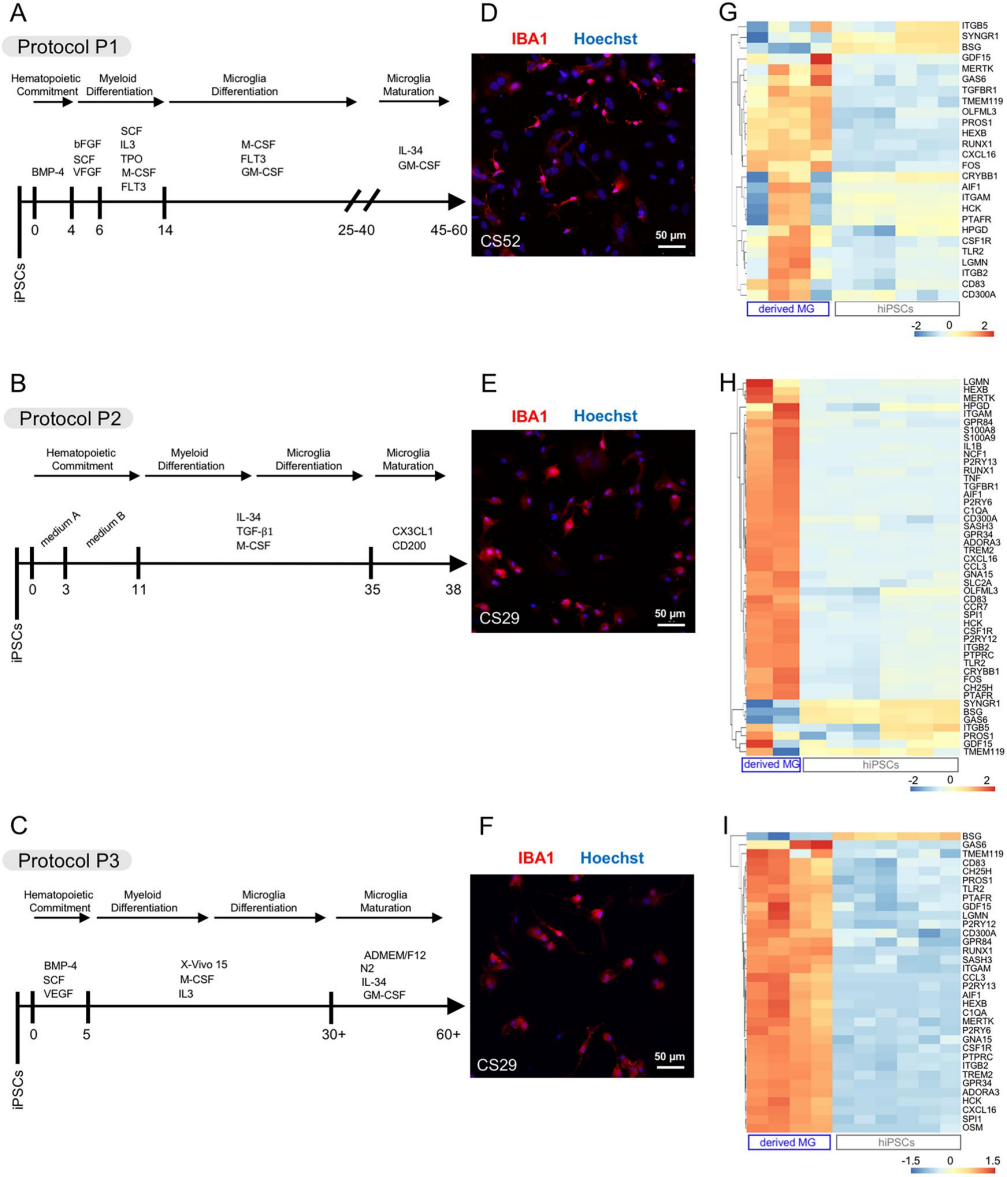
Generation and characterization of iPSC-derived microglia using three separate methods. (A–C) Schematics of three microglia differentiation protocols, referred to as “protocol P1” adapted from Douvaras et al. (A), “protocol P2” adapted from McQuade et al. (B), and “protocol P3” adapted from Haenseler et al. (C). (D–F) Representative images of microglia preparations differentiated from iPSC lines CS52-C9n6-ISO (CS52) and CS29-C9n1-ISO (CS29) with protocol P1 (D), P2 (E), or P3 (F) and immunostained with anti-IBA1 antibody (red) and counterstained with Hoechst (blue). Scale bar, 50 µm. (G–I) Heatmaps showing known microglia/macrophage marker gene expression in undifferentiated iPSCs (obtained from publicly available datasets) or microglia preparations differentiated from the CS52 and CS29 iPSC lines with protocol P1 (G), P2 (H) or P3 (I). Each column of the heatmap corresponds to one sample (undifferentiated iPSCs (hiPSCs) or isogenic iPSC-derived microglia (derived MG), as indicated at the bottom, and each line corresponds to one gene. Subgroups of genes with correlated expression patterns are portrayed by the dendrogram on the left side of each heatmap. Relative levels of gene expression are indicated by a color scale, with cool colors corresponding to low expression and warm colors corresponding to increased/high expression. Hierarchical clustering of rows was applied to the heatmaps to group genes with correlated expression patterns, resulting in different gene lists, depending on the samples analyzed in the given heatmap. The gene list used for this analysis was as follows: *HEXB, P2RY12, CXC3CR1, P2RY13, TREM2, S100A8, TMEM119, S100A9, RNASE4, GPR34, FCRLS, SIGLECH, OLFML3, FOS, SCLO2B1, TGFBR1, SLC2A5, CAMP, ITGB5, CRYBB1, SYNGR1, GPR56, NGP, CMRF35, HPGD, GPR34, MERTK, C1QA, PROS1, GAS6, ITGAM, ITGB2, CSF1R, PTPRC, AIF1, ADORA3, LGMN, GPR84, CCR7, BCL2A1D, TNF, NCF1, GDF15, OSM, LMC25, H2-OA, CD83, CCL3, GNA15, IL1B, PFAU, CCL9, TMEM119, C1QA, LRF8, CXCL16, CH25H, HCK, CCL12, PTAFR, CD300A, LRT5, STPI1, SELPIG, SASH3, BSG, TLR2, P2RY6, CDI4, BCL2A1A, BCL2A1C, RUNX1, and SPI1.*

We generated iPSC-derived microglia using three separate differentiation methods based on previously published protocols (Douvaras et al., 2017; Haenseler et al., 2017; McQuade et al., 2018). The use of multiple derivation protocols was aimed at mitigating the confounding effects that might result from the variability in the basal properties of iPSC-derived microglia generated using different *in vitro* differentiation methods (Tang et al., 2022). Hereafter, we shall operationally refer to the three microglia differentiation protocols used in the present study as protocols P1 (Douvaras et al., 2017), P2 (McQuade et al., 2018), and P3 (Haenseler et al., 2017) (Figure 1A–C). With all protocols, we allowed microglia to acquire a mature phenotype *in vitro* by differentiating the cells for at least as long as indicated in the original publications and then confirming robustness and maturity of the induced cultures as we have previously described (Tang et al., 2022).

Examination of isogenic iPSC lines CS52-C9n6-ISO and CS29-C9n1-ISO by immunocytochemistry showed that all three microglia derivation protocols generated cultures highly enriched in cells expressing typical microglia/macrophage markers such as IBA1 (Figure 1D–F) and P2RY12 (Supplemental Figure S1). Moreover, RNAseq data showed that the majority of 49 previously characterized (Butovsky et al., 2014; Hickman et al., 2013; Muffat et al., 2016; Zhang et al., 2014) microglia/macrophage cell marker genes were upregulated in microglia generated with all three protocols, when compared to RNAseq data from separate undifferentiated iPSC lines obtained using publicly available datasets (Figure 1G–I).

**Figure 2.**
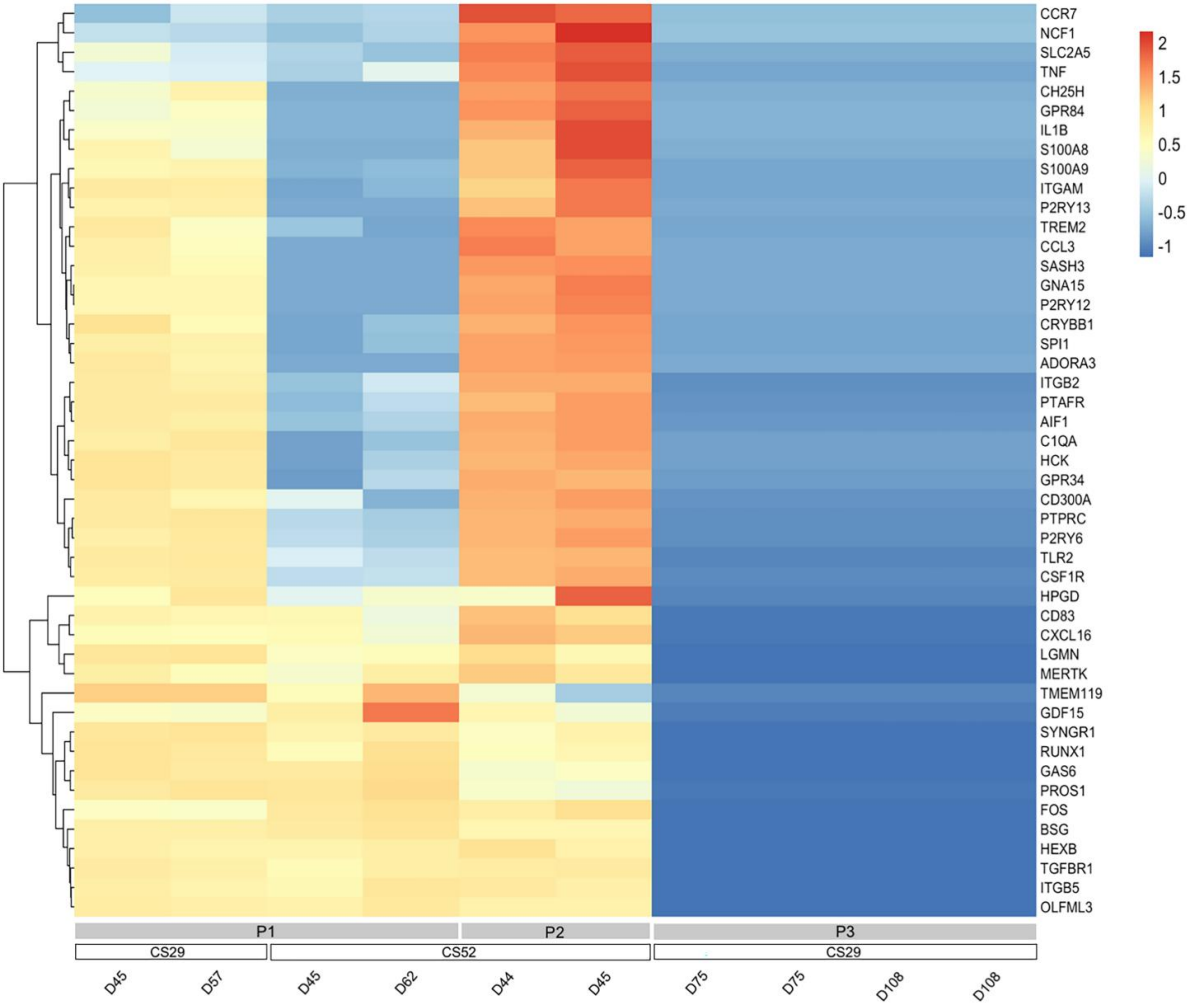
Comparison of microglia/macrophage gene expression in iPSC-derived microglia obtained with three different protocols. Heatmap showing expression of known myeloid/macrophage/microglial cell marker genes in microglia preparations differentiated from the CS52-C9n6-ISO (CS52) and CS29-C9n1-ISO (CS29) iPSC lines with protocols P1, P2, or P3. Each column corresponds to one isogenic sample (examined at the indicated differentiation day) and each line corresponds to one gene. Subgroups of genes with correlated expression patterns are portrayed by the dendrogram on the left side of the heatmap. Relative levels of gene expression are indicated by a color scale, with cool colors corresponding to low expression and warm colors corresponding to increased/high expression. The gene list used for this analysis was the same as the list shown in the legend to Figure 1, with addition of the following macrophage marker genes: *EMILIN2*, *SELL*, *HP.*

Microglia generated using protocol P2 had the highest number of significantly upregulated genes encoding pro-inflammatory molecules, such as Chemokine (C-C motif) ligand 3 (CCL3), C-C Motif Chemokine Receptor 7 (CCR7), C-X-C Motif Chemokine Ligand 16 (CXCL16), G Protein Coupled Receptor 84 (GPR84), Hematopoietic Cell Kinase (HCK), IL1B, S100 Calcium Binding Protein A9 (S100A9), S100 Calcium Binding Protein B (S100B), Tumor Necrosis Factor (TNF), when compared to microglia generated with protocols P1 and P3 (Figure 1G–I). Consistently, direct comparison of the expression of microglia/macrophage genes across a panel of RNAseq datasets from microglia generated using the three different methods, and collected at varying time points, showed that protocol 2-microglia had the highest levels of pro-inflammatory genes. Protocol P3 had the lowest levels of the same genes, suggesting that method 3-microglia are the least developmentally mature and/or least activated compared to microglia obtained using the other two protocols, even after long *in vitro* differentiation times (Figure 2). We observed further that differentiation protocol P2 generated cells with the most robust expression of microglial markers such as *P2RY12*, *TMEM119*, and *SLC2A5*, while more specific macrophage markers like *EMILIN2*, *HAPTOGLOBIN* (*HP*), and *SELECTIN L* (*SELL*) (Haage et al., 2019) were similarly represented in cells obtained with protocols P1 and P2. Lastly, more generic myeloid markers such as *RUNX1*, *SPI1*, and *CD14* where higher in microglia obtained with protocols P1 and P2 compared to P3 (Figure 2). Together, these observations suggest that microglia generated using method P2 are more pro-inflammatory than microglia obtained using protocols P1 and P3 under comparable experimental conditions.

### Dysregulated Transcriptome of C9orf72-ALS iPSC-Derived Microglia

All iPSC lines, C9orf72-ALS and isogenic, were used to generate microglia using at least two of the three derivation methods described above. All microglia preparations (25 in total) were analysed by RNAseq. To search for cell-intrinsic transcriptomic changes associated with the *C9orf72* HRE, all microglia preparations were not exposed to lipopolysaccharide (LPS) or other treatments inducing microglia activation prior to RNA collection.

**Figure 3.**
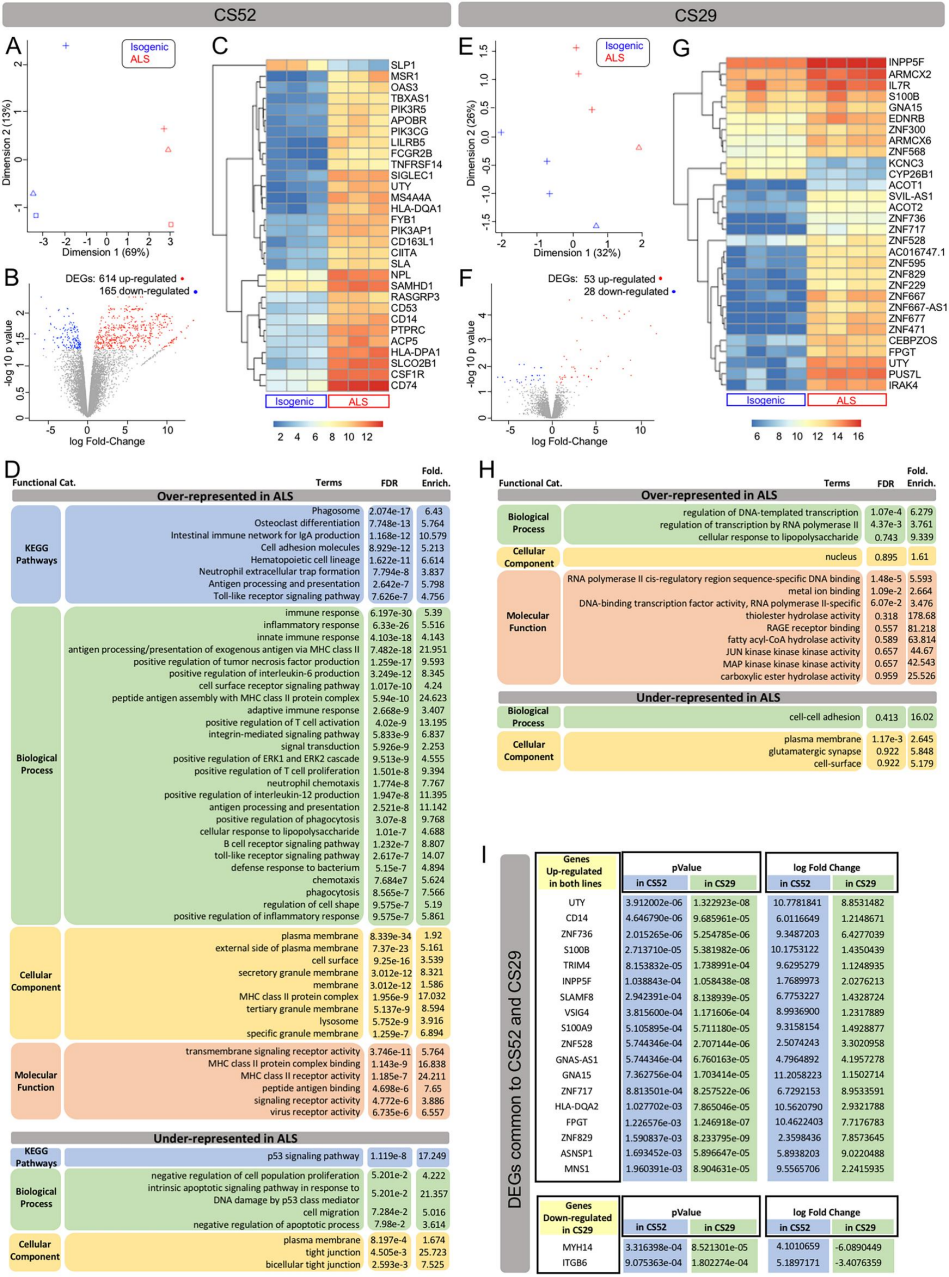
Differential gene expression between corrected isogenic and C9orf72-ALS microglia derived using protocol P1. (A–H**)** Differential gene expression and gene ontology analysis of CS52-C9n6-M versus CS52-C9n6-ISO microglia (CS52 collectively) (A–D) or CS29-C9n1-M versus CS29-C9n1-ISO microglia (CS29 collectively) (E–H). (A and E) Principal component analysis plots showing the relationship between the transcriptomic profiles of CS52 (A) or CS29 (E) iPSC-derived microglia. Each plotted data point represents one biological replicate, and the different symbols refer to the different batches. Blue data points correspond to corrected isogenic samples (Isogenic); red data points correspond to C9orf72-ALS samples (ALS). The percentages of variance explained for dimensions 1 and 2 are indicated along the axes. (B and F) Volcano plots showing the log Fold-Change (X‐axis) and -log10 p value (Y‐axis), highlighting the differentially expressed genes (DEGs) in C9orf72-ALS microglia compared to isogenic microglia differentiated from the CS52 (B) or CS29 (F) iPSC lines. Each plotted data point represents one gene: grey data points correspond to genes that are not DEGs, red data points correspond to up-regulated DEGs, and blue data points correspond to down-regulated DEGs (p‐adjusted <0.05, with no Fold Change cutoff). The numbers of up- and down-regulated DEGs are indicated on top of each volcano plot. (C and G) Heatmaps of the topmost DEGs in C9orf72-ALS microglia compared to isogenic microglia differentiated from the CS52 (C) or CS29 (G) iPSC lines. For each heatmap, each column corresponds to one sample (Isogenic or ALS, as indicated at the bottom) and each line corresponds to one gene. Relative levels of gene expression are indicated by a color scale, with cool colors corresponding to low expression and warm colors corresponding to increased/high expression. (D and H) Gene ontology analysis tables showing KEGG pathways, biological processes, cellular components and molecular functions that are significantly over- or under-represented in C9orf72-ALS compared to isogenic microglia differentiated from either the CS52 (D) or CS29 (H) iPSC line. (I) Table showing the 20 DEGs common to CS52-C9n6-M and CS29-C9n1-M microglia. The p value and log Fold Change are indicated for each gene.

We first analyzed RNA isolated from three separate microglia cultures derived from the C9orf72-ALS iPSC line CS52-C9n6-M and three microglia cultures from the matching isogenic control line CS52-C9n6-ISO. These 6 biological samples were obtained using differentiation protocol P1, which in our hands generates microglia that are less intrinsically pro-inflammatory than microglia generated using method 2 (Figure 2). Principle component analysis showed that CS52-C9n6-M microglia grouped together along one dimension, clustering away from CS52-C9n6-ISO microglia (Figure 3A). Comparing the transcriptomic data from these six separate preparations using an EdgeR (version 4.0) analysis pipeline for differentially expressed gene (DEG) analysis (Chen et al., 2016), we identified 614 genes that were significantly upregulated in C9orf72-ALS microglia, while 165 genes were downregulated (adjusted p-value (false discovery rate; FDR) <0.05, with no fold-change cutoff) (Figure 3B; entire DEG list available in Supplemental Information). The top 30 most upregulated DEGs in CS52-C9n6-M microglia included several genes that have previously been associated with ALS, such as *Cluster of Differentiation 14* (*CD14*), *Class II Major Histocompatibility Complex Transactivator* (*CIITA*), *Fc Gamma Receptor IIb* (*FCGR2B*), *Major Histocompatibility Complex, Class II, DQ Alpha 1* (*HLA-DQA1*), and *Colony Stimulating Factor 1 Receptor* (*CSF1R*) (Beers et al., 2022; Chi et al., 2023; Martínez-Muriana et al., 2016; Sanfilippo et al., 2017; Xie et al., 2021) (Figure 3C).

Several genes involved in microglia/macrophage activation were also present within the top 30 DEGs, including *Cluster of Differentiation 74* (*CD74*), *Macrophage Scavenger Receptor 1* (*MSR1*), *Phosphoinositide-3-Kinase Regulatory Subunit 5* (*PIK3R5*), *Phosphatidylinositol-4,5-Bisphosphate 3-Kinase Catalytic Subunit Gamma* (*PIK3CG*), to name a few (Gu et al., 2024; Ji et al., 2022; Lajqi et al., 2019; Peferoen et al., 2015). Consistently, combined Gene Ontology (GO) and Kyoto Encyclopedia of Genes and Genomes (KEGG) analysis of the upregulated DEGs in CS52-C9n6-M microglia generated using protocol P1 revealed enrichment in pathways associated with ‘immune response’, ‘inflammatory response’, ‘innate immune response’, to name a few (Figure 3D). The latter finding resembles the observation by Vahsen and colleagues that LPS-primed C9orf72-ALS iPSC-derived microglia (obtained using a protocol resembling protocol P3 in our study) exhibit enrichment in DEGs involved in pathways associated with immune-cell activation (Vahsen et al., 2023).

Importantly, a number of the top 30 DEGs depicted in Figure 3C (*CD14*, *CIITA*, *CSF1R*, *PIK3CG*, *Protein Tyrosine Phosphatase Receptor Type C* (*PTPRC;* also referred to as *Cluster of Differentiation 45* (*CD45*))*, Sialic Acid Binding Ig Like Lectin 1* (*SIGLEC1*; also known as *Cluster of Differentiation 169* (CD169)) encode proteins previously implicated in the modulation of pyroptosis mechanisms, including the NLRP3 inflammasome (Du et al., 2020; Lu et al., 2023; Radian et al., 2015; Souza de Lima et al., 2017; Vasudevan et al., 2022; Vidmar et al., 2019; Wang et al., 2022b). In line with this finding, we identified genes encoding core components of the NLRP3 inflammasome, including *NLRP3*, *CASP1*, and *IL1B*, among the significantly upregulated DEGs in C9orf72-ALS microglia, even though not among the top 30 (Figure S2). Importantly, other genes involved in inflammasome/pyroptosis mechanisms, such as *NLRP2*, *NLR Family CARD Domain Containing 4* (*NLRC4*), *MEFV innate immunity regulator, pyrin* (*MEFV*), and *ninjurin-1* (*NINJ1*) (Barnett et al., 2023), were also among the significantly upregulated DEGs (Figure S2). These observations are consistent with the previous finding that NLRP3 activation can occur in concert with other inflammasome pathways, including NLRC4-mediated processes (Freeman et al., 2017; Ip & Medzhitov, 2015).

When the same analysis was conducted using eight RNAseq datasets from microglia derived from the C9orf72-ALS iPSC line CS29-C9n1-M and its matching isogenic control CS29-C9n1-ISO, differentiated using protocol P1, we identified 53 significantly upregulated genes in C9orf72-ALS microglia, while 28 genes were downregulated (Figure 3E, F; entire DEG list available in Supplemental Information). Interestingly, isogenic microglia generated from CS29-C9n1-ISO iPSCs appeared intrinsically more inflammatory than microglia generated from CS52-C9n6-ISO (Figure 2), perhaps explaining in part the lower number of DEGs compared to the latter. The list of top 30 DEGs in CS29-C9n1-M microglia revealed predominantly gene-regulation- and proliferation-associated genes, including numerous zinc finger protein-encoding genes (Figure 3G). The observation of several genes encoding zinc finger-domain proteins among the top DEGs agrees with the identification of more than 20 zinc-finger family members as genes implicated in inflammation in ALS from blood transcriptome profiles (Pappalardo et al., 2024). GO and KEGG analysis of the upregulated DEGs in CS29-C9n1-M microglia revealed enrichment in processes associated with transcription, DNA binding, RNA polymerase II activity, but no significant enrichment in immune processes apart from the term ‘response to LPS’ (Figure 3H). However, when we searched for DEGs common to both CS52-C9n6-M and CS29-C9n1-M microglia derived using protocol P1, we identified 18 significantly upregulated DEGs in both cases, as well as two common DEGs that were upregulated in CS52-C9n6-M microglia but downregulated in CS29-C9n1-M microglia (Figure 3I). These 20 common DEGs include 6 genes previously implicated in NLRP3 inflammasome-mediated mechanisms: *CD14*, *Integrin Subunit Beta 6* (*ITGB6*), *S100A9*, *S100B*, *SLAM Family Member 8* (*SLAMF8*), and *V-Set and Immunoglobulin Domain Containing 4* (*VSIG4*) (Arterbery et al., 2018; Bi et al., 2019; Huang et al., 2019; Li et al., 2025; Pruenster et al., 2023; Vizuete et al., 2022; Wang et al., 2024). This finding further suggests that mechanisms involving the NLRP3 inflammasome are dysregulated in C9orf72-ALS microglia.

**Figure 4.**
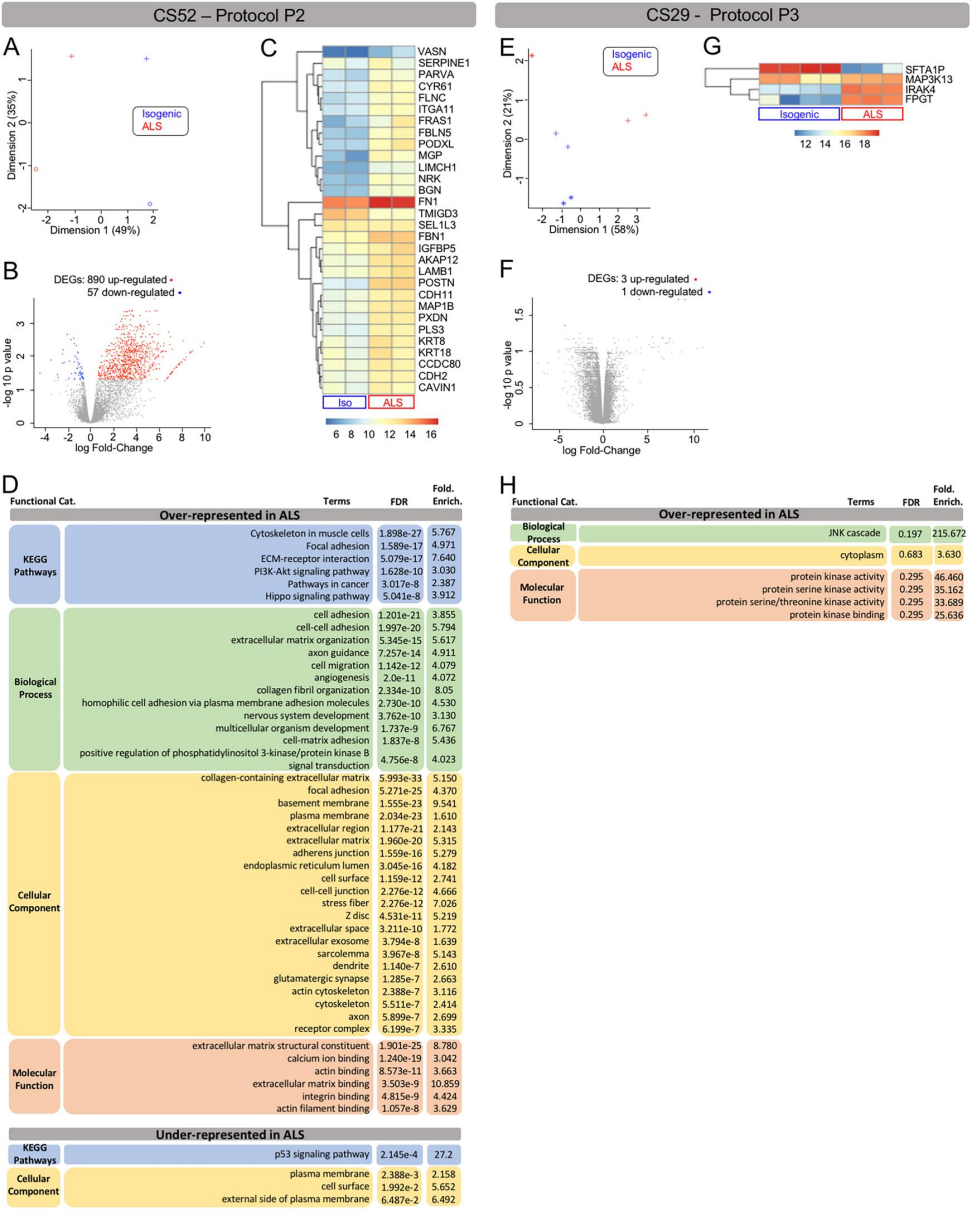
Differential gene expression between corrected isogenic and C9orf72-ALS microglia derived using protocols P1 and P2. (A–H) Differential gene expression and gene ontology analysis of CS52-C9n6-M versus CS52-C9n6-ISO microglia (CS52 collectively) generated using protocol P2 (A–D) or CS29-C9n1-M versus CS29-C9n1-ISO microglia (CS29 collectively) generated using protocol P3 (E–H). (A and E) Principal component analysis plots showing the relationship between the transcriptomic profiles of CS52 microglia generated with protocol P2 (A) or CS29 microglia generated with protocol P3 (E). Each plotted data point represents one biological replicate, and the different symbols refer to the different batches. Blue data points correspond to isogenic samples (Isogenic); red data points correspond to C9orf72-ALS samples (ALS). The percentages of variance explained for dimensions 1 and 2 are indicated along the axes. (B and F) Volcano plots showing the log Fold-Change (X‐axis) and -log10 p value (Y‐axis), highlighting the differentially expressed genes (DEGs) in C9orf72-ALS microglia compared to isogenic microglia differentiated from the CS52 (B) or CS29 (F) iPSC lines using protocols P2 or P3, respectively. Each plotted data point represents one gene: grey data points correspond to genes that are not DEG, red data points correspond to up-regulated DEGs, and blue data points correspond to down-regulated DEGs (p‐adjusted <0.05, logFC >0.26). The numbers of up- and down-regulated DEGs are indicated on top of each volcano plot. (C and G) Heatmaps of the topmost DEGs in C9orf72-ALS microglia compared to isogenic microglia differentiated from either the CS52 iPSC line with protocol P2 (C) or the CS29 iPSC line with protocol P3 (G). For each heatmap, each column corresponds to one sample (Isogenic or ALS, as indicated at the bottom) and each line corresponds to one gene. Relative levels of gene expression are indicated by a color scale, with cool colors corresponding to low expression and warm colors corresponding to increased/high expression. (D and H) Gene ontology analysis tables showing KEGG pathways, biological processes, cellular components and molecular functions that are significantly over- or under-represented in C9orf72-ALS compared to isogenic microglia differentiated from either the CS52 iPSC line with protocol P2 (D) or CS29 iPSC line with protocol P3 (H).

The commonality of 20 DEGs in microglia that exhibit varying degrees of mutation-associated alterations in gene expression suggests that at least some of these transcriptomic changes may represent physiologically relevant DEGs in C9orf72-ALS microglia. To test this possibility, and in the process search for additional DEGs, we next investigated microglia generated from CS52-C9n6-M iPSCs, and matching isogenic iPSCs, using differentiation protocol P2. As mentioned, this derivation method gives rise to microglia that appear more activated and inflammatory than those obtained with protocols 1 and 3 in our hands (Figure 2). We identified 890 genes significantly upregulated in CS52-C9n6-M microglia, with 57 genes significantly downregulated (Figure 4A, B; entire DEG list available in Supplemental Information). Perhaps not surprisingly given the phenotypic differences between microglia generated using protocols P1 and P2, the top 30 DEGs in CS52-C9n6-M microglia generated with differentiation method P2 were mainly different from the top 30 DEGs identified using differentiation method P1(Figure 4C). Importantly, however, genes associated with the NLRP3 inflammasome were also present among the top 30 DEGs identified in CS52-C9n6-M microglia generated with protocol P2. These included *Biglycan* (*BGN*), *Caveolae Associated Protein 1* (*CAVIN1*) (also termed *Polymerase 1 and Transcript Release Factor* (*PTRF*)), *Cystein Rich Angiogenic Inducer 61* (*CYR61*) (also known as *Cellular Communication Network Factor 1* (*CCN1*)), and *Periostin* (*POSTN)* (Babelova et al., 2009; Bai et al., 2010; Jin et al., 2024; Yao et al., 2020; Zheng et al., 2013; Zhou et al., 2021) (Figure 4C). Although not among the top 30 DEGs, *SIGLEC1* and *ITGB6* were also among the significantly upregulated genes in CS52-C9n6-M microglia generated with protocol P2, in agreement with protocol P1 RNAseq data. We observed that several inflammasome-associated top 30 DEGs identified using both protocols P1 and P2 encode ECM/matricellular proteins, such as *ITGB6*, *SIGLEC1*, *VSIG4*, *BGN*, *CYR61*, and *POSTN*. Upregulation of additional ECM genes was detected in C9orf72-ALS microglia generated with both differentiation protocols P1 and P2, including members of the non-fibrillar collagen-, laminin-, and integrin-protein families (Figure S3). In agreement with this, GO/KEGG analysis of molecular and cellular pathways associated with up-regulated DEGs detected using differentiation protocol 2 revealed mechanisms converging on cell adhesion, extracellular matrix organization, and cell-matrix adhesion (Figure 4D). These findings further suggest that genes involved in NLRP3 inflammasome/pyroptosis are significant DEGs in C9orf72-ALS ALS microglia and implicate changes at the ECM in these events.

To further examine cell-intrinsic gene expression changes in C9orf72-ALS microglia, we next generated microglia from CS29-C9n1-M and CS29-C9n1-ISO iPSCs using protocol P3. As mentioned above, in our hands this method generates microglia that appear less developmentally mature and less inflammatory than those obtained using the other two methods. Although only 4 DEGs were identified in CS29-C9n1-M microglia generated using protocol P3 (Figure 4E–H), one of these DEGs, *IL-1 Receptor–Associated Kinase 4* (*IRAK4*), encodes a protein previously shown to be involved in the NLRP3 inflammasome (Leaf et al., 2017). Of note, *IRAK4* was also identified as a top 30 DEG in CS29-C9n1-M microglia generated using protocol P1 (Figure 3G). One additional DEG, *Fucose-1-Phosphate Guanylyltransferase* (*FPGT*), was conserved across CS29-C9n1-M microglia generated using protocols P1 and P3, as well as CS52-C9n6-M microglia obtained using protocols P1 and P2 (Figures 3G and 4G; Supplemental Information). *FPGT* has previously been implicated in inflammation in ALS from blood transcriptome profiles (Pappalardo et al., 2024). A third DEG was also of interest in the context of glial cell activation and caspase-mediated mechanisms of cell death, namely *Mitogen-Activated Protein Kinase Kinase Kinase 13* (*MAP3K13*, also known as *Leucine Zipper-Bearing Kinase* (*LZK*). MAP3K13/LZK promotes astrocyte reactivation and can activate NF-κB signaling (Chen et al., 2018; Masaki et al., 2003).

We next searched for DEGs common to all 25 RNAseq datasets encompassing different iPSC lines and different differentiation protocols. This analysis identified 15 up-regulated DEGs common to all conditions tested (Figure S4). The top 6 shared DEGs by p values (*ASNSP1*, *FPGT*, *ZNF736*, *ZNF717*, *GNAS-AS1*, and *UTY*) were also among the 18 up-regulated DEGs common to both CS52-C9n6-M and CS29-C9n1-M microglia using differentiation protocols P1 and P2 (cf. Figure S4 and Figure 3I). Several of the 15 up-regulated DEGs common to all conditions tested are directly or indirectly implicated in immune mechanisms, including *CXCL5*, *FPGT*, *GNAS-AS1*, *ZNF717*, *ZNF736*, *ZNF880* (Dong et al., 2023; Hsiao et al., 2021; Lin et al., 2025; Liu et al., 2024a; Pappalardo et al., 2024; Wang et al., 2016). Two additional DEGs associated with the *C9orf72* HRE in all 25 RNAseq datasets are of significance in the context of inflammation and ALS. *AC004846.1* encodes a long non-coding RNA (lncRNA) identified as a pyroptosis-related lncRNA in colorectal cancer (Cai et al., 2022). *Protocadherin Alpha 2* (*PCDHA2*), encoding a transmembrane protein involved in cell-cell adhesion, is a member of the *Protocadherin Alpha* gene cluster composed of 15 cadherin superfamily genes, including *PCDHA9*, which has been identified as a candidate gene for ALS (Zhong et al., 2024).

In summary, the combined analysis of all 25 RNAseq datasets from C9orf72-ALS and isogenic microglia, differentiated from different iPSC lines using different methods, identified several upregulated genes implicated in immune mechanisms and modulation of inflammasome/pyroptosis processes. These include genes encoding cell membrane proteins (CD14, CSFR1, PTPRC/CD45, and SLAMF8), ECM components (BGN, CYR61/CCN1, ITGB6, POSTN, SIGLEC1/CD169, and VSIG4), intracellular proteins (IRAK4, PIK3CG, and FPGT), nuclear transcription factors (CIITA), intracellular proteins secreted under inflammatory conditions (S100A9 and S100B), and proteins with broad intracellular distribution (CAVIN1/PTRF). Importantly, previous studies have shown that the protein products of most of these genes cross-interact at molecular and/or cellular levels in several tissues, supporting the notion that at least some of them participate in common mechanisms in microglia (Table 1). Taken together, the present results have characterized cell-autonomous changes in the transcriptome of C9orf72-ALS iPSC-derived microglia, consistent across different experimental conditions, providing evidence for dysregulation of mechanisms involved in NLRP3 inflammasome activation and ECM biology.

**Table 1.** NLRP3 Inflammasome-associated differentially expressed genes.

**DEG**	**Protein localization**	**Interaction/correlation with other DEGs**	**Involved in TLR/NF-κB signalling**
CD14	Transmembrane	BGN, CSF1R (Roedig et al., 2019; Zheng et al., 2023)	YES (Roedig et al., 2019; Zhao et al., 2010)
CSF1R	Transmembrane	CD14 (Zheng et al., 2023)	
PTPRC/CD45	Transmembrane	POSTN, S110B (Rai et al., 2016; Yan et al., 2015)	YES (Puck et al., 2021)
SLAMF8	Transmembrane	CD14 (Zeng et al., 2020)	YES (Qin et al., 2022; Zeng et al., 2020)
BGN	ECM	CD14, S100A9 (Hou et al., 2014; Roedig et al., 2019;)	YES (Roedig et al., 2019)
CYR61/CCN1	ECM	CIITA, IRAK4 (Lee et al., 2010; Mooring et al., 2023)	YES (Jun & Lau, 2020)
ITGB6	ECM	PIK3CG (Duggan et al., 2024)	
POSTN	ECM	PTPRC (Rai et al., 2016)	YES (Zhu et al., 2024)
SIGLEC1/CD169	ECM		YES (Li et al., 2020)
VSIG4	ECM	CIITA (Kim et al., 2005)	YES (Wang et al., 2022a; Zhang et al., 2021)
IRAK4	Intracellular	CD14, CYR61 (Mooring et al., 2023; Sayers et al., 2024)	YES (Kim et al., 2007**;** 2019)
PIK3CG	Intracellular	ITGB6 (Duggan et al., 2024)	
S100A9	Intracell/Secreted	BGN (Hou et al., 2014)	YES (Hermani et al., 2006; Riva et al., 2012)
S100B	Intracell/Secreted	PTPRC (Yan et al., 2015)	YES (Bianchi et al., 2010; Villarreal et al., 2011)
CIITA	Nucleus	CYR61, VSIG4 (Kim et al., 2005; Lee et al., 2010)	YES (Benson & Ernst, 2009; Qian et al., 2018)
CAVIN1/PTRF	Nucleus, caveolae		YES (Yi et al., 2021; Zheng et al., 2013)

Shown are the localizations of the encoded proteins (ECM, extracellular matrix), molecular interactions and/or expression correlations among different.

DEGs, and involvement in mechanisms regulating TLR and NF-κB signalling.

### Effect of C9orf72-ALS iPSC-Derived Microglia Conditioned Medium on Motor Neurons

**Figure 5.**
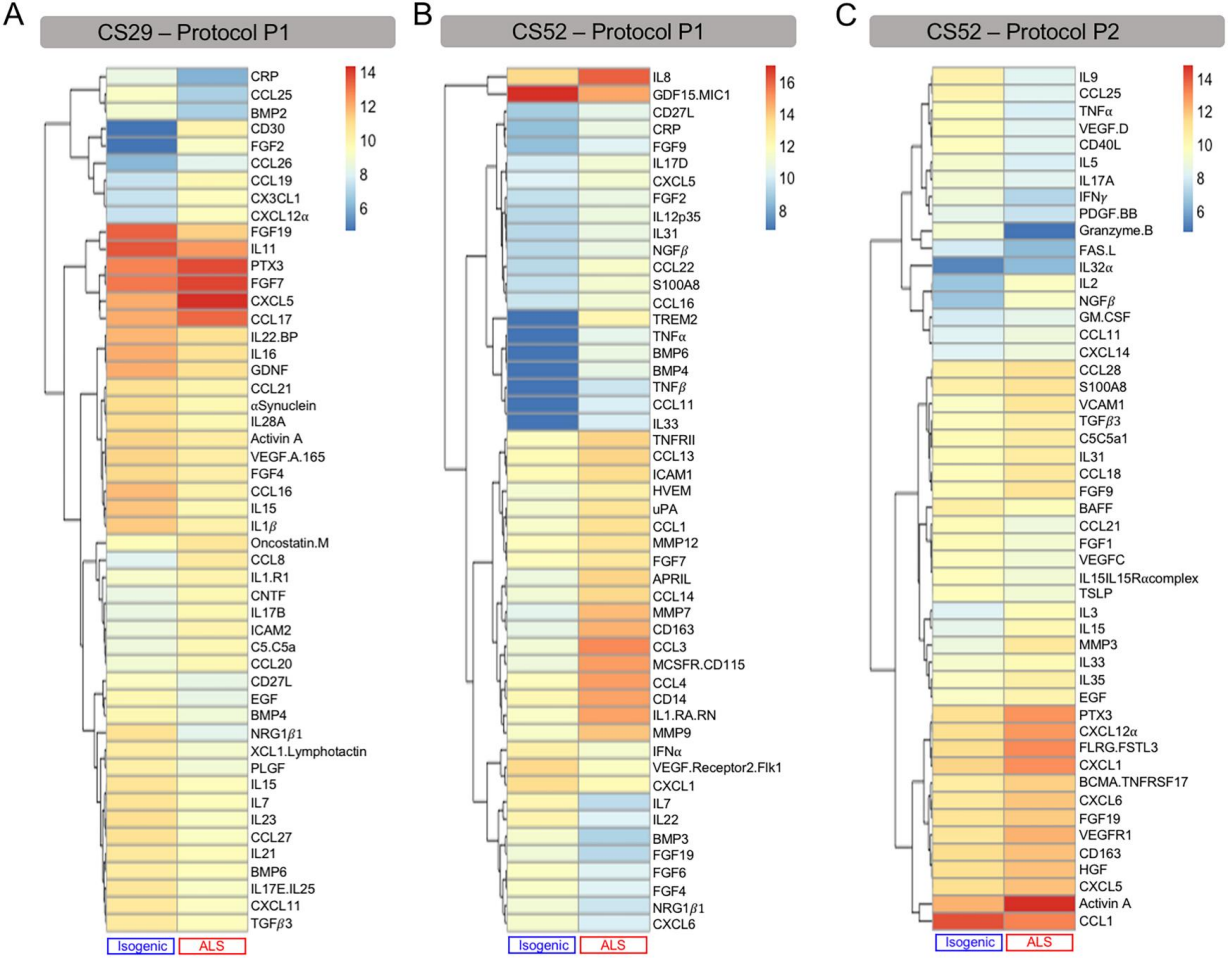
Differentially secreted proteins in C9orf72-ALS microglia identified by multiplex immunoassay. (A-C) Heatmaps showing the top 50 most differentially secreted proteins in CS29 (A) or CS52 (B) iPSC-derived microglia cultures differentiated with protocol P1, and CS52 iPSC-derived microglia cultures derived with protocol P2 (C). Levels of 180 secreted proteins in cell culture supernatants were measured using the nELISA bead-based multiplex immunoassay platform. For each heatmap, columns correspond to isogenic or C9orf72-ALS microglia samples and each line corresponds to one protein. Subgroups of proteins with correlated level patterns are portrayed by the dendrogram on the left side of each heatmap. Relative levels of proteins are indicated by a color scale, with cool colors corresponding to low secreted levels and warm colors corresponding to high secreted levels.

The cell-autonomous transcriptomic changes observed in C9orf72-ALS microglia suggest that these cells might have a dysregulated pro-inflammatory secretome. To examine this possibility, we characterized the levels of 180 proteins in the conditioned media from C9orf72-ALS microglia preparations obtained using protocols P1 and P2. Among the top 50 dysregulated proteins, we observed up-regulated levels of several pro-inflammatory members of the CCL, CXCL, and matrix metalloprotease (MMP) families in the secretome of both CS52-C9n6-M and CS29-C9n1-M microglia using both differentiation protocols. These over-secreted proteins included molecules associated with the NLRP3 inflammasome that matched up-regulated DEGs from our RNAseq studies, such as *CCL3*, *CCL4*, *CCL13*, *CXCL5*, *CXCL12*, *MMP9*, and *MMP12* (Figure 5A–C and Supplemental Information). Activation of innate immunity mechanisms in C9orf72-ALS microglia was also suggested by the dysregulated secretion of other immunity-mediating molecules, including CD163, CD14, S100A8, and PENTRAXIN3 (PTX3) (Figure 5A–C and Supplemental Information). Several of these dysregulated proteins are known to have neuroinflammatory effects, including CCL3, CXCL5, CXCL12, MMP9, MMP12, and S100A8/A9 dimers (Li et al., 2019; 2024; Liu et al., 2013; 2024b; Shi et al., 2025; Vahsen et al., 2023; Wei et al., 2025).

**Figure 6.**
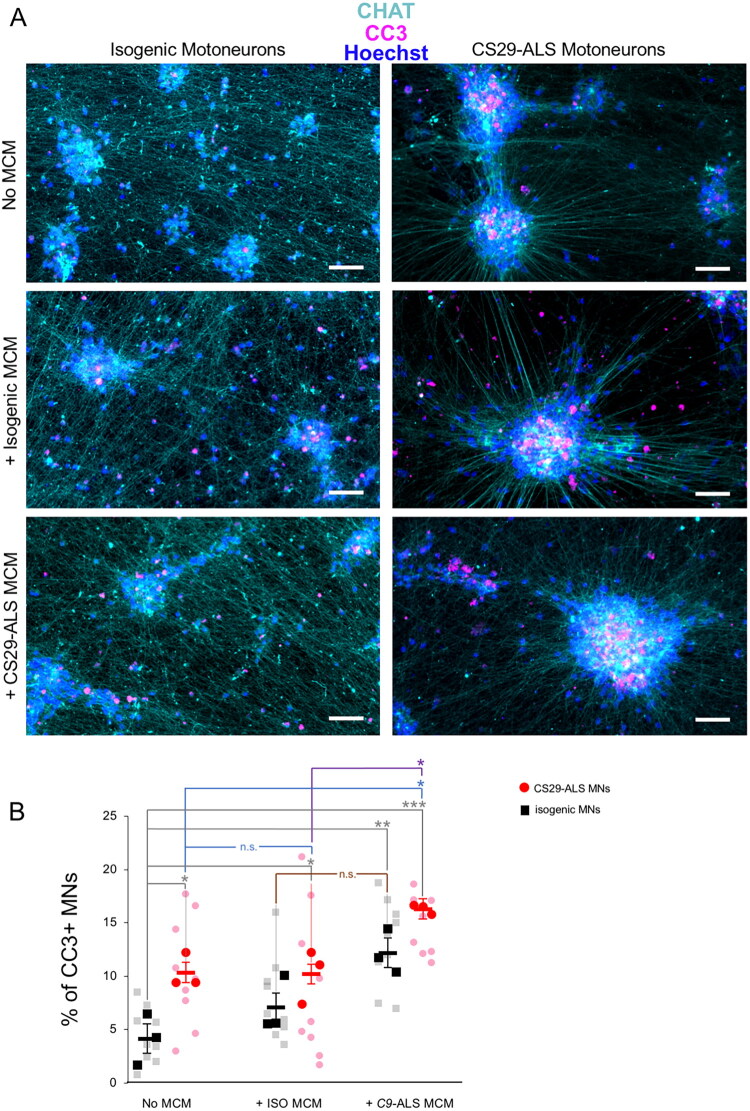
Effect of C9orf72-ALS microglia conditioned media on C9orf72-ALS motor neurons. (A) Representative immunocytochemistry images of motor neurons (Motoneurons) derived from isogenic (left column) or C9orf72-ALS (CS29-ALS) (right column) iPSCs and maintained in culture for 3 weeks in the absence of microglia conditioned media (MCM) (top images: No MCM) or in the presence of conditioned media from isogenic microglia (middle images: isogenic MCM) or C9orf72-ALS (CS29-ALS) MCM (bottom images). Cultures were immunostained with anti-CC3 antibody (magenta), anti-CHAT antibody (cyan), and counterstained with Hoechst (blue). Scale bars, 50 µm. (B) Plot showing the quantification of the percentage of apoptotic CC3-positive motor neurons in isogenic or CS29 motor neuron preparations after 3 weeks of culture in each of the following three conditions: absence of MCM (No MCM); presence of isogenic microglia conditioned media (ISO MCM); or presence of CS29 MCM (C9-ALS MCM). Statistics were conducted at the level of n = 3 biological replicates per group and per condition (black squares for Isogenic MNs; red dots for ALS MNs). For each biological replicate, the percentages were computed on three images of >300 cells in three random fields (grey squares for Isogenic MNs; pink dots for ALS MNs). Two-way ANOVA and Tukey’s multiple comparisons post-test; n.s. = non-significant; * = p value < 0.05; **p value < 0.005; ***p value < 0.0005.

Both RNAseq and secretome data suggest that C9orf72-ALS microglia are neuroinflammatory. To directly test this possibility, we examined the effect of conditioned media from either C9orf72-ALS microglia or their matching isogenic controls on both C9orf72-ALS and isogenic motor neurons. Mutated or isogenic motor neurons were differentiated for 25 days, followed by addition of either no conditioned media or conditioned media collected from C9orf72-ALS or isogenic microglia generated using protocol P1. Motor neurons were then cultured for an additional three weeks (till day 46) and then subjected to immunocytochemistry with an antibody against cleaved caspase 3 (CC3) to detect apoptotic cells (Figure 6A). Motor neurons were visualized with an antibody against choline acetyltransferase (CHAT). With no conditioned media added, quantification of the numbers of CHAT-expressing cells positive for CC3 showed increased numbers of apoptotic C9orf72-ALS motor neurons compared to isogenic motor neurons (Figure 6B, bars 1 and 2) indicating intrinsic reduced viability of the mutant cells, in agreement with previous studies (Thiry et al., 2024). Exposure to conditioned media from isogenic microglia had no detectable effect on the viability of either C9orf72-ALS or isogenic motor neurons (Figure 6B, bars 1–4). In contrast, we observed a statistically significant increase in the fraction of CC3-positive C9orf72-ALS motor neurons in the presence of conditioned media from C9orf72-ALS microglia compared to absence of any conditioned media or exposure to media from isogenic microglia (Figure 6B, cf. bars 2, 4, 6). Isogenic motor neuron cultures tended to contain more CC3-positive cells in the presence of conditioned media from C9orf72-ALS microglia, but this was not statistically significant when compared to conditioned media from isogenic microglia (Figure 6B, cf. bars 3 and 5). These results provide evidence that C9orf72-ALS microglia enhance the death of C9orf72-ALS motor neurons and that this effect does not require motor neuron-microglial cell contact.

## Discussion

There is evidence from studies in animal and human cell models of ALS that the *C9orf72* HRE is associated with activation of the NLRP3 inflammasome in microglia (Banerjee et al., 2023; Fu et al., 2023; Rivers-Auty et al., 2024; Trageser et al., 2023). Little information is available, however, about the molecular mechanisms underlying the activation of inflammasome-mediated processes in C9orf72-ALS microglia. In this study, we sought to identify cell-intrinsic gene and protein expression changes in *C9orf72*-HRE microglia that might shed light on the molecular events underlying inflammasome activation in C9orf72-ALS microglia and how this is correlated with neurodegeneration.

The present RNAseq investigations have identified changes in the expression of several genes suggesting possible mechanisms of NLRP3 inflammasome activation in C9orf72-ALS microglia. For instance, DEG *SLAMF8* codes for a cell surface receptor that is increased in collagen-induced rheumatoid arthritis and knockout of *SLAMF8* attenuates this condition through inhibition of TLR4/NF-κB signaling (Qin et al., 2022). Consistently, simultaneous knockout of *SLAMF8* and its related family member *SLAMF9* prevents LPS-induced liver inflammation by downregulating TLR4 expression on macrophages (Zeng et al., 2020). These findings suggest that abnormally elevated levels of SLAMF8 in C9orf72-ALS microglia might play a role in initiating inflammation and/or maintaining a pro-inflammatory environment via TLR4/NF-κB-mediated activation of the NLRP3 inflammasome. Another candidate activator of the NLRP3 inflammasome is the product of DEG *CD14*, which is a GPI-anchored co-receptor for TLRs involved in LPS-induced responses. CD14/TLR coreceptors are important for microglia activation in ALS, as demonstrated by the finding that microglia activation mediated by extracellular mutant superoxide dismutase 1 was attenuated using TLR2, TLR4 and CD14 blocking antibodies, or when microglia lacked CD14 expression (Zhao et al., 2010). Thus, elevated levels of CD14 in C9orf72-ALS microglia could cause increased TLR signaling, with the potential to activate the NLRP3 inflammasome via NF-κB. A role for CD14 in dysregulated TLR activation in C9orf72-ALS microglia is also suggested by the present identification of *BGN* as an upregulated gene in these cells. The *BGN* gene product, biglycan (BGN), is an ECM-derived damage-associated molecular pattern that acts as a high affinity ligand for CD14 in macrophages, where CD14 is required for BGN-mediated TLR2/4-dependent inflammatory signaling pathways (Roedig et al., 2019). Consistently, BGN can activate the NLRP3 inflammasome in macrophages leading to activation of CASP1 and release of IL1B (Babelova et al., 2009). These observations suggest that elevated levels of both BGN and CD14 cause dysregulated NLRP3 inflammasome activation through elevated TLR/NF-κB signaling.

The present study has identified other ECM protein-encoding genes, in addition to *BGN*, among DEGs in C9orf72-ALS microglia with the potential to activate inflammasome-mediated mechanisms. More specifically, the matricellular protein CYR61/CCN1, encoded by DEG *CYR61*, acts as a pattern recognition receptor that binds bacterial pathogen-associated molecular patterns in macrophages, where it interacts directly with TLR2 and TLR4 to activate inflammatory responses (Jun & Lau, 2020). In agreement with this finding, CYR61/CCN1 promotes the expression of NLRP3, ASC, CASP1, and IL1B in astrocytes in an autocrine manner (Jin et al., 2024). Separate investigations showed that knockdown of the present DEG, *POSTN*, encoding periostin (POSTN), a secreted matricellular protein, suppresses CASP1-mediated pyroptosis and NLRP3 inflammasome activation in muscle cells (Yao et al., 2020). Consistently, POSTN activates inflammation in atopic dermatitis and modulates the immune response and ECM remodeling during atherosclerotic plaque formation (Nunomura et al., 2023; Schwanekamp et al., 2016). Relevant to these observations, POSTN enhances integrin/NF-κB signaling during the process of intervertebral disc degeneration (Zhu et al., 2024). Although not among the top 30 DEGs found in our datasets, numerous additional ECM genes were present among the statistically significant genes dysregulated in C9orf72-ALS microglia, consistent with alterations in ECM composition. Together, these observations suggest that the *C9orf72* HRE causes dysregulation of mechanisms that may underlie an interplay between ECM biology and inflammatory responses in microglia. This notion is consistent with the previous observation of a link between NLRP3 inflammasome/pyroptosis and ECM remodelling in non-neural systems (He et al., 2023), further suggesting a connection between dysregulated inflammasome activation and the ECM in C9orf72-ALS microglia.

Our results also suggest intracellular mechanisms by which the NLRP3 inflammasome may be stimulated in C9orf72-ALS microglia. Interleukin 1 Receptor Associated Kinase 4, encoded by DEG *IRAK4*, is a kinase that activates NF-κB downstream of TLR signaling. Mice lacking *IRAK4* display severe impairment of TLR signaling because of the loss of the normal physical association of the cytoplasmic portion of TLRs with IRAK4 (Suzuki et al., 2002). Consistently, inhibition of IRAK4/NF-κB/NLRP3 pathway reduces pyroptosis in hippocampal neurons (Zhao et al., 2024). Thus, it is reasonable to speculate that upregulation of IRAK4 in C9orf72-ALS microglia could contribute to NLRP3 inflammasome activation, especially if occurring together with other dysregulated mechanisms. Another potential process by which the NLRP3 inflammasome could become activated in C9orf72-ALS microglia through enhanced TLR signaling is suggested by the present identification of *S100B* and *S100A9* (and the related *S100A8* gene) as upregulated DEGs. These genes encode intracellular proteins containing EF-hand calcium-binding motifs. During inflammation, S100A9 and S100B proteins are released in the extracellular milieu where they can bind to TLRs and Receptor for Advanced Glycation End-products (RAGE) (Franz et al., 2022; Villarreal et al., 2014). In microglia, increased level of extracellular S100B upregulates IL1B and TNF-α expression via RAGE engagement and this effect requires activation of NF-κB (Bianchi et al., 2010). Extracellular S100B can promote reactive astrocyte transformation leading to a pro-inflammatory phenotype characterized by expression of TLR2 and IL1B (Villarreal et al., 2014). Consistently, extracellular S100A9 stimulates macrophages to undergo NLRP3 inflammasome activation (Sheng et al., 2025). Conversely, ablation of S100A9 in macrophages dampens NLRP3-driven inflammatory response and pyroptosis (Sheng et al., 2023). Of note, *S100A9* expression is downregulated when NLRP3 inflammasome blockade leads to reduced inflammation and ECM remodeling in adipose tissue (Unamuno et al., 2021). Taken together, these observations suggest several mechanisms by which the upregulated genes identified in C9orf72-ALS microglia in this study could be involved in NLRP3 inflammasome activation.

The cell autonomous pro-inflammatory phenotype of C9orf72-ALS microglia suggests that these cells can be toxic to motor neurons. In agreement with this possibility, the present studies have shown that C9orf72-ALS microglia have a dysregulated secretome characterized by over-secretion of several cytokine/chemokines with demonstrated neuroinflammatory activity, including but not limited to CCL3, CXCL5, CXCL12, MMP9, MMP12, and S100A8/A9 dimers. We therefore examined the possibility that factors released by C9orf72-ALS microglia are sufficient to decrease the survival of C9orf72-ALS motor neurons in the absence of cell-cell contact. Our results have shown that exposure to conditioned media from C9orf72-ALS microglia, but not isogenic microglia, increases the number of apoptotic C9orf72-ALS motor neurons. The effect of the C9orf72-ALS microglia conditioned media on isogenic motor neurons was only moderate. This finding agrees with the results of previous studies showing that LPS-primed C9orf72-ALS iPSC-derived microglia promote the death of motor neurons in microglia-motor neuron co-culture studies (Banerjee et al., 2023; Vahsen et al., 2023). These combined findings provide evidence that C9orf72-ALS microglia have a dysregulated secretome that makes them deleterious to motor neurons in a cell-autonomous fashion. The characterization of the identity and biological features of the neurotoxic molecules secreted by C9orf72-ALS microglia remain to be determined, requiring functional validation of candidate molecules through biochemical and genetic studies.

In conclusion, the transcriptomic and secretome changes in C9orf72-ALS microglia described in this study provide evidence for a cell-autonomous pro-inflammatory phenotype associated with this mutation. Moreover, they agree with earlier evidence of NLRP3 inflammasome activation in C9orf72-ALS microglia and provide previously unavailable information on the identity of several genes that could contribute to inflammasome activation in microglia and ensuing microglia-mediated neuroinflammation in ALS/FTD. At least some of the mechanisms mediated by these gene products could provide targets for translational research.

## Materials and Methods

### Human Induced Pluripotent Stem Cells

Four previously studied (Chi et al., 2023; Gao et al., 2024; Ho et al., 2021; Thiry et al., 2024) human iPSC lines were used for microglia derivation: the C9orf72-ALS iPSC lines CS52iALS-C9n6 (CS52-C9n6-M) and CS29iALS-C9n1 (CS29-C9n1-M), and their matching isogenic control lines CS52iALS-C9n6.ISOxx (CS52-C9n6-ISO) and CS29iALS-C9n1.ISOxx (CS29-C9n1-ISO). These iPSC lines were obtained from Cedars-Sinai (Los Angeles, CA, USA). Undifferentiated state of human iPSCs was assessed by testing for expression of the stem cell markers NANOG and OCT4 using rabbit anti-NANOG (1/1,000; Abcam; Cambridge, UK; Cat. No. ab21624) and rabbit anti-OCT4 (1 μg/ml; Abcam; Cat. No. ab19857) or goat anti-OCT3/4 (1/500; Santa Cruz Biotechnology; Dallas, TX, USA, Cat. No. sc-8628) antibodies, as previously described (Chen et al., 2021).

### Derivation of Microglia from Human iPSCs

Derivation of microglia from human iPSCs was performed essentially as described in three separate protocols published by Fossati and coworkers (Douvaras et al., 2017), Blurton-Jones and colleagues (McQuade et al., 2018), and Cowley and colleagues (Haenseler et al., 2017). Since we used slightly modified versions of these methods, they are referred to as protocols “P1”, “P2” and “P3,” respectively. The modifications made to the Douvaras et al. and McQuade et al. methods were previously described (Tang et al., 2022). The following modifications were made to the Haenseler et al. method. Floating progenitor cells were harvested from embryoid bodies weekly and cultured by plating onto 6-well plates or glass coverslips coated with Corning® Matrigel (MilliporeSigma; Burlington, MA, USA; Cat. No. 354277) at 100,000 per cm^2^. Cells were cultured for 10–20 days in DMEM/F12 (Thermo-Fisher Scientific; Waltham, MA, USA; Cat. No. 10565-018) with 100 ng/mL IL-34 (Thermo-Fisher Scientific; Cat. No. 200-34), 10 ng/mL GM-CSF (R&D Systems; Minneapolis, MN, USA; Cat. No. 215-GM), N2 supplement (Thermo-Fisher Scientific; Cat. No. 17502-048), 2 mM GlutaMAX (Thermo-Fisher Scientific; Cat. No. 35050-061), and antibiotic-antimycotic (Thermo-Fisher Scientific; Cat. No. 15240-062). All microglia preparations generated using these three protocols were validated through morphological analysis and immunocytochemistry as described (Tang et al., 2022) before being subjected to RNAseq.

### RNA Sequencing

Total RNA was isolated for RNAseq from cell pellets obtained from microglia generated with each one of the three protocols described above using sequential treatment with TRIzol Reagent (ThermoFisher Scientific; Cat. No. 15596026) and PureLink RNA Micro Scale Kit (ThermoFisher Scientific; Cat. No. 12183-016) following the instructions provided by the manufacturer. We isolated RNA from iPSC-derived microglia at a series of time points spanning days *in vitro* 45 to 66 for preparations generated with protocols P1 and P2 and days *in vitro* 75 to 108 for preparations induced with protocol P3. These time points were long enough for induced cells to acquire a microglia phenotype according to all three derivation methods (Douvaras et al., 2017; Haenseler et al., 2017; McQuade et al., 2018). All RNA samples were analyzed by Illumina next-generation sequencing at the Genomics platform at the Institute for Research in Immunology and Cancer, Montreal, Quebec, Canada (https://www.iric.ca/en/research/platforms-andinfrastructures/genomics). Adaptor sequences and low-quality bases in the resulting FASTQ files were trimmed using Trimmomatic version 0·35 (Bolger et al., 2014), and genome alignments were conducted using STAR version 2·5 1b (Dobin et al., 2013). Sequences were aligned to the human genome version GRCh38, with gene annotations from Gencode v29 based on Ensemble release 94. As part of quality control, the sequences were aligned to several different genomes to verify that there was no sample contamination. Raw read-counts were obtained directly from STAR and reads in transcripts per million (TPM) formats were computed using RSEM (Li & Dewey, 2011).

### In Silico Analysis

To compare isogenic microglia preparations derived with three different protocols, heatmaps showing the levels of expression of 49 microglia/macrophage marker genes (Butovsky et al., 2014; Hickman et al., 2013; Muffat et al., 2016; Zhang et al., 2014) were generated for each sample. Read counts for these specific genes were converted into log2-counts-per-million (logCPM) values as previously described (Tang et al., 2022). Accordingly, negative values represent very low gene expression values. Hierarchical clustering of rows was applied to the heatmaps to group genes with correlated expression patterns, resulting in different gene lists, depending on the samples analyzed in the given heatmap. For each heatmap, color comparisons can be made across samples for each gene as well as between genes for any given sample.

Differential gene expression analysis was conducted on the raw read-count matrix using an edgeR (version 4·0) pipeline as previously described (Chen et al., 2016). Briefly, genes were filtered to retain only those with a robust expression level. A scaling factor of the trimmed mean of M-values (TMM) between each pair of samples was used to normalize the differing RNA composition of each sample. The differentiation protocol and experimental batch were incorporated into the design used for differential expression analysis since they were the two known sources of variability in the studied samples. The “glmQLFit” and “glmQLFTest” functions of edgeR were then used to obtain DEGs with the quasi-likelihood (QL) method. Genes were considered statistically significant if the adjusted p-value (false discovery rate; FDR) was <0.05, with no fold-change cutoff applied to restrict the number of DEGs identified.

Gene set enrichment analysis was conducted on the DEGs using the Functional Annotation Tool of DAVID Bioinformatics Resources 6·8 (Huang da et al., 2009a, 2009b). GO terms and KEGG pathways enriched in the DEGs were computed separately for upregulated and downregulated genes, as this increases the statistical power to identify pathways/terms pertinent to the phenotype of interest (Hong et al., 2014). For each GO category (e.g., Biological Process or Molecular Function), the “direct” function in DAVID was used to restrict the results to those directly annotated by the source database and to avoid repetitive and generic parent terms. For the KEGG pathways, only the non-disease-specific pathways are shown. For both GO and KEGG pathways analysis, all terms with associated FRD < 1 are shown, except for categories presenting more than 10 terms, in which case only the more statistically significant terms with associated FDR < e^−6^ are shown.

### Open-Source Data

Raw read counts from RNA sequencing of undifferentiated human iPSCs were obtained from the LINCS Data Portal (LSC-1002 and LSC-1004 from Dataset LDS-1355) (http://lincsportal.ccs.miami.edu/datasets/). LSC-1002 and LSC-1004 correspond to human iPSC lines CS14i-CTR-n6 and CS25iCTR-18n2, provided by Cedar Sinai Stem Cell Core Laboratory. These raw data were combined with the RNAseq data from the present microglia preparations and analyzed using a single pipeline, to generate heatmaps comparing the levels of expression of activated microglia marker genes in each sample.

### Secretome Analysis of Microglia Conditioned Media

Conditioned media from microglia preparations were subjected to analysis of 180 secreted proteins utilising the nucleobase Enabled Localized Immunoassay with Spectral Addressing (nELISA) platform from Nomic Bio (Montreal, Quebec, Canada; https://www.nomic.bio/) (Dagher et al., 2025). The nELISA pre-assembles antibody pairs on spectrally encoded microparticles (Dagher et al., 2018), resulting in spatial separation between non-cognate antibodies, preventing the rise of reagent-driven cross-reactivity, and enabling multiplexing of hundreds of ELISAs in parallel using flow cytometry. Standard curves were generated for each protein using the recombinant protein standard run in each of the control media, enabling quantification of pg/mL concentrations.

### Exposure of Cultures of iPSC-Derived Motor Neurons to Microglia Conditioned Media

Motor neuron cultures enriched in phrenic motor neurons were derived from human iPSC lines CS29-C9n1-M and its matching isogenic control line CS29-C9n1-ISO according to a previously described protocol (Thiry et al., 2024). Briefly, neural progenitor cells were dissociated on day 6 and split 1:5 with a chemically defined neural medium including DMEM/F12 supplemented with GlutaMAX (1/1; Thermo-Fisher Scientific; Cat. No. 35050-061), Neurobasal medium (1/1; Thermo-Fisher Scientific; Cat. No. 21103-049), N2 (0.5X; Thermo-Fisher Scientific; Cat. No. 17504-044), B27 (0.5X; Thermo-Fisher Scientific; Cat. No. 17502-048), ascorbic acid (100 μM; Sigma-Aldrich; Oakville, ON, Canada; Cat. No. A5960), antibiotic-antimycotic (1X; Thermo-Fisher Scientific; Cat. No. 15240-062), supplemented with retinoic acid (RA) (1 µM, Sigma-Aldrich; Cat. No. R2625) and purmorphamine (0.125 µM, Sigma-Aldrich; Cat. No. SML-0868) in combination with 1 μM CHIR99021 (STEMCELL Technologies; Vancouver, BC, Canada; Cat. No. 72054), 2 μM DMH1 (Sigma-Aldrich; Cat. No. D8946), and 2 μM SB431542 (Tocris Bioscience; Bristol, UK; Cat. No. 1614). The culture medium was changed every other day for 6 days and the resulting motor neuron progenitors were then expanded for 6 days with the same medium containing 3 µM CHIR99021, 2 µM DMH1, 2 µM SB431542, 1 µM RA, 0.125 µM purmorphamine, and 500 µM valproic acid (Sigma-Aldrich; Cat. No. P4543). Motor neuron progenitors were dissociated and plated at 50,000 cells/well on coverslips coated with Dendritic Polyglycerol Amine/Matrigel substrate as described previously (Thiry et al., 2022). Motor neurons were differentiated in the same neural medium supplemented with 1 µM RA and 0.125 µM purmorphamine, 0.1 µM Compound E (MilliporeSigma; Cat. No. 565790), insulin-like growth factor 1 (10 ng/mL; R&D Systems; Cat. No. 291-G1-200), brain-derived neurotrophic factor (10 ng/mL; Thermo-Fisher Scientific; Cat. No. PHC7074) and ciliary neurotrophic factor (10 ng/mL; R&D Systems; Cat. No. 257-NT-050). Motor neuron culture medium was replaced every other day for one week. Motor neuron cultures were validated by immunocytochemistry and single-cell RNA sequencing as described (Thiry et al., 2020, 2022, 2024). For studies with microglia conditioned media, after one week in motor neuron culture medium, the medium was replaced every other day for an additional three weeks with a mixture of motor neuron culture medium (50%) and microglia conditioned medium (50%) obtained from either C9orf72-ALS or isogenic microglia derived from CS29-C9n1-M or CS29-C9n1-ISO iPSCs using protocol P2, as described above. After 46 days of culture (3-weeks post-plating), culture media were removed, and motor neurons were rinsed and analyzed by immunocytochemistry.

### Analysis of Apoptosis in iPSC-Derived Motor Neuron Cultures

Immunocytochemistry with rabbit anti-Cleaved-Caspase 3 (CC3 (Asp175); 1/400; Cell Signaling; New England Biolabs, Ltd., Ontario, Canada; Cat. No. 9661S) in combination with goat anti-CHAT antibody (1/100; Millipore; Cat. No. MAB144P) was performed to detect apoptotic motor neurons in cultures obtained from CS29-C9n1-M or CS29-C9n1-ISO iPSCs, in the absence or presence of conditioned media from CS29-C9n1-M or CS29-C9n1-ISO microglia. For quantification, images in 3 random fields were taken with a 20X objective using an Axio Observer Z1 microscope connected to an AxioCam camera using ZEN software (Zeiss) and analyzed with Image J.

### Statistical Analysis of Motor Neuron Cell Death Assays

To account for culture variability and ensure experimental reproducibility, studies with motor neurons plus or minus microglia conditioned media were performed with at least 3 biologically independent cultures per condition. Error bars shown are means ± standard error of means (SEM) of the average. Two-way repeated measure ANOVA and Tukey’s multiple comparisons post-hoc test were used to detect differences in the proportion of CC3-positive motor neurons in isogenic and C9orf72-ALS cells in each of the three culture conditions tested, namely absence of microglia conditioned media, presence of isogenic microglia conditioned media, or presence of C9orf72-ALS microglia conditioned media.

## Supplementary Material

Revised Supplementary Figures and Information_anonymous.pdf

## Data Availability

R code used for in silico analysis was based on the published code (Tang et al., 2022). The raw data are available at the Sequence Read Archive (SRA): http://www.ncbi.nlm.nih.gov/bioproject/1235672.

## References

[CIT0001] Appel, S. H., Beers, D. R., & Zhao, W. (2021). Amyotrophic lateral sclerosis is a systemic disease: Peripheral contributions to inflammation-mediated neurodegeneration. *Current Opinion in Neurology*, *34*(5), 765–772. 10.1097/WCO.0000000000000983 34402459

[CIT0002] Arterbery, A. S., Yao, J., Ling, A., Avitzur, Y., Martinez, M., Lobritto, S., Deng, Y., Geliang, G., Mehta, S., Wang, G., Knight, J., & Ekong, U. D. (2018). Inflammasome Priming Mediated *via* Toll-Like Receptors 2 and 4, Induces Th1-Like Regulatory T Cells in *De Novo* Autoimmune Hepatitis. *Frontiers in Immunology*, *9*, 1612. 10.3389/fimmu.2018.01612 30072988 PMC6060440

[CIT0003] Atanasio, A., Decman, V., White, D., Ramos, M., Ikiz, B., Lee, H. C., Siao, C. J., Brydges, S., LaRosa, E., Bai, Y., Fury, W., Burfeind, P., Zamfirova, R., Warshaw, G., Orengo, J., Oyejide, A., Fralish, M., Auerbach, W., Poueymirou, W., … Lai, K. M. (2016). C9orf72 ablation causes immune dysregulation characterized by leukocyte expansion, autoantibody production, and glomerulonephropathy in mice. *Scientific Reports*, *6*(1), 23204. 10.1038/srep23204 26979938 PMC4793236

[CIT0004] Babelova, A., Moreth, K., Tsalastra-Greul, W., Zeng-Brouwers, J., Eickelberg, O., Young, M. F., Bruckner, P., Pfeilschifter, J., Schaefer, R. M., Gröne, H. J., & Schaefer, L. (2009). Biglycan, a danger signal that activates the NLRP3 inflammasome via toll-like and P2X receptors. *The Journal of Biological Chemistry*, *284*(36), 24035–24048. 10.1074/jbc.M109.014266 19605353 PMC2781998

[CIT0005] Bai, T., Chen, C. C., & Lau, L. F. (2010). Matricellular protein CCN1 activates a proinflammatory genetic program in murine macrophages. *Journal of Immunology (Baltimore, MD.: 1950)*, *184*(6), 3223–3232. 10.4049/jimmunol.0902792 20164416 PMC2832719

[CIT0006] Balendra, R., & Isaacs, A. M. (2018). C9orf72-mediated ALS and FTD: Multiple pathways to disease. *Nature Reviews. Neurology*, *14*(9), 544–558. 10.1038/s41582-018-0047-2 30120348 PMC6417666

[CIT0007] Banerjee, P., Mehta, A. R., Nirujogi, R. S., Cooper, J., James, O. G., Nanda, J., Longden, J., Burr, K., McDade, K., Salzinger, A., Paza, E., Newton, J., Story, D., Pal, S., Smith, C., Alessi, D. R., Selvaraj, B. T., Priller, J., & Chandran, S. (2023). Cell-autonomous immune dysfunction driven by disrupted autophagy in *C9orf72*-ALS iPSC-derived microglia contributes to neurodegeneration. *Science Advances*, *9*(16), eabq0651. 10.1126/sciadv.abq0651 37083530 PMC10121169

[CIT0008] Barnett, K. C., Li, S., Liang, K., & Ting, J. P. (2023). A 360° view of the inflammasome: Mechanisms of activation, cell death, and diseases. *Cell*, *186*(11), 2288–2312. 10.1016/j.cell.2023.04.025 37236155 PMC10228754

[CIT0009] Beers, D. R., & Appel, S. H. (2019). Immune dysregulation in amyotrophic lateral sclerosis: Mechanisms and emerging therapies. *The Lancet. Neurology*, *18*(2), 211–220. 10.1016/S1474-4422(18)30394-6 30663610

[CIT0010] Beers, D. R., Thonhoff, J. R., Faridar, A., Thome, A. D., Zhao, W., Wen, S., & Appel, S. H. (2022). Tregs attenuate peripheral oxidative stress and acute phase proteins in ALS. *Annals of Neurology*, *92*(2), 195–200. 10.1002/ana.26375 35445431 PMC9545429

[CIT0011] Benatar, M., Wuu, J., Huey, E. D., McMillan, C. T., Petersen, R. C., Postuma, R., McHutchison, C., Dratch, L., Arias, J. J., Crawley, A., Houlden, H., McDermott, M. P., Cai, X., Thakur, N., Boxer, A., Rosen, H., Boeve, B. F., Dacks, P., Cosentino, S., … Turner, M. R, Attendees of the Second International Pre-Symptomatic ALS Workshop. (2024). The miami framework for ALS and related neurodegenerative disorders: An integrated view of phenotype and biology. *Nature Reviews. Neurology*, *20*(6), 364–376. Epub 2024 May 20. 10.1038/s41582-024-00961-z 38769202 PMC11216694

[CIT0012] Benson, S. A., & Ernst, J. D. (2009). TLR2-dependent inhibition of macrophage responses to IFN-gamma is mediated by distinct, gene-specific mechanisms. *PloS One*, *4*(7), e6329. 10.1371/journal.pone.0006329 19629181 PMC2710511

[CIT0013] Bi, J., Dai, J., Koivisto, L., Larjava, M., Bi, L., Häkkinen, L., & Larjava, H. (2019). Inflammasome and cytokine expression profiling in experimental periodontitis in the integrin β6 null mouse. *Cytokine*, *114*, 135–142. 10.1016/j.cyto.2018.11.011 30467097

[CIT0014] Bianchi, R., Giambanco, I., & Donato, R. (2010). S100B/RAGE-dependent activation of microglia via NF-kappaB and AP-1 co-regulation of COX-2 expression by S100B, IL-1beta and TNF-alpha. *Neurobiology of Aging*, *31*(4), 665–677. 10.1016/j.neurobiolaging.2008.05.017 18599158

[CIT0015] Bolger, A. M., Lohse, M., & Usadel, B. (2014). Trimmomatic: A flexible trimmer for Illumina sequence data. *Bioinformatics (Oxford, England)*, *30*(15), 2114–2120. 10.1093/bioinformatics/btu170 24695404 PMC4103590

[CIT0016] Burberry, A., Suzuki, N., Wang, J. Y., Moccia, R., Mordes, D. A., Stewart, M. H., Suzuki-Uematsu, S., Ghosh, S., Singh, A., Merkle, F. T., Koszka, K., Li, Q. Z., Zon, L., Rossi, D. J., Trowbridge, J. J., Notarangelo, L. D., & Eggan, K. (2016). Loss-of-function mutations in the C9ORF72 mouse ortholog cause fatal autoimmune disease. *Science Translational Medicine*, *8*(347), 347ra93. 10.1126/scitranslmed.aaf6038 PMC5024536 27412785

[CIT0017] Butovsky, O., Jedrychowski, M. P., Moore, C. S., Cialic, R., Lanser, A. J., Gabriely, G., Koeglsperger, T., Dake, B., Wu, P. M., Doykan, C. E., Fanek, Z., Liu, L., Chen, Z., Rothstein, J. D., Ransohoff, R. M., Gygi, S. P., Antel, J. P., & Weiner, H. L. (2014). Identification of a unique TGF-β-dependent molecular and functional signature in microglia. *Nature Neuroscience*, *17*(1), 131–143. 10.1038/nn.3599 24316888 PMC4066672

[CIT0018] Cai, X., Liang, X., Wang, K., Liu, Y., Hao, M., Li, H., Dai, X., & Ding, L. (2022). Pyroptosis-related lncRNAs: A novel prognosis signature of colorectal cancer. *Frontiers in Oncology*, *12*, 983895. 10.3389/fonc.2022 36531020 PMC9748486

[CIT0019] Calma, A. D., Pavey, N., Menon, P., & Vucic, S. (2024). Neuroinflammation in amyotrophic lateral sclerosis: Pathogenic insights and therapeutic implications. *Current Opinion in Neurology*, *37*(5), 585–592. 10.1097/WCO.0000000000001279 38775138

[CIT0020] Cardenas, A. M., Sarlls, J. E., Kwan, J. Y., Bageac, D., Gala, Z. S., Danielian, L. E., Ray-Chaudhury, A., Wang, H. W., Miller, K. L., Foxley, S., Jbabdi, S., Welsh, R. C., & Floeter, M. K. (2017). Pathology of callosal damage in ALS: An *ex-vivo*, 7T diffusion tensor MRI study. *NeuroImage. Clinical*, *15*, 200–208. 10.1016/j.nicl.2017.04.024 28529876 PMC5429246

[CIT0021] Chen, C. X., Abdian, N., Maussion, G., Thomas, R. A., Demirova, I., Cai, E., Tabatabaei, M., Beitel, L. K., Karamchandani, J., Fon, E. A., & Durcan, T. M. (2021). A multistep workflow to evaluate newly generated iPSCs and their ability to generate different cell types. *Methods and Protocols*, *4*(3), 50. 10.3390/mps4030050 34287353 PMC8293472

[CIT0022] Chen, M., Geoffroy, C. G., Meves, J. M., Narang, A., Li, Y., Nguyen, M. T., Khai, V. S., Kong, X., Steinke, C. L., Carolino, K. I., Elzière, L., Goldberg, M. P., Jin, Y., & Zheng, B. (2018). Leucine zipper-bearing kinase is a critical regulator of astrocyte reactivity in the adult mammalian CNS. *Cell Reports*, *22*(13), 3587–3597. 10.1016/j.celrep.2018.02.102 29590625 PMC5905706

[CIT0023] Chen, Y., Lun, A. T. L., & Smyth, G. K. (2016). From reads to genes to pathways: Differential expression analysis of RNA-Seq experiments using Rsubread and the edgeR quasi-likelihood pipeline. *F1000Research*, *5*, 1438. 10.12688/f1000research.8987.2 27508061 PMC4934518

[CIT0024] Chew, J., Gendron, T. F., Prudencio, M., Sasaguri, H., Zhang, Y. J., Castanedes-Casey, M., Lee, C. W., Jansen-West, K., Kurti, A., Murray, M. E., Bieniek, K. F., Bauer, P. O., Whitelaw, E. C., Rousseau, L., Stankowski, J. N., Stetler, C., Daughrity, L. M., Perkerson, E. A., Desaro, P., … Petrucelli, L. (2015). Neurodegeneration. C9ORF72 repeat expansions in mice cause TDP-43 pathology, neuronal loss, and behavioral deficits. *Science (New York, N.Y.)*, *348*(6239), 1151–1154. 10.1126/science.aaa9344 25977373 PMC4692360

[CIT0025] Chi, B., Öztürk, M. M., Paraggio, C. L., Leonard, C. E., Sanita, M. E., Dastpak, M., O’Connell, J. D., Coady, J. A., Zhang, J., Gygi, S. P., Lopez-Gonzalez, R., Yin, S., & Reed, R. (2023). Causal ALS genes impact the MHC class II antigen presentation pathway. *Proceedings of the National Academy of Sciences of the United States of America*, *120*(39), e2305756120. 10.1073/pnas.2305756120 37722062 PMC10523463

[CIT0026] Dagher, M., Kleinman, M., Ng, A., & Juncker, D. (2018). Ensemble multicolour FRET model enables barcoding at extreme FRET levels. *Nature Nanotechnology*, *13*(10), 925–932. 10.1038/s41565-018-0205-0 30061659

[CIT0027] Dagher, M., Ongo, G., Robichaud, N., Kong, J., Rho, W., Teahulos, I., Tavakoli, A., Bovaird, S., Merjaneh, S., Tan, A., Edwardson, K., Scheepers, C., Ng, A., Hajjar, A., Sow, B., Vrouvides, M., Lee, A., DeCorwin-Martin, P., Rasool, S., … Juncker, D. (2025). nELISA: A high-throughput, high-plex platform enables quantitative profiling of the inflammatory secretome. *bioRxiv: The Preprint Server for Biology*, bioRxiv [Preprint] Mar 24:20230417535914. 10.1101/2023.04.17.535914 PMC12615254 41203862

[CIT0028] de Calbiac, H., Renault, S., Haouy, G., Jung, V., Roger, K., Zhou, Q., Campanari, M. L., Chentout, L., Demy, D. L., Marian, A., Goudin, N., Edbauer, D., Guerrera, C., Ciura, S., & Kabashi, E. (2024). Poly-GP accumulation due to C9orf72 loss of function induces motor neuron apoptosis through autophagy and mitophagy defects. *Autophagy*, *20*(10), 2164–2185. 10.1080/15548627.2024.2358736 39316747 PMC11423671

[CIT0030] DeJesus-Hernandez, M., Mackenzie, I. R., Boeve, B. F., Boxer, A. L., Baker, M., Rutherford, N. J., Nicholson, A. M., Finch, N. A., Flynn, H., Adamson, J., Kouri, N., Wojtas, A., Sengdy, P., Hsiung, G. Y., Karydas, A., Seeley, W. W., Josephs, K. A., Coppola, G., Geschwind, D. H., … Rademakers, R. (2011). Expanded GGGGCC hexanucleotide repeat in noncoding region of C9ORF72 causes chromosome 9p-linked FTD and ALS. *Neuron*, *72*(2), 245–256. 10.1016/j.neuron.2011.09.011 21944778 PMC3202986

[CIT0031] Dobin, A., Davis, C. A., Schlesinger, F., Drenkow, J., Zaleski, C., Jha, S., Batut, P., Chaisson, M., & Gingeras, T. R. (2013). STAR: Ultrafast universal RNA-seq aligner. *Bioinformatics (Oxford, England)*, *29*(1), 15–21. 10.1093/bioinformatics/bts635 23104886 PMC3530905

[CIT0032] Dong, X., Zhang, Y., Sun, Y., Nan, Q., Li, M., Ma, L., Zhang, L., Luo, J., Qi, Y., & Miao, Y. (2023). Promoter hypermethylation and comprehensive regulation of ncRNA lead to the down-regulation of ZNF880, providing a new insight for the therapeutics and research of colorectal cancer. *BMC Medical Genomics*, *16*(1), 148. 10.1186/s12920-023-01571-2 37370088 PMC10294494

[CIT0033] Donnelly, C. J., Zhang, P. W., Pham, J. T., Haeusler, A. R., Mistry, N. A., Vidensky, S., Daley, E. L., Poth, E. M., Hoover, B., Fines, D. M., Maragakis, N., Tienari, P. J., Petrucelli, L., Traynor, B. J., Wang, J., Rigo, F., Bennett, C. F., Blackshaw, S., Sattler, R., & Rothstein, J. D. (2013). RNA toxicity from the ALS/FTD C9ORF72 expansion is mitigated by antisense intervention. *Neuron*, *80*(2), 415–428. 10.1016/j.neuron.2013.10.015 24139042 PMC4098943

[CIT0034] Douvaras, P., Sun, B., Wang, M., Kruglikov, I., Lallos, G., Zimmer, M., Terrenoire, C., Zhang, B., Gandy, S., Schadt, E., Freytes, D. O., Noggle, S., & Fossati, V. (2017). Directed differentiation of human pluripotent stem cells to microglia. *Stem Cell Reports*, *8*(6), 1516–1524. 10.1016/j.stemcr.2017.04.023 28528700 PMC5470097

[CIT0035] Du, X., Xu, Y., Chen, S., & Fang, M. (2020). Inhibited CSF1R alleviates ischemia injury via inhibition of microglia M1 polarization and NLRP3 pathway. *Neural Plasticity*, *2020*, 8825911–8825954. 10.1155/2020/8825954 PMC7474788 32908485

[CIT0036] Duggan, M. R., Peng, Z., Sipilä, P. N., Lindbohm, J. V., Chen, J., Lu, Y., Davatzikos, C., Erus, G., Hohman, T. J., Andrews, S. J., Candia, J., Tanaka, T., Joynes, C. M., Alvarado, C. X., Nalls, M. A., Cordon, J., Daya, G. N., An, Y., Lewis, A., … Walker, K. A. (2024). Proteomics identifies potential immunological drivers of postinfection brain atrophy and cognitive decline. *Nature Aging*, *4*(9), 1263–1278. Epub 2024 Aug 14. 10.1038/s43587-024-00682-4 39143319 PMC11408246

[CIT0037] Franz, S., Ertel, A., Engel, K. M., Simon, J. C., & Saalbach, A. (2022). Overexpression of S100A9 in obesity impairs macrophage differentiation via TLR4-NFkB-signaling worsening inflammation and wound healing. *Theranostics*, *12*(4), 1659–1682. 10.7150/thno.67174 35198063 PMC8825590

[CIT0038] Freeman, L., Guo, H., David, C. N., Brickey, W. J., Jha, S., & Ting, J. P. (2017). NLR members NLRC4 and NLRP3 mediate sterile inflammasome activation in microglia and astrocytes. *The Journal of Experimental Medicine*, *214*(5), 1351–1370. 10.1084/jem.20150237 28404595 PMC5413320

[CIT0039] Fu, J., & Wu, H. (2023). Structural mechanisms of NLRP3 inflammasome assembly and activation. *Annual Review of Immunology*, *41*(1), 301–316. 10.1146/annurev-immunol-081022-021207 PMC10159982 36750315

[CIT0040] Fu, R. H., Chen, H. J., & Hong, S. Y. (2023). Glycine-alanine dipeptide repeat protein from C9-ALS interacts with sulfide quinone oxidoreductase (SQOR) to induce the activity of the NLRP3 Inflammasome in HMC3 Microglia: Irisflorentin reverses this interaction. *Antioxidants (Basel, Switzerland)*, *12*(10), 1896. 10.3390/antiox12101896 37891975 PMC10604625

[CIT0041] Gao, C., Shi, Q., Pan, X., Chen, J., Zhang, Y., Lang, J., Wen, S., Liu, X., Cheng, T. L., & Lei, K. (2024). Neuromuscular organoids model spinal neuromuscular pathologies in C9orf72 amyotrophic lateral sclerosis. *Cell Reports*, *43*(3), 113892. 10.1016/j.celrep.2024.113892 38431841

[CIT0042] Gendron, T. F., & Petrucelli, L. (2018). Disease mechanisms of *C9ORF72* repeat expansions. *Cold Spring Harbor Perspectives in Medicine*, *8*(4), a024224. 10.1101/cshperspect.a024224 28130314 PMC5880161

[CIT0043] Goutman, S. A., Hardiman, O., Al-Chalabi, A., Chió, A., Savelieff, M. G., Kiernan, M. C., & Feldman, E. L. (2022). Emerging insights into the complex genetics and pathophysiology of amyotrophic lateral sclerosis. *The Lancet. Neurology*, *21*(5), 465–479. 10.1016/S1474-4422(21)00414-2 35334234 PMC9513754

[CIT0044] Grottelli, S., Mezzasoma, L., Scarpelli, P., Cacciatore, I., Cellini, B., & Bellezza, I. (2019). Cyclo(His-Pro) inhibits NLRP3 inflammasome cascade in ALS microglial cells. *Molecular and Cellular Neurosciences*, *94*, 23–31. 10.1016/j.mcn.2018.11.002 30439413

[CIT0045] Gu, L., Chen, H., Geng, R., Liang, T., Chen, Y., Wang, Z., Ye, L., Sun, M., Shi, Q., Wan, G., Chang, J., Wei, J., Ma, W., Xiao, J., Bao, X., & Wang, R. (2024). Endothelial pyroptosis-driven microglial activation in choroid plexus mediates neuronal apoptosis in hemorrhagic stroke rats. *Neurobiology of Disease*, *201*, 106695. 10.1016/j.nbd.2024.106695 39370051

[CIT0047] Haage, V., Semtner, M., Vidal, R. O., Hernandez, D. P., Pong, W. W., Chen, Z., Hambardzumyan, D., Magrini, V., Ly, A., Walker, J., Mardis, E., Mertins, P., Sauer, S., Kettenmann, H., & Gutmann, D. H. (2019). Comprehensive gene expression meta-analysis identifies signature genes that distinguish microglia from peripheral monocytes/macrophages in health and glioma. *Acta Neuropathologica Communications*, *7*(1), 20. 10.1186/s40478-019-0665-y 30764877 PMC6376799

[CIT0048] Haenseler, W., Sansom, S. N., Buchrieser, J., Newey, S. E., Moore, C. S., Nicholls, F. J., Chintawar, S., Schnell, C., Antel, J. P., Allen, N. D., Cader, M. Z., Wade-Martins, R., James, W. S., & Cowley, S. A. (2017). A highly efficient human pluripotent stem cell microglia model displays a neuronal-co-culture-specific expression profile and inflammatory response. *Stem Cell Reports*, *8*(6), 1727–1742. 10.1016/j.stemcr.2017.05.017 28591653 PMC5470330

[CIT0049] He, A., Liu, Y., Zhang, R., Mao, Y., & Liu, W. (2023). CircSFMBT2-OA alleviates chondrocyte apoptosis and extracellular matrix degradation through repressing NF-κB/NLRP3 inflammasome activation. *Heliyon*, *9*(6), e17312. 10.1016/j.heliyon.2023.e17312 37441407 PMC10333456

[CIT0050] Hermani, A., De Servi, B., Medunjanin, S., Tessier, P. A., & Mayer, D. (2006). S100A8 and S100A9 activate MAP kinase and NF-kappaB signaling pathways and trigger translocation of RAGE in human prostate cancer cells. *Experimental Cell Research*, *312*(2), 184–197. 10.1016/j.yexcr.2005.10.013 16297907

[CIT0051] Hickman, S. E., Kingery, N. D., Ohsumi, T. K., Borowsky, M. L., Wang, L. C., Means, T. K., & El Khoury, J. (2013). The microglial sensome revealed by direct RNA sequencing. *Nature Neuroscience*, *16*(12), 1896–1905. 10.1038/nn.3554 24162652 PMC3840123

[CIT0052] Ho, R., Workman, M. J., Mathkar, P., Wu, K., Kim, K. J., O’Rourke, J. G., Kellogg, M., Montel, V., Banuelos, M. G., Arogundade, O. A., Diaz-Garcia, S., Oheb, D., Huang, S., Khrebtukova, I., Watson, L., Ravits, J., Taylor, K., Baloh, R. H., & Svendsen, C. N. (2021). Cross-comparison of human iPSC motor neuron models of familial and sporadic ALS reveals early and convergent transcriptomic disease signatures. *Cell Systems*, *12*(2), 159–175.e9. 10.1016/j.cels.2020.10.010 33382996 PMC7897311

[CIT0053] Hong, G., Zhang, W., Li, H., Shen, X., & Guo, Z. (2014). Separate enrichment analysis of pathways for up- and downregulated genes. *Journal of the Royal Society, Interface*, *11*(92), 20130950. 10.1098/rsif.2013.0950 24352673 PMC3899863

[CIT0054] Hou, A., Lan, W., Law, K. P., Khoo, S. C., Tin, M. Q., Lim, Y. P., & Tong, L. (2014). Evaluation of global differential gene and protein expression in primary Pterygium: S100A8 and S100A9 as possible drivers of a signaling network. *PLoS One*, *9*(5), e97402. 10.1371/journal.pone.0097402 24825356 PMC4019582

[CIT0055] Hsiao, C. C., Sankowski, R., Prinz, M., Smolders, J., Huitinga, I., & Hamann, J. (2021). GPCRomics of homeostatic and disease-associated human microglia. *Frontiers in Immunology*, *12*, 674189. 10.3389/fimmu.2021.674189 34054860 PMC8160299

[CIT0056] Huang da, W., Sherman, B. T., & Lempicki, R. A. (2009a). Systematic and integrative analysis of large gene lists using DAVID bioinformatics resources. *Nature Protocols*, *4*(1), 44–57. 10.1038/nprot.2008.211 19131956

[CIT0057] Huang da, W., Sherman, B. T., & Lempicki, R. A. (2009b). Bioinformatics enrichment tools: Paths toward the comprehensive functional analysis of large gene lists. *Nucleic Acids Research*, *37*(1), 1–13. 10.1093/nar/gkn923 19033363 PMC2615629

[CIT0058] Huang, X., Feng, Z., Jiang, Y., Li, J., Xiang, Q., Guo, S., Yang, C., Fei, L., Guo, G., Zheng, L., Wu, Y., & Chen, Y. (2019). VSIG4 mediates transcriptional inhibition of *Nlrp3* and *Il-1*β in macrophages. *Science Advances*, *5*(1), eaau7426. 10.1126/sciadv.aau7426 30662948 PMC6326752

[CIT0059] Ip, W. K., & Medzhitov, R. (2015). Macrophages monitor tissue osmolarity and induce inflammatory response through NLRP3 and NLRC4 inflammasome activation. *Nature Communications*, *6*(1), 6931. 10.1038/ncomms7931 PMC4430126 25959047

[CIT0060] Izrael, M., Slutsky, S. G., & Revel, M. (2020). Rising Stars: Astrocytes as a Therapeutic target for ALS disease. *Frontiers in Neuroscience*, *14*, 824. 10.3389/fnins.2020.00824 32848579 PMC7399224

[CIT0061] Ji, H., Liu, Z., Wang, N., Jin, J., Zhang, J., Dong, J., Wang, F., Yan, X., Gong, Q., Zhao, H., Sun, H., Li, Y., Hu, S., & You, C. (2022). Integrated genomic, transcriptomic, and epigenetic analyses identify a leukotriene synthesis-related M2 macrophage gene signature that predicts prognosis and treatment vulnerability in gliomas. *Frontiers in Immunology*, *13*, 970702. 10.3389/fimmu.2022.970702 36159811 PMC9493442

[CIT0062] Jin, D., Dai, Z., Zhao, L., Ma, T., Ma, Y., & Zhang, Z. (2024). CYR61 is involved in neonatal hypoxic-ischemic brain damage via modulating astrocyte-mediated neuroinflammation. *Neuroscience*, *552*, 54–64. 10.1016/j.neuroscience.2024.06.001 38908506

[CIT0063] Jun, J. I., & Lau, L. F. (2020). CCN1 is an opsonin for bacterial clearance and a direct activator of toll-like receptor signaling. *Nature Communications*, *11*(1), 1242. 10.1038/s41467-020-15075-5 PMC7060279 32144270

[CIT0064] Kim, J. K., Choi, E. M., Shin, H. I., Kim, C. H., Hwang, S. H., Kim, S. M., & Kwon, B. S. (2005). Characterization of monoclonal antibody specific to the Z39Ig protein, a member of immunoglobulin superfamily. *Immunology Letters*, *99*(2), 153–161. 10.1016/j.imlet.2005.02.012 16009265

[CIT0065] Kim, T. W., Staschke, K., Bulek, K., Yao, J., Peters, K., Oh, K. H., Vandenburg, Y., Xiao, H., Qian, W., Hamilton, T., Min, B., Sen, G., Gilmour, R., & Li, X. (2007). A critical role for IRAK4 kinase activity in toll-like receptor-mediated innate immunity. *The Journal of Experimental Medicine*, *204*(5), 1025–1036. 10.1084/jem.20061825 17470642 PMC2118590

[CIT0066] Kim, Y. C., Lee, S. E., Kim, S. K., Jang, H. D., Hwang, I., Jin, S., Hong, E. B., Jang, K. S., & Kim, H. S. (2019). Toll-like receptor mediated inflammation requires FASN-dependent MYD88 palmitoylation. *Nature Chemical Biology*, *15*(9), 907–916. 10.1038/s41589-019-0344-0 31427815

[CIT0067] Kirola, L., Mukherjee, A., & Mutsuddi, M. (2022). Recent updates on the genetics of amyotrophic lateral sclerosis and frontotemporal dementia. *Molecular Neurobiology*, *59*(9), 5673–5694. 10.1007/s12035-022-02934-z 35768750

[CIT0068] Lajqi, T., Lang, G. P., Haas, F., Williams, D. L., Hudalla, H., Bauer, M., Groth, M., Wetzker, R., & Bauer, R. (2019). Memory-like inflammatory responses of microglia to rising doses of LPS: Key role of PI3Kγ. *Frontiers in Immunology*, *10*, 2492. 10.3389/fimmu.2019.02492 31781091 PMC6856213

[CIT0069] Lall, D., Lorenzini, I., Mota, T. A., Bell, S., Mahan, T. E., Ulrich, J. D., Davtyan, H., Rexach, J. E., Muhammad, A. K. M. G., Shelest, O., Landeros, J., Vazquez, M., Kim, J., Ghaffari, L., O’Rourke, J. G., Geschwind, D. H., Blurton-Jones, M., Holtzman, D. M., Sattler, R., & Baloh, R. H. (2021). C9orf72 deficiency promotes microglial-mediated synaptic loss in aging and amyloid accumulation. *Neuron*, *109*(14), 2275–2291.e8. 10.1016/j.neuron.2021.05.020 34133945 PMC8298293

[CIT0070] Lant, S. B., Robinson, A. C., Thompson, J. C., Rollinson, S., Pickering-Brown, S., Snowden, J. S., Davidson, Y. S., Gerhard, A., & Mann, D. M. (2014). Patterns of microglial cell activation in frontotemporal lobar degeneration. *Neuropathology and Applied Neurobiology*, *40*(6), 686–696. 10.1111/nan.12092 24117616

[CIT0071] Leaf, I. A., Nakagawa, S., Johnson, B. G., Cha, J. J., Mittelsteadt, K., Guckian, K. M., Gomez, I. G., Altemeier, W. A., & Duffield, J. S. (2017). Pericyte MyD88 and IRAK4 control inflammatory and fibrotic responses to tissue injury. *The Journal of Clinical Investigation*, *127*(1), 321–334. 10.1172/JCI87532 27869651 PMC5199713

[CIT0072] Lee, Y. L., Lin, S. K., Hong, C. Y., Wang, J. S., Yang, H., Lai, E. H., Chen, M. H., & Kok, S. H. (2010). Major histocompatibility complex class II transactivator inhibits cysteine-rich 61 expression in osteoblastic cells and its implication in the pathogenesis of periapical lesions. *Journal of Endodontics*, *36*(6), 1021–1025. 10.1016/j.joen.2010.03.009 20478458

[CIT0073] Li, B., & Dewey, C. N. (2011). RSEM: Accurate transcript quantification from RNA-Seq data with or without a reference genome. *BMC Bioinformatics*, *12*(1), 323. 10.1186/1471-2105-12-323 21816040 PMC3163565

[CIT0074] Li, D., Yu, L., Zi, J., Du, X., Yan, X., Chen, H., Wang, L., Zheng, C., Wang, G., Zhang, J., & Jiang, Y. (2025). SLAMF8 disrupts epithelial barrier in chronic rhinosinusitis with nasal polyps via M1 macrophage polarization. *Ann Allergy Asthma Immunol*, *25* S1081-1206(25)00047-X. 10.1016/j.anai.2025.01.020 39870212

[CIT0075] Li, L., Zhou, J., Han, L., Guo, C., Ma, S., Ge, S., & Qu, Y. (2024). Intervention of CXCL5 attenuated neuroinflammation and promoted neurological recovery after traumatic brain injury. *Neuroreport*, *35*(9), 549–557. 10.1097/WNR.0000000000002032 38739900

[CIT0076] Li, S., Jiang, L., Yang, Y., Cao, J., Zhang, Q., Zhang, J., Wang, R., Deng, X., & Li, Y. (2020). Siglec1 enhances inflammation through miR-1260-dependent degradation of IκBα in COPD. *Experimental and Molecular Pathology*, *113*, 104398. 10.1016/j.yexmp.2020.104398 32007531

[CIT0077] Li, Y., Niu, M., Zhao, A., Kang, W., Chen, Z., Luo, N., Zhou, L., Zhu, X., Lu, L., & Liu, J. (2019). CXCL12 is involved in α-synuclein-triggered neuroinflammation of Parkinson’s disease. *Journal of Neuroinflammation*, *16*(1), 263. 10.1186/s12974-019-1646-6 31831012 PMC6909602

[CIT0078] Lin, Y. H., Yu, J., Teng, Y. C., Huang, C. G., Lim, S. N., Lai, M. W., & Lin, W. R. (2025). The ZNF717-rs2918520 genotype contributes to COVID-19 severity: A Taiwanese cohort study. *BMC Infectious Diseases*, *25*(1), 201. 10.1186/s12879-025-10551-z 39934654 PMC11817965

[CIT0079] Liu, X., Wang, J., Jin, J., Hu, Q., Zhao, T., Wang, J., Gao, J., & Man, J. (2024b). S100A9 deletion in microglia/macrophages ameliorates brain injury through the STAT6/PPARγ pathway in ischemic stroke. *CNS Neuroscience & Therapeutics*, *30*(8), e14881. 10.1111/cns.14881 39107960 PMC11303267

[CIT0080] Liu, Y., Ma, T. X., Fan, P. F., Wang, Z., Wang, Z., & Li, L. (2024a). STAT3-induced lncRNA GNAS-AS1 accelerates keloid formation by mediating the miR-196a-5p/CXCL12/STAT3 axis in a feedback loop. *Experimental Dermatology*, *33*(6), e15111. 10.1111/exd.15111 38840411

[CIT0081] Liu, Y., Zhang, M., Hao, W., Mihaljevic, I., Liu, X., Xie, K., Walter, S., & Fassbender, K. (2013). Matrix metalloproteinase-12 contributes to neuroinflammation in the aged brain. *Neurobiology of Aging*, *34*(4), 1231–1239. 10.1016/j.neurobiolaging.2012.10.015 23159549

[CIT0082] Lopez-Gonzalez, R., Lu, Y., Gendron, T. F., Karydas, A., Tran, H., Yang, D., Petrucelli, L., Miller, B. L., Almeida, S., & Gao, F. B. (2016). Poly(GR) in C9ORF72-related ALS/FTD compromises mitochondrial function and increases oxidative stress and dna damage in iPSC-derived motor neurons. *Neuron*, *92*(2), 383–391. 10.1016/j.neuron.2016.09.015 27720481 PMC5111366

[CIT0083] Lorenzini, I., Alsop, E., Levy, J., Gittings, L. M., Lall, D., Rabichow, B. E., Moore, S., Pevey, R., Bustos, L. M., Burciu, C., Bhatia, D., Singer, M., Saul, J., McQuade, A., Tzioras, M., Mota, T. A., Logemann, A., Rose, J., Almeida, S., … Sattler, R. (2023). Moderate intrinsic phenotypic alterations in *C9orf72*ALS/FTD iPSC-microglia despite the presence of C9orf72 pathological features. *Frontiers in Cellular Neuroscience*, *17*, 1179796. 10.3389/fncel.2023.1179796 37346371 PMC10279871

[CIT0084] Lu, C., Liu, J., Escames, G., Yang, Y., Wu, X., Liu, Q., Chen, J., Song, Y., Wang, Z., Deng, C., Acuña-Castroviejo, D., & Wang, X. (2023). PIK3CG regulates NLRP3/GSDMD-mediated pyroptosis in septic myocardial injury. *Inflammation*, *46*(6), 2416–2432. 10.1007/s10753-023-01889-0 37676465

[CIT0085] Majewski, S., Klein, P., Boillée, S., Clarke, B. E., & Patani, R. (2025). Towards an integrated approach for understanding glia in amyotrophic lateral sclerosis. *Glia*, *73*(3), 591–607. 10.1002/glia.24622 39318236 PMC11784848

[CIT0086] Martínez-Muriana, A., Mancuso, R., Francos-Quijorna, I., Olmos-Alonso, A., Osta, R., Perry, V. H., Navarro, X., Gomez-Nicola, D., & López-Vales, R. (2016). CSF1R blockade slows the progression of amyotrophic lateral sclerosis by reducing microgliosis and invasion of macrophages into peripheral nerves. *Scientific Reports*, *6*(1), 25663. 10.1038/srep25663 27174644 PMC4865981

[CIT0087] Masaki, M., Ikeda, A., Shiraki, E., Oka, S., & Kawasaki, T. (2003). Mixed lineage kinase LZK and antioxidant protein-1 activate NF-kappaB synergistically. *European Journal of Biochemistry*, *270*(1), 76–83. 10.1046/j.1432-1033.2003.03363.x 12492477

[CIT0088] McQuade, A., Coburn, M., Tu, C. H., Hasselmann, J., Davtyan, H., & Blurton-Jones, M. (2018). Development and validation of a simplified method to generate human microglia from pluripotent stem cells. *Molecular Neurodegeneration*, *13*(1), 67. 10.1186/s13024-018-0297-x 30577865 PMC6303871

[CIT0089] Mehta, A. R., Gregory, J. M., Dando, O., Carter, R. N., Burr, K., Nanda, J., Story, D., McDade, K., Smith, C., Morton, N. M., Mahad, D. J., Hardingham, G. E., Chandran, S., & Selvaraj, B. T. (2021). Mitochondrial bioenergetic deficits in C9orf72 amyotrophic lateral sclerosis motor neurons cause dysfunctional axonal homeostasis. *Acta Neuropathologica*, *141*(2), 257–279. 10.1007/s00401-020-02252-5 33398403 PMC7847443

[CIT0090] Mimic, S., Aru, B., Pehlivanoğlu, C., Sleiman, H., Andjus, P. R., & Yanıkkaya Demirel, G. (2023). Immunology of amyotrophic lateral sclerosis—role of the innate and adaptive immunity. *Frontiers in Neuroscience*, *17*, 1277399. 10.3389/fnins.2023.1277399 38105925 PMC10723830

[CIT0091] Mooring, M., Yeung, G. A., Luukkonen, P., Liu, S., Akbar, M. W., Zhang, G. J., Balogun, O., Yu, X., Mo, R., Nejak-Bowen, K., Poyurovsky, M. V., Booth, C. J., Konnikova, L., Shulman, G. I., & Yimlamai, D. (2023). Hepatocyte CYR61 polarizes profibrotic macrophages to orchestrate NASH fibrosis. *Science Translational Medicine*, *15*(715), eade3157. 10.1126/scitranslmed.ade3157 37756381 PMC10874639

[CIT0092] Muffat, J., Li, Y., Yuan, B., Mitalipova, M., Omer, A., Corcoran, S., Bakiasi, G., Tsai, L. H., Aubourg, P., Ransohoff, R. M., & Jaenisch, R. (2016). Efficient derivation of microglia-like cells from human pluripotent stem cells. *Nature Medicine*, *22*(11), 1358–1367. 10.1038/nm.4189 PMC5101156 27668937

[CIT0093] Nicholson, A. M., Zhou, X., Perkerson, R. B., Parsons, T. M., Chew, J., Brooks, M., DeJesus-Hernandez, M., Finch, N. A., Matchett, B. J., Kurti, A., Jansen-West, K. R., Perkerson, E., Daughrity, L., Castanedes-Casey, M., Rousseau, L., Phillips, V., Hu, F., Gendron, T. F., Murray, M. E., … Rademakers, R. (2018). Loss of Tmem106b is unable to ameliorate frontotemporal dementia-like phenotypes in an AAV mouse model of C9ORF72-repeat induced toxicity. *Acta Neuropathologica Communications*, *6*(1), 42. 10.1186/s40478-018-0545-x 29855382 PMC5984311

[CIT0094] Nijs, M., & Van Damme, P. (2024). The genetics of amyotrophic lateral sclerosis. *Current Opinion in Neurology*, *37*(5), 560–569. 10.1097/WCO.0000000000001294 38967083 PMC11377058

[CIT0095] Nunomura, S., Uta, D., Kitajima, I., Nanri, Y., Matsuda, K., Ejiri, N., Kitajima, M., Ikemitsu, H., Koga, M., Yamamoto, S., Honda, Y., Takedomi, H., Andoh, T., Conway, S. J., & Izuhara, K. (2023). Periostin activates distinct modules of inflammation and itching downstream of the type 2 inflammation pathway. *Cell Reports*, *42*(1), 111933. 10.1016/j.celrep.2022.111933 36610396 PMC11486451

[CIT0096] O’Rourke, J. G., Bogdanik, L., Yáñez, A., Lall, D., Wolf, A. J., Muhammad, A. K., Ho, R., Carmona, S., Vit, J. P., Zarrow, J., Kim, K. J., Bell, S., Harms, M. B., Miller, T. M., Dangler, C. A., Underhill, D. M., Goodridge, H. S., Lutz, C. M., & Baloh, R. H. (2016). C9orf72 is required for proper macrophage and microglial function in mice. *Science (New York, N.Y.)*, *351*(6279), 1324–1329. 10.1126/science.aaf1064 26989253 PMC5120541

[CIT0097] Pappalardo, X. G., Jansen, G., Amaradio, M., Costanza, J., Umeton, R., Guarino, F., De Pinto, V., Oliver, S. G., Messina, A., & Nicosia, G. (2024). Inferring gene regulatory networks of ALS from blood transcriptome profiles. *Heliyon*, *10*(23), e40696. 10.1016/j.heliyon.2024.e40696 39687198 PMC11648123

[CIT0098] Peferoen, L. A., Vogel, D. Y., Ummenthum, K., Breur, M., Heijnen, P. D., Gerritsen, W. H., Peferoen-Baert, R. M., van der Valk, P., Dijkstra, C. D., & Amor, S. (2015). Activation status of human microglia is dependent on lesion formation stage and remyelination in multiple sclerosis. *Journal of Neuropathology and Experimental Neurology*, *74*(1), 48–63. 10.1097/NEN.0000000000000149 25470347

[CIT0099] Pruenster, M., Immler, R., Roth, J., Kuchler, T., Bromberger, T., Napoli, M., Nussbaumer, K., Rohwedder, I., Wackerbarth, L. M., Piantoni, C., Hennis, K., Fink, D., Kallabis, S., Schroll, T., Masgrau-Alsina, S., Budke, A., Liu, W., Vestweber, D., Wahl-Schott, C., … Sperandio, M. (2023). E-selectin-mediated rapid NLRP3 inflammasome activation regulates S100A8/S100A9 release from neutrophils via transient gasdermin D pore formation. *Nature Immunology*, *24*(12), 2021–2031. 10.1038/s41590-023-01656-1 37903858 PMC10681899

[CIT0100] Puck, A., Künig, S., Modak, M., May, L., Fritz, P., Battin, C., Radakovics, K., Steinberger, P., Reipert, B. M., Crowe, B. A., & Stöckl, J. (2021). The soluble cytoplasmic tail of CD45 regulates T-cell activation via TLR4 signaling. *European Journal of Immunology*, *51*(12), 3176–3185. 10.1002/eji.202149227 34626426

[CIT0101] Qian, J., Luo, F., Yang, J., Liu, J., Liu, R., Wang, L., Wang, C., Deng, Y., Lu, Z., Wang, Y., Lu, M., Wang, J. Y., & Chu, Y. (2018). TLR2 promotes glioma immune evasion by downregulating MHC Class II molecules in microglia. *Cancer Immunology Research*, *6*(10), 1220–1233. 10.1158/2326-6066.CIR-18-0020 30131377

[CIT0102] Qin, W., Rong, X., Yu, C., Jia, P., Yang, J., & Zhou, G. (2022). Knockout of SLAMF8 attenuates collagen-induced rheumatoid arthritis in mice through inhibiting TLR4/NF-κB signaling pathway. *International Immunopharmacology*, *107*, 108644. 10.1016/j.intimp.2022.108644 35259711

[CIT0103] Radian, A. D., Khare, S., Chu, L. H., Dorfleutner, A., & Stehlik, C. (2015). ATP binding by NLRP7 is required for inflammasome activation in response to bacterial lipopeptides. *Molecular Immunology*, *67*(2 Pt B), 294–302. 10.1016/j.molimm.2015.06.013 26143398 PMC4565763

[CIT0104] Rai, A. N., Thornton, J. A., Stokes, J., Sunesara, I., Swiatlo, E., & Nanduri, B. (2016). Polyamine transporter in Streptococcus pneumoniae is essential for evading early innate immune responses in pneumococcal pneumonia. *Scientific Reports*, *6*(1), 26964. 10.1038/srep26964 27247105 PMC4887915

[CIT0105] Renton, A. E., Majounie, E., Waite, A., Simón-Sánchez, J., Rollinson, S., Gibbs, J. R., Schymick, J. C., Laaksovirta, H., van Swieten, J. C., Myllykangas, L., Kalimo, H., Paetau, A., Abramzon, Y., Remes, A. M., Kaganovich, A., Scholz, S. W., Duckworth, J., Ding, J., Harmer, D. W., … Traynor, B. J, ITALSGEN Consortium. (2011). A hexanucleotide repeat expansion in C9ORF72 is the cause of chromosome 9p21-linked ALS-FTD. *Neuron*, *72*(2), 257–268. 10.1016/j.neuron.2011.09.010 21944779 PMC3200438

[CIT0106] Riva, M., Källberg, E., Björk, P., Hancz, D., Vogl, T., Roth, J., Ivars, F., & Leanderson, T. (2012). Induction of nuclear factor-κB responses by the S100A9 protein is toll-like receptor-4-dependent. *Immunology*, *137*(2), 172–182. 10.1111/j.1365-2567.2012.03619.x 22804476 PMC3461398

[CIT0107] Rivers-Auty, J., Hoyle, C., Pointer, A., Lawrence, C., Pickering-Brown, S., Brough, D., & Ryan, S. (2024). *C9orf72*dipeptides activate the NLRP3 inflammasome. *Brain Communications*, *6*(5), fcae282. 10.1093/braincomms/fcae282 39229486 PMC11369816

[CIT0108] Roedig, H., Nastase, M. V., Frey, H., Moreth, K., Zeng-Brouwers, J., Poluzzi, C., Hsieh, L. T., Brandts, C., Fulda, S., Wygrecka, M., & Schaefer, L. (2019). Biglycan is a new high-affinity ligand for CD14 in macrophages. *Matrix Biology: Journal of the International Society for Matrix Biology*, *77*, 4–22. 10.1016/j.matbio.2018.05.006 29777767

[CIT0109] Sanfilippo, C., Longo, A., Lazzara, F., Cambria, D., Distefano, G., Palumbo, M., Cantarella, A., Malaguarnera, L., & Di Rosa, M. (2017). CHI3L1 and CHI3L2 overexpression in motor cortex and spinal cord of sALS patients. *Molecular and Cellular Neurosciences*, *85*, 162–169. 10.1016/j.mcn.2017.10.001 28989002

[CIT0110] Schwanekamp, J. A., Lorts, A., Vagnozzi, R. J., Vanhoutte, D., & Molkentin, J. D. (2016). Deletion of periostin protects against atherosclerosis in mice by altering inflammation and extracellular matrix remodeling. *Arteriosclerosis, Thrombosis, and Vascular Biology*, *36*(1), 60–68. 10.1161/ATVBAHA.115.306397 26564821 PMC4690815

[CIT0111] Sharma, B. R., & Kanneganti, T. D. (2021). NLRP3 inflammasome in cancer and metabolic diseases. *Nature Immunology*, *22*(5), 550–559. 10.1038/s41590-021-00886-5 33707781 PMC8132572

[CIT0112] Sheng, M., Liu, W., Cao, Y., Wang, S., Lin, Y., & Yu, W. (2025). Targeting S100A9-TLR2 axis controls macrophage NLRP3 inflammasome activation in fatty liver ischemia reperfusion injury. *Shock (Augusta, Ga.)*, *63*(2), 292–298. 10.1097/SHK.0000000000002470 39447083 PMC11776876

[CIT0113] Sheng, M., Weng, Y., Cao, Y., Zhang, C., Lin, Y., & Yu, W. (2023). Caspase 6/NR4A1/SOX9 signaling axis regulates hepatic inflammation and pyroptosis in ischemia-stressed fatty liver. *Cell Death Discovery*, *9*(1), 106. 10.1038/s41420-023-01396-z 36977670 PMC10043527

[CIT0114] Shi, G., Cao, Y., Xu, J., Chen, B., Zhang, X., Zhu, Y., Liu, L., Liu, X., Zhang, L., Zhou, Y., Li, S., Yang, G., Liu, X., Chen, F., Chen, X., Zhang, J., & Zhang, S. (2025). Inhibition of S100A8/A9 ameliorates neuroinflammation by blocking NET formation following traumatic brain injury. *Redox Biology*, *81*, 103532. 10.1016/j.redox.2025.103532 39929053 PMC11849670

[CIT0115] Shu, X., Wei, C., Tu, W. Y., Zhong, K., Qi, S., Wang, A., Bai, L., Zhang, S. X., Luo, B., Xu, Z. Z., Zhang, K., & Shen, C. (2023). Negative regulation of TREM2-mediated C9orf72 poly-GA clearance by the NLRP3 inflammasome. *Cell Reports*, *42*(2), 112133. 10.1016/j.celrep.2023.112133 36800288

[CIT0116] Souza de Lima, D., Nunes, V. C. L., Ogusku, M. M., Sadahiro, A., Pontillo, A., & Alencar, B. C. (2017). Polymorphisms in SIGLEC1 contribute to susceptibility to pulmonary active tuberculosis possibly through the modulation of IL-1ß. *Infection, Genetics and Evolution: Journal of Molecular Epidemiology and Evolutionary Genetics in Infectious Diseases*, *55*, 313–317. 10.1016/j.meegid.2017.09.031 28964857

[CIT0117] Sudria-Lopez, E., Koppers, M., de Wit, M., van der Meer, C., Westeneng, H. J., Zundel, C. A., Youssef, S. A., Harkema, L., de Bruin, A., Veldink, J. H., van den Berg, L. H., & Pasterkamp, R. J. (2016). Full ablation of C9orf72 in mice causes immune system-related pathology and neoplastic events but no motor neuron defects. *Acta Neuropathologica*, *132*(1), 145–147. 10.1007/s00401-016-1581-x 27206760 PMC4911370

[CIT0118] Suzuki, N., Suzuki, S., Duncan, G. S., Millar, D. G., Wada, T., Mirtsos, C., Takada, H., Wakeham, A., Itie, A., Li, S., Penninger, J. M., Wesche, H., Ohashi, P. S., Mak, T. W., & Yeh, W. C. (2002). Severe impairment of interleukin-1 and toll-like receptor signalling in mice lacking IRAK-4. *Nature*, *416*(6882), 750–756. 10.1038/nature736 11923871

[CIT0119] Tang, Y. M., Pulimood, N. S., & Stifani, S. (2022). Comparing the characteristics of microglia preparations generated using different human iPSC-based differentiation methods to model neurodegenerative diseases. *ASN Neuro*, *14*(1), 17590914221145105. 10.1177/17590914221145105 36524236 PMC9761225

[CIT0120] Thiry, L., Clément, J. P., Haag, R., Kennedy, T. E., & Stifani, S. (2022). Optimization of long-term human iPSC-derived spinal motor neuron culture using a dendritic polyglycerol amine-based substrate. *ASN Neuro*, *14*(1), 17590914211073381. 10.1177/17590914211073381 35023784 PMC8784909

[CIT0121] Thiry, L., Hamel, R., Pluchino, S., Durcan, T., & Stifani, S. (2020). Characterization of human iPSC-derived spinal motor neurons by single-cell RNA sequencing. *Neuroscience*, *450*, 57–70. 10.1016/j.neuroscience.2020.04.041 32380268

[CIT0122] Thiry, L., Sirois, J., Durcan, T. M., & Stifani, S. (2024). Generation of human iPSC-derived phrenic-like motor neurons to model respiratory motor neuron degeneration in ALS. *Communications Biology*, *7*(1), 238. 10.1038/s42003-024-05925-z 38418587 PMC10901792

[CIT0123] Trageser, K. J., Yang, E. J., Smith, C., Iban-Arias, R., Oguchi, T., Sebastian-Valverde, M., Iqbal, U. H., Wu, H., Estill, M., Al Rahim, M., Raval, U., Herman, F. J., Zhang, Y. J., Petrucelli, L., & Pasinetti, G. M. (2023). Inflammasome-mediated neuronal-microglial crosstalk: A therapeutic substrate for the familial C9orf72 variant of frontotemporal dementia/amyotrophic lateral sclerosis. *Molecular Neurobiology*, *60*(7), 4004–4016. 10.1007/s12035-023-03315-w 37010807

[CIT0124] Tran, H., Almeida, S., Moore, J., Gendron, T. F., Chalasani, U., Lu, Y., Du, X., Nickerson, J. A., Petrucelli, L., Weng, Z., & Gao, F. B. (2015). Differential toxicity of nuclear RNA foci versus dipeptide repeat proteins in a drosophila model of C9ORF72 FTD/ALS. *Neuron*, *87*(6), 1207–1214. 10.1016/j.neuron.2015.09.015 26402604 PMC4589299

[CIT0125] Unamuno, X., Gómez-Ambrosi, J., Ramírez, B., Rodríguez, A., Becerril, S., Valentí, V., Moncada, R., Silva, C., Salvador, J., Frühbeck, G., & Catalán, V. (2021). NLRP3 inflammasome blockade reduces adipose tissue inflammation and extracellular matrix remodeling. *Cellular & Molecular Immunology*, *18*(4), 1045–1057. 10.1038/s41423-019-0296-z 31551515 PMC8115140

[CIT0126] Vahsen, B. F., Nalluru, S., Morgan, G. R., Farrimond, L., Carroll, E., Xu, Y., Cramb, K. M. L., Amein, B., Scaber, J., Katsikoudi, A., Candalija, A., Carcolé, M., Dafinca, R., Isaacs, A. M., Wade-Martins, R., Gray, E., Turner, M. R., Cowley, S. A., & Talbot, K. (2023). C9orf72-ALS human iPSC microglia are pro-inflammatory and toxic to co-cultured motor neurons via MMP9. *Nature Communications*, *14*(1), 5898. 10.1038/s41467-023-41603-0 PMC10517114 37736756

[CIT0127] Vasudevan, S. O., Russo, A. J., Kumari, P., Vanaja, S. K., & Rathinam, V. A. (2022). A TLR4-independent critical role for CD14 in intracellular LPS sensing. *Cell Reports*, *39*(5), 110755. 10.1016/j.celrep.2022.110755 35508125 PMC9376664

[CIT0128] Vidmar, L., Maver, A., Drulović, J., Sepčić, J., Novaković, I., Ristič, S., Šega, S., & Peterlin, B. (2019). Multiple Sclerosis patients carry an increased burden of exceedingly rare genetic variants in the inflammasome regulatory genes. *Scientific Reports*, *9*(1), 9171. 10.1038/s41598-019-45598-x 31235738 PMC6591387

[CIT0129] Villarreal, A., Aviles Reyes, R. X., Angelo, M. F., Reines, A. G., & Ramos, A. J. (2011). S100B alters neuronal survival and dendrite extension via RAGE-mediated NF-κB signaling. *Journal of Neurochemistry*, *117*(2), 321–332. 10.1111/j.1471-4159.2011.07207.x 21291473

[CIT0130] Villarreal, A., Seoane, R., González Torres, A., Rosciszewski, G., Angelo, M. F., Rossi, A., Barker, P. A., & Ramos, A. J. (2014). S100B protein activates a RAGE-dependent autocrine loop in astrocytes: Implications for its role in the propagation of reactive gliosis. *Journal of Neurochemistry*, *131*(2), 190–205. 10.1111/jnc.12790 24923428

[CIT0131] Vizuete, A. F. K., Fróes, F., Seady, M., Zanotto, C., Bobermin, L. D., Roginski, A. C., Wajner, M., Quincozes-Santos, A., & Gonçalves, C. A. (2022). Early effects of LPS-induced neuroinflammation on the rat hippocampal glycolytic pathway. *Journal of Neuroinflammation*, *19*(1), 255. 10.1186/s12974-022-02612-w 36221097 PMC9552490

[CIT0132] Wang, G., Huang, K., Tian, Q., Guo, Y., Liu, C., Li, Z., Yu, Z., Zhang, Z., & Li, M. (2024). S100A9 aggravates early brain injury after subarachnoid hemorrhage via inducing neuroinflammation and inflammasome activation. *iScience*, *27*(3), 109165. 10.1016/j.isci.2024.109165 38420589 PMC10901081

[CIT0133] Wang, L. Y., Tu, Y. F., Lin, Y. C., & Huang, C. C. (2016). CXCL5 signaling is a shared pathway of neuroinflammation and blood-brain barrier injury contributing to white matter injury in the immature brain. *Journal of Neuroinflammation*, *13*(1), 6. 10.1186/s12974-015-0474-6 26738635 PMC4704424

[CIT0134] Wang, Y., Ding, J., Song, H., Teng, Y., & Fang, X. (2022a). VSIG4 regulates macrophages polarization and alleviates inflammation through activating PI3K/AKT and inhibiting TLR4/NF-κB pathway in myocardial ischemia-reperfusion injury rats. *Physiology International*. 10.1556/2060.2022.00055 36057104

[CIT0135] Wang, Y., Jin, Z., Sun, J., Chen, X., Xie, P., Zhou, Y., & Wang, S. (2022b). The role of activated monocyte IFN/SIGLEC1 signalling in Graves’ disease. *The Journal of Endocrinology*, *255*(1), 1–9. 10.1530/JOE-21-0453 35695299

[CIT0136] Wei, C., Liu, J., Wu, B., Shen, T., Fan, J., Lin, Y., Li, K., Guo, Y., Shang, Y., Zhou, B., & Xie, H. (2025). Blockage of CCL3 with neutralizing antibody reduces neuroinflammation and reverses Alzheimer disease phenotypes. *Brain, Behavior, and Immunity*, *128*, 400–415. 10.1016/j.bbi.2025.04.034 40268067

[CIT0137] Xie, Y., Luo, X., He, H., & Tang, M. (2021). Novel insight into the role of immune dysregulation in amyotrophic lateral sclerosis based on bioinformatic analysis. *Frontiers in Neuroscience*, *15*, 657465. 10.3389/fnins.2021.657465 33994932 PMC8119763

[CIT0138] Yan, P., Zhu, A., Liao, F., Xiao, Q., Kraft, A., Gonzales, E., Perez, R., Greenberg, S. M., Holtzman, D., & Lee, J. M. (2015). Minocycline reduces spontaneous hemorrhage in mouse models of cerebral amyloid angiopathy. *Stroke*, *46*(6), 1633–1640. 10.1161/STROKEAHA.115.008582 25944329 PMC4442054

[CIT0139] Yao, L., Song, J., Meng, X. W., Ge, J. Y., Du, B. X., Yu, J., & Ji, F. H. (2020). Periostin aggravates NLRP3 inflammasome-mediated pyroptosis in myocardial ischemia-reperfusion injury. *Molecular and Cellular Probes*, *53*, 101596. 10.1016/j.mcp.2020.101596 32461137

[CIT0140] Yi, K., Cui, X., Liu, X., Wang, Y., Zhao, J., Yang, S., Xu, C., Yang, E., Xiao, M., Hong, B., Fang, C., Kang, C., Tan, Y., & Wang, Q. (2021). PTRF/Cavin-1 as a novel RNA-binding protein expedites the NF-κB/PD-L1 axis by stabilizing lncRNA NEAT1, contributing to tumorigenesis and immune evasion in glioblastoma. *Frontiers in Immunology*, *12*, 802795. 10.3389/fimmu.2021.802795 35069587 PMC8778801

[CIT0141] Zeng, X., Liu, G., Peng, W., He, J., Cai, C., Xiong, W., Chen, S., Yang, M., & Dong, Z. (2020). Combined deficiency of SLAMF8 and SLAMF9 prevents endotoxin-induced liver inflammation by downregulating TLR4 expression on macrophages. *Cellular & Molecular Immunology*, *17*(2), 153–162. 10.1038/s41423-018-0191-z 30552382 PMC7000402

[CIT0142] Zhang, D., Shen, X., Pang, K., Yang, Z., & Yu, A. (2021). VSIG4 alleviates intracerebral hemorrhage induced brain injury by suppressing TLR4-regulated inflammatory response. *Brain Research Bulletin*, *176*, 67–75. 10.1016/j.brainresbull.2021.08.008 34419512

[CIT0143] Zhang, Y., Chen, K., Sloan, S. A., Bennett, M. L., Scholze, A. R., O’Keeffe, S., Phatnani, H. P., Guarnieri, P., Caneda, C., Ruderisch, N., Deng, S., Liddelow, S. A., Zhang, C., Daneman, R., Maniatis, T., Barres, B. A., & Wu, J. Q. (2014). An RNA-sequencing transcriptome and splicing database of glia, neurons, and vascular cells of the cerebral cortex. *The Journal of Neuroscience: The Official Journal of the Society for Neuroscience*, *34*(36), 11929–11947. 10.1523/JNEUROSCI.1860-14.2014 25186741 PMC4152602

[CIT0144] Zhao, T., Zhang, X., Cui, X., Su, S., Li, L., Chen, Y., Wang, N., Sun, L., Zhao, J., Zhang, J., Han, X., & Cao, J. (2024). Inhibiting the IRAK4/NF-κB/NLRP3 signaling pathway can reduce pyroptosis in hippocampal neurons and seizure episodes in epilepsy. *Experimental Neurology*, *377*, 114794. 10.1016/j.expneurol.2024.114794 38685307

[CIT0146] Zhao, W., Beers, D. R., Henkel, J. S., Zhang, W., Urushitani, M., Julien, J. P., & Appel, S. H. (2010). Extracellular mutant SOD1 induces microglial-mediated motoneuron injury. *Glia*, *58*(2), 231–243. 10.1002/glia.20919 19672969 PMC2784168

[CIT0147] Zheng, M., Li, Z., Feng, Y., & Zhang, X. (2023). CD14 and CSF1R as developmental molecular targets for the induction of osteoarthritis. *International Journal of Clinical and Experimental Pathology*, *16*(8), 184–198. 37693684 PMC10492034

[CIT0148] Zheng, Y., Lee, S., Liang, X., Wei, S., Moon, H. G., & Jin, Y. (2013). Suppression of PTRF alleviates the polymicrobial sepsis induced by cecal ligation and puncture in mice. *The Journal of Infectious Diseases*, *208*(11), 1803–1812. 10.1093/infdis/jit364 23908488 PMC3814834

[CIT0149] Zhong, J., Wang, C., Zhang, D., Yao, X., Zhao, Q., Huang, X., Lin, F., Xue, C., Wang, Y., He, R., Li, X. Y., Li, Q., Wang, M., Zhao, S., Afridi, S. K., Zhou, W., Wang, Z., Xu, Y., & Xu, Z. (2024). PCDHA9 as a candidate gene for amyotrophic lateral sclerosis. *Nature Communications*, *15*(1), 2189. 10.1038/s41467-024-46333-5 PMC10928119 38467605

[CIT0150] Zhou, H. H., Zhang, Y. M., Zhang, S. P., Xu, Q. X., Tian, Y. Q., Li, P., Cao, D., & Zheng, Y. Q. (2021). Suppression of PTRF alleviates post-infectious irritable bowel syndrome via downregulation of the TLR4 pathway in rats. *Frontiers in Pharmacology*, *12*, 724410. 10.3389/fphar.2021.724410 34690766 PMC8529073

[CIT0151] Zhu, D., Chen, S., Sheng, P., Wang, Z., Li, Y., & Kang, X. (2024). POSTN promotes nucleus pulposus cell senescence and extracellular matrix metabolism via activing Wnt/β-catenin and NF-κB signal pathway in intervertebral disc degeneration. *Cellular Signalling*, *121*, 111277. 10.1016/j.cellsig.2024.111277 38944256

