# Supplementary material for: Dysregulated Expression of Inflammasome and Extracellular Matrix Genes in C9orf72-ALS/FTD Microglia
Source: ASN Neuro. 2025 Aug 7;17(1):2542998. doi: 10.1080/17590914.2025.2542998 (PMC12499538; doi:10.1080/17590914.2025.2542998)
Supplement: Revised Supplementary Figures and Information_anonymous.pdf [file TASN_A_2542998_SM0452.pdf]

Supplementary Figures and Information

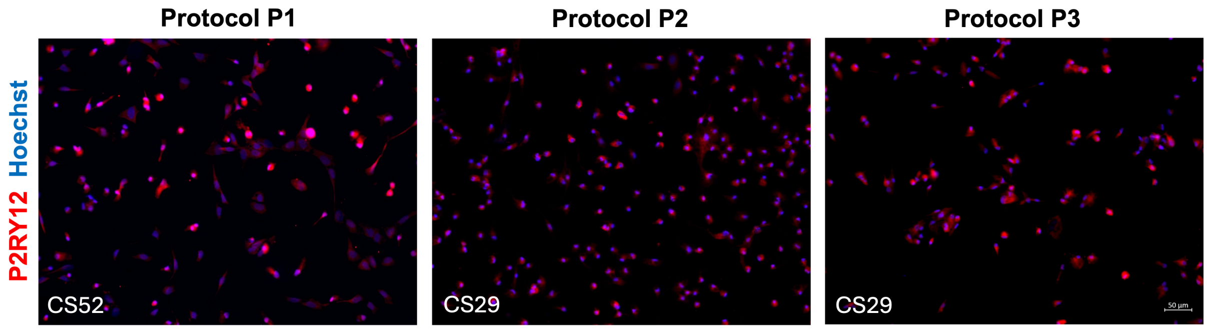

Figure S1. Expression of P2RY12 in iPSC-derived microglia generated using three differentiation protocols. Representative images of microglia preparations differentiated from iPSC lines CS52-C9n6-ISO (CS52) and CS29-C9n1-ISO (CS29) with protocols P1, P2, or P3 as indicated. Immunostaining was performed with an antibody against the typical microglia marker P2RY12 (red); nuclei were counterstained with Hoechst (blue). Scale bar = 50 µm.

DEGs	p value	log FC
NLRP3	1.557e-4	5.936
IL1-beta	5.829e-4	6.462
CASP1	1.295e-3	2.589
NLRP2	7.809e-5	8.911
NLRC4	6.479e-4	7.954
MEFV	1.607e-3	3.699
NINJ1	1.48e-3	1.345

Figure S2. Dysregulation of inflammasome genes in C9orf72-ALS microglia generated using derivation protocol P1. Shown are the p value and log Fold Change (logFC) for the indicated DEGs in CS52-C9n6-M versus CS52-C9n6-ISO microglia.

CS52-P1	CS52-P2
**Non-fibrillar collagens**	
COL6A6<br>COL17A1<br>COL23A1	COL4A1, COL4A2, COL4A4, COL4A5, COL4A6<br>COL9A3<br>COl12A1<br>COL25A1<br>COL26A1
**Laminin subunit beta**	
LAMB3	LAMB1<br>LAMB2
**Integrins**	
ITGA3<br>ITGA9<br>ITGB6	ITGA3<br>ITGA9<br>ITGB6

Figure S3. **Dysregulation of extracellular matrix genes in C9orf72-ALS microglia generated using derivation protocols P1 and P2.** Shown are genes encoding members of the non-fibrillar collagen, laminin subunit beta, and integrin families dysregulated in microglia generated using both differentiation protocols P1 and P2. CS52 = CS52-C9n6-M versus CS52-C9n6-ISO microglia; P1 and P2 = differentiation protocols P1 and P2.

Gene Symbol	logFC	PValue
**ASNSP1**	**6,5573082**	**6,98E-10**
**FPGT**	**6,89466988**	**6,52E-08**
**ZNF736**	**6,8771781**	**1,07E-07**
**ZNF717**	**5,67946815**	**1,49E-07**
**GNAS-AS1**	**4,58547975**	**1,80E-07**
**UTY**	**7,79357376**	**2,36E-07**
AC004846.1	4,07713295	2,74E-07
CCDC68	4,58288132	6,38E-07
PCDHA2	4,21377096	1,22E-06
ZNF558	6,30008912	1,29E-06
ZNF528-AS1	4,70548836	4,23E-06
CXCL5	4,19531914	1,64E-05
ZNF350	0,93081509	2,00E-05
SVIL-AS1	5,0471343	2,75E-05
ZNF880	1,67828107	3,15E-05

Figure S4. DEGs common to all RNAseq datasets. Shown are the 15 DEGs identified in all 25 RNAseq samples from C9orf72-ALS microglia and isogenic microglia. Log fold-changes (LogFC) and p values are shown for each gene.

Supplementary Information: DEG lists.

Protocol 1 CS52-C9n6-M microglia

614 Up-regulated DEGs in ALS vs Isogenic:

Gene Symbol	logFC	PValue	FDR
FYB1	8,28179947	5,19E-07	0,005000384
HLA-DPA1	8,089188426	1,15E-06	0,005000384
CSF1R	6,870667002	1,41E-06	0,005000384
NPL	5,192592985	2,12E-06	0,005000384
SAMHD1	3,781667	2,42E-06	0,005000384
CD74	8,026907097	2,46E-06	0,005000384
RASGRP3	5,748205259	2,76E-06	0,005000384
PTPRC	7,45189	3,10E-06	0,005000384
LILRB5	11,0091872	3,66E-06	0,005000384
SLCO2B1	9,274546141	3,86E-06	0,005000384
UTY	10,77818411	3,91E-06	0,005000384
CD53	6,639563681	4,48E-06	0,005000384
CD14	6,011664922	4,65E-06	0,005000384
MSR1	10,78788611	4,77E-06	0,005000384
APOBR	8,946527816	4,89E-06	0,005000384
TBXAS1	8,869678392	4,95E-06	0,005000384
PIK3CG	9,370598329	5,04E-06	0,005000384
MS4A4A	12,06986201	5,51E-06	0,005000384
PIK3R5	8,409013894	5,80E-06	0,005000384
CIITA	6,89251217	5,96E-06	0,005000384
SIGLEC1	9,791625448	6,53E-06	0,005225815
PIK3AP1	8,090244955	6,94E-06	0,005318912
HLA-DQA1	10,01407483	8,61E-06	0,006323369
FCGR2B	10,17835623	9,51E-06	0,006605306
ACP5	7,379086855	9,75E-06	0,006605306
OAS3	7,578985521	1,03E-05	0,006749312
TNFRSF14	8,901498621	1,21E-05	0,007344632
SLA	7,505761852	1,21E-05	0,007344632
CD163L1	8,359067217	1,28E-05	0,007344632
LMO2	8,172356326	1,31E-05	0,007344632
LPAR6	4,824934179	1,33E-05	0,007344632
ARHGAP18	2,660705176	1,40E-05	0,007449746
PTPRO	7,80610533	1,48E-05	0,007545392
PILRA	5,259641887	1,51E-05	0,007545392
KYNU	6,167594006	1,56E-05	0,007545392
GMFG	7,800113936	1,58E-05	0,007545392
AOAH	7,827759196	1,65E-05	0,007654449
MYO1F	5,078155294	1,73E-05	0,007793065
PARVG	9,07406037	1,81E-05	0,007985973
RCSD1	6,56462661	1,93E-05	0,008256027
FUCA1	2,89118817	2,01E-05	0,008256027
ZNF736	9,348720252	2,02E-05	0,008256027
TAL1	8,720669139	2,18E-05	0,008330235
LYZ	10,35819081	2,29E-05	0,008434832
PLCB2	4,831793842	2,39E-05	0,008434832
JAML	10,68528197	2,46E-05	0,008434832
LINC01094	4,192492623	2,47E-05	0,008434832
HLA-DRB1	8,322910737	2,59E-05	0,008434832
PLEK	9,71203892	2,61E-05	0,008434832
SRGN	9,017453525	2,71E-05	0,008434832
S100B	10,17531222	2,71E-05	0,008434832
RASGRP4	6,096955835	2,72E-05	0,008434832
SKAP2	3,163171441	2,75E-05	0,008434832
CHCHD2	10,42796639	2,91E-05	0,008434832
IL10RA	6,861598713	2,91E-05	0,008434832
HLA-DOA	9,416905662	2,97E-05	0,008434832
TMOD2	2,885803788	3,04E-05	0,008434832
COL17A1	3,34389366	3,05E-05	0,008434832
CTSC	4,638352423	3,11E-05	0,008434832
FCGR2A	8,551717162	3,14E-05	0,008434832
MYO7A	6,018582428	3,28E-05	0,008434832
OAS2	9,123508022	3,28E-05	0,008434832
LY86	9,170373942	3,29E-05	0,008434832
KCNJ5	5,652175609	3,32E-05	0,008434832
PECAM1	10,3652783	3,33E-05	0,008434832
DOCK8	4,177997049	3,64E-05	0,008434832
CLEC5A	9,00096887	3,65E-05	0,008434832
RNASE1	8,345177554	3,65E-05	0,008434832
HLA-DPB1	4,652817147	3,66E-05	0,008434832
MILR1	9,477823742	3,72E-05	0,008434832
DOCK10	7,696299217	3,77E-05	0,008434832
ATP8B4	6,445032908	3,78E-05	0,008434832

Gene Symbol	logFC	PValue	FDR
CD80	6,32815714	3,87E-05	0,008434832
AIF1	7,623601466	3,88E-05	0,008434832
SPI1	9,780471776	3,98E-05	0,008434832
CCDC170	8,021122535	4,00E-05	0,008434832
RNASE6	9,837996873	4,00E-05	0,008434832
CLEC7A	9,100772615	4,04E-05	0,008434832
ITGAX	5,949767878	4,06E-05	0,008434832
FERMT3	6,385349606	4,07E-05	0,008434832
CGAS	6,03191619	4,07E-05	0,008434832
TFEC	7,938088021	4,35E-05	0,008658171
GGTA1P	8,075866552	4,39E-05	0,008658171
THEMIS2	5,86667237	4,39E-05	0,008658171
FCGR2C	8,921660028	4,41E-05	0,008658171
ITGAM	10,5931861	4,42E-05	0,008658171
HLA-DMA	4,891386349	4,65E-05	0,008707687
C1QA	10,71679647	4,66E-05	0,008707687
FGD3	5,658499422	4,77E-05	0,008707687
RASSF4	3,016863762	4,77E-05	0,008707687
CPM	6,527812585	4,78E-05	0,008707687
CXCL12	2,626509255	4,93E-05	0,008707687
PLCG2	3,331226926	4,95E-05	0,008707687
TRPM2	9,38356109	5,04E-05	0,008707687
SIGLEC10	8,545617668	5,16E-05	0,008707687
SLC37A2	8,114307954	5,22E-05	0,008707687
WAS	5,761630326	5,25E-05	0,008707687
SIDT1	8,533534134	5,30E-05	0,008707687
GIMAP8	9,270345033	5,32E-05	0,008707687
C5AR1	5,529545609	5,33E-05	0,008707687
PTAFR	6,826027041	5,38E-05	0,008707687
CD86	8,723994821	5,40E-05	0,008707687
DHRS9	7,864891323	5,42E-05	0,008707687
HSPB6	6,898025403	5,42E-05	0,008707687
SIRPB2	8,987321461	5,44E-05	0,008707687
CLEC10A	8,549125354	5,60E-05	0,008881399
ARRB2	2,778908662	5,68E-05	0,008936703
ITGA9	4,958883859	5,84E-05	0,009031571
CXorf21	8,981860252	5,85E-05	0,009031571
TMEM273	9,360422582	5,90E-05	0,009031571

Gene Symbol	logFC	PValue	FDR
GIMAP1	6,339192385	6,06E-05	0,009122183
ARHGDIB	4,655443753	6,30E-05	0,009397818
TLR2	5,296650251	6,47E-05	0,009573235
RCBTB2	2,674069723	6,78E-05	0,009953274
MOB3B	2,722776013	6,85E-05	0,009977873
DOCK2	8,543842749	7,29E-05	0,010436692
CD300LF	9,904567777	7,45E-05	0,010586594
FGL1	9,1553943	7,57E-05	0,010662426
NLRP2	8,910850982	7,81E-05	0,010917326
ZNF558	8,950220717	8,05E-05	0,011167785
TRIM4	9,629527934	8,15E-05	0,011221711
BMPER	3,2736922	8,49E-05	0,011506919
WDFY4	8,91436294	8,49E-05	0,011506919
SYK	9,749356178	8,68E-05	0,011602379
DENND2D	3,963257951	8,69E-05	0,011602379
TREM2	8,634495081	9,10E-05	0,011797549
MFNG	8,085389015	9,12E-05	0,011797549
FMNL1	3,205825104	9,19E-05	0,011797549
CD28	9,392679979	9,22E-05	0,011797549
GIMAP6	8,760105833	9,45E-05	0,011797549
CRYBG1	4,957249092	9,48E-05	0,011797549
RASAL3	8,000667433	9,54E-05	0,011797549
LINC00996	9,505912711	9,54E-05	0,011797549
TIFAB	5,448882942	9,58E-05	0,011797549
MS4A6A	9,904787196	9,64E-05	0,011797549
MPP1	2,626389549	9,85E-05	0,011863536
MS4A7	10,01232048	9,86E-05	0,011863536
CD40	5,013709017	9,91E-05	0,011863536
LAPTM5	9,654134479	0,0001	0,011863536
TMC8	6,832634684	0,000101	0,011908497
CHIT1	10,63385845	0,000102	0,011952791
TM6SF1	5,504442233	0,000103	0,01196095
INPP5F	1,768997267	0,000104	0,01196095
TAGAP	5,600740436	0,000109	0,012472528
PRAM1	4,93662528	0,00011	0,012550151
TLR8	8,730496585	0,000112	0,012615334
ARHGAP4	3,369968727	0,000113	0,012634396
FCGR3A	8,812618723	0,000115	0,012634396

Gene Symbol	logFC	PValue	FDR
CYFIP1	1,694037028	0,000115	0,012634396
CCL13	10,24932224	0,000116	0,012634396
SIRPA	1,933032863	0,000117	0,012634396
B3GNT5	3,078538566	0,000117	0,012634396
STAC	5,04317839	0,000118	0,012634396
AMPD3	3,740674939	0,000118	0,012634396
IFI44	4,616516592	0,000118	0,012642595
FPR1	5,794923993	0,000119	0,012666762
HLA-DMB	10,36027649	0,000123	0,012962816
C1QB	9,689766957	0,000129	0,013529114
LPL	8,520985813	0,000131	0,013609257
ZNF280D	4,583266973	0,000132	0,013673393
SULT1C2	8,783045769	0,000133	0,013673424
BLNK	8,176803577	0,000134	0,013673424
CD48	5,567282459	0,000134	0,013673424
NCF4	13,3294147	0,000138	0,013976702
TYROBP	13,27639209	0,00014	0,014120632
AP1B1	1,682822112	0,000143	0,014265572
ALOX5AP	7,191156503	0,000146	0,014447488
SUCNR1	10,38796447	0,000146	0,014447488
ACE	6,086300126	0,000149	0,014617238
SLC2A9	5,466373257	0,000151	0,014807726
KLHL6	6,944626949	0,000153	0,014923297
FCMR	6,783949966	0,000154	0,014923297
NLRP3	5,93593668	0,000156	0,014989359
TRAF3IP3	5,976374334	0,000158	0,015059793
BTK	9,065545041	0,000158	0,015059793
TFCP2L1	5,983796717	0,000159	0,01507745
CBLN4	2,233535175	0,000162	0,015132561
CD180	9,744038447	0,000162	0,015132561
CORO1A	5,213528945	0,000167	0,01542859
ARHGAP30	9,798380234	0,000167	0,01542859
FCGR1A	8,257338221	0,000168	0,01542859
STAB1	9,560422492	0,000172	0,015600229
CSF2RB	7,822725767	0,000173	0,015600229
SLAMF7	4,690016627	0,000179	0,015993587
CD84	10,54366713	0,00018	0,015993587
TNFRSF1B	8,44871366	0,000182	0,016007646

Gene Symbol	logFC	PValue	FDR
DTX1	7,82503578	0,000184	0,01609315
MAP3K5	5,04976832	0,000186	0,0162157
MKNK1	1,552887228	0,000189	0,01636556
LIPA	3,042683965	0,000191	0,016385372
C1QC	10,0775624	0,000192	0,016385372
CCR1	9,198641557	0,000195	0,01651891
HLA-DRB6	7,910252198	0,000195	0,01651891
HPGDS	9,067895928	0,000201	0,016799149
ZNF502	7,064090561	0,000201	0,016799149
ADAP2	7,084399358	0,000202	0,016799149
LSP1	5,256491934	0,000205	0,01696959
TMEM86A	2,548351495	0,000211	0,017205783
OAS1	3,991163625	0,000212	0,017205783
SCARF1	4,225891719	0,000214	0,017205783
LST1	8,215229981	0,000215	0,017205783
ATP6V0D2	10,02047778	0,000216	0,017205783
MMP12	5,789640344	0,000216	0,017205783
NOD2	7,69421276	0,000217	0,017205783
SIGLEC12	6,268258205	0,000218	0,017205783
RHOH	6,898083685	0,000219	0,017235215
DOK3	3,617687444	0,000221	0,017263789
TMIGD3	7,417224491	0,000223	0,017344557
PSTPIP1	6,708027331	0,000224	0,017376926
ITGB2	7,033320373	0,000228	0,01743702
SPN	7,071880348	0,000228	0,01743702
ADGRE2	5,622014853	0,000229	0,01743702
LAIR1	9,378114576	0,000229	0,01743702
FGD2	9,264219988	0,000231	0,01743702
PTPN6	4,322481104	0,000232	0,01743702
CYTIP	8,761948269	0,000232	0,01743702
OTULINL	3,263539699	0,000233	0,01743702
ST14	5,628400241	0,000234	0,01744294
EVI2B	9,353730161	0,000235	0,01744294
IL16	7,911882988	0,000242	0,017875394
ST18	5,785160518	0,000244	0,017978082
NFAM1	6,039073771	0,000246	0,018007734
ADORA3	12,63376113	0,000248	0,018007734
CTTNBP2	2,689715402	0,00025	0,018007734

Gene Symbol	logFC	PValue	FDR
OSCAR	4,02462066	0,00025	0,018007734
TNFAIP2	4,513325333	0,00025	0,018007734
TLR1	4,564995462	0,000252	0,018039074
TNFRSF11A	6,250753056	0,000255	0,018146801
SLC15A3	4,323027165	0,000255	0,018146801
LINC01857	6,739803785	0,000263	0,018593607
CD4	6,083349117	0,000264	0,018623341
GAS2L3	2,266006784	0,000265	0,018623341
EYA2	5,779552067	0,000267	0,018685037
CYTH4	8,978370095	0,000268	0,018685037
CYBB	9,649697539	0,000274	0,018837069
TLR5	4,193777144	0,000277	0,018837069
IGSF6	9,903846599	0,000277	0,018837069
DOCK4	3,327290777	0,000278	0,018837069
ARHGAP45	3,961790183	0,000285	0,019070062
GATM	4,616544323	0,000287	0,019070062
DCX	4,454234951	0,000288	0,019070062
TEC	3,192173741	0,000288	0,019070062
CNN1	2,115412244	0,00029	0,019070062
MPEG1	9,531125889	0,000291	0,019070062
SLCO2A1	3,24197092	0,000294	0,019070062
SLAMF8	6,77532272	0,000294	0,019070062
TBX15	4,509078688	0,000294	0,019070062
GPR84	7,251427945	0,000295	0,019070062
TSPAN33	3,290184695	0,000296	0,019070062
HLA-DRB5	7,847805778	0,000297	0,01911702
EPB41L3	2,182653887	0,000299	0,019135791
SLC18B1	2,043653088	0,000305	0,019326905
FLVCR2	2,987489642	0,000305	0,019326905
SIRPB1	5,1966338	0,000307	0,019398059
ZNF528-AS1	5,14651419	0,000308	0,019398059
FGR	8,274256365	0,000311	0,019495572
PLD4	5,278483826	0,000314	0,019559787
POTEF	4,668467757	0,000314	0,019559787
BIN2	9,292706682	0,000316	0,019559787
CCL3	12,46131423	0,000318	0,019559787
ARHGAP9	4,224792321	0,00032	0,019559787
KCNQ1	3,538492069	0,000324	0,019559787

Gene Symbol	logFC	PValue	FDR
RYR1	5,59768894	0,000324	0,019559787
MAFB	2,585951604	0,000325	0,019559787
RASSF2	6,29249117	0,000329	0,01966272
FAR2P1	6,013935992	0,00033	0,01966272
SCIMP	8,182376297	0,000331	0,01966272
MYH14	4,10106592	0,000332	0,01966272
ACER3	1,639373482	0,000333	0,01966272
GPSM3	3,376846505	0,000338	0,01990475
FES	2,640940097	0,000339	0,01990475
HSPA7	7,244865118	0,000341	0,01990475
VAV1	7,915571956	0,000341	0,01990475
PKD2L1	5,847297275	0,000344	0,019943677
SIGLEC15	3,801769301	0,000345	0,019943677
CASP10	2,29196327	0,000345	0,019943677
ADAMDEC1	6,178779805	0,000353	0,020216962
PLB1	2,928195096	0,000364	0,020665921
IL4I1	6,167989162	0,000365	0,020692726
IKZF1	8,039557161	0,000368	0,020693033
ACVRL1	3,834865947	0,000372	0,020884504
NME8	6,733137822	0,000378	0,021024656
VSIG4	8,993690026	0,000382	0,021101571
ADCY7	3,205276732	0,000385	0,021219392
PCDHGA8	5,968562606	0,00039	0,021319449
VAV3	7,249814676	0,000393	0,021412981
SELPLG	4,293003335	0,000394	0,021412981
HLA-DRA	10,61652721	0,000396	0,021463998
MYO1G	6,272842353	0,000399	0,021547759
TSPYL5	4,234176058	0,000401	0,021547759
RNF144B	3,019787348	0,000403	0,021547759
DPEP2	7,155746908	0,000404	0,021547759
ABHD12	1,484589092	0,00041	0,021771709
HLA-DQB1	4,987172901	0,000414	0,021771709
FGD5	3,26951668	0,000416	0,021771709
CD163	10,28506396	0,000418	0,021771709
AP002954,1	7,39680862	0,00042	0,021771709
NRROS	7,163569705	0,000423	0,021771709
SNX10	2,669812958	0,000424	0,021771709
SMAP2	1,697575104	0,000424	0,021771709

Gene Symbol	logFC	PValue	FDR
CR1	7,222630055	0,000425	0,021771709
NCF2	7,955510121	0,000426	0,021771709
PTPN18	1,684236926	0,00044	0,022350666
IRF5	4,847731392	0,000453	0,022955896
GIMAP4	11,73500857	0,000457	0,023046331
ARL11	7,940378947	0,00046	0,023095287
GK	2,351151675	0,000461	0,023095287
HCK	9,101042843	0,000471	0,023420644
BRCA2	1,585284208	0,000479	0,023604516
TDO2	2,652952471	0,000479	0,023604516
RCN3	2,516986052	0,00048	0,023604516
SASH3	11,82952747	0,000484	0,023763561
SLC11A1	5,313873015	0,000489	0,023854745
CAT	1,425927173	0,000491	0,023912098
TXNIP	1,403266526	0,000493	0,023912231
SGCD	2,296965976	0,000499	0,0241069
CD33	7,818016483	0,00051	0,024508295
S100A9	9,315815416	0,000511	0,024508295
PKD1L3	5,340917972	0,000512	0,024524432
CAPG	2,273433839	0,000516	0,024620041
SERPINA1	6,397448567	0,000521	0,024772763
ITGAL	8,892820609	0,000522	0,024772763
IL10	8,076168277	0,000531	0,025148738
NCKAP1L	8,898820981	0,000534	0,025148738
FCER1G	7,956798268	0,000537	0,025220661
CTSZ	3,333906463	0,000544	0,025468217
PRDM1	5,424403465	0,000548	0,025521092
IRF8	7,84351786	0,000551	0,025594619
PARP15	4,698317963	0,000573	0,026507406
ZNF528	2,507424313	0,000574	0,026507406
GNAS-AS1	4,796489153	0,000575	0,026507406
STARD8	2,690658993	0,000578	0,026529041
RNF130	1,431748751	0,000578	0,026529041
IQGAP2	3,213873369	0,00058	0,026529041
IL1B	6,461617182	0,000583	0,026529041
CD22	6,243466982	0,000587	0,026529041
MFSD1	1,498006046	0,000588	0,026529041
PARP12	2,190058321	0,000588	0,026529041

Gene Symbol	logFC	PValue	FDR
GPR85	4,460638923	0,000594	0,026700342
GFRA2	4,631044581	0,000599	0,026803282
RTN1	6,983656564	0,000601	0,026803282
CARD6	2,663748461	0,000601	0,026803282
LCP2	7,871683679	0,000611	0,026974584
AP005131,6	5,378996985	0,000611	0,026974584
GPR34	9,094725003	0,000611	0,026974584
SENCR	6,311817881	0,000613	0,026974584
GSDMA	3,380454375	0,000616	0,02707757
CD209	7,316535818	0,000627	0,027470167
CARMN	2,281157928	0,000634	0,027701971
PCDHGB4	5,624344421	0,000638	0,027701971
LGMN	2,909173672	0,000638	0,027701971
MICB	3,444569885	0,000638	0,027701971
NLRC4	7,953992006	0,000648	0,027977688
PTPN22	6,573609087	0,000649	0,027977688
CTSS	7,950152634	0,00065	0,027977688
CTSF	6,787731357	0,000652	0,027981623
TMEM144	1,87258043	0,000655	0,027981623
MICAL1	1,607943806	0,000655	0,027981623
FAM49A	2,69129392	0,000656	0,027981623
CCDC88A	1,266003791	0,000661	0,028047221
P2RY6	4,121028215	0,000663	0,028047221
CD200R1	6,344320467	0,000664	0,028047221
PIK3CD	1,630650751	0,000665	0,028047221
MNDA	7,301052495	0,000684	0,028620306
ARHGAP25	8,177734733	0,000686	0,028620306
ITGB3	2,016565734	0,000692	0,028778554
ANPEP	4,320236405	0,000702	0,029105396
FOXP1	1,33326232	0,000704	0,029111542
SH3KBP1	1,142152721	0,000712	0,029115947
TNFSF13	3,80833684	0,000715	0,029115947
MACC1	3,705437469	0,000715	0,029115947
ZIM2-AS1	6,446351068	0,000716	0,029115947
SLC38A6	1,571734624	0,000716	0,029115947
PYHIN1	3,74887697	0,000718	0,029115947
CRYBB1	6,125290758	0,000719	0,029115947
PDE3B	2,471784393	0,000724	0,029252734

Gene Symbol	logFC	PValue	FDR
P2RY12	11,20003929	0,00073	0,02944458
ROR2	1,801884701	0,000734	0,029533031
GNA15	11,20582227	0,000736	0,029544945
GIMAP2	3,566542816	0,000744	0,029746787
TLR4	7,743998093	0,000745	0,029746787
AC090559,1	4,955803388	0,000758	0,029961567
ADGRE4P	7,535193875	0,000762	0,029961567
PPFIA2	3,87505965	0,000765	0,029961567
ZNF385A	1,727507952	0,000765	0,029961567
BMF	1,144763341	0,000765	0,029961567
CD36	8,613863425	0,000765	0,029961567
CALHM2	5,827592387	0,000771	0,030089824
ITGB2-AS1	3,31471062	0,000772	0,030089824
GPR65	7,07498088	0,000782	0,03037594
MAPK13	1,685501123	0,000783	0,03037594
C20orf197	6,319153512	0,000789	0,030399413
ZNF248	6,882695631	0,000795	0,030399413
CD37	5,963620035	0,000796	0,030399413
CX3CR1	4,011656248	0,000798	0,030399413
SIGLEC9	7,854706506	0,000799	0,030399413
IDH1	1,388227819	0,000799	0,030399413
HVCN1	3,320491611	0,000802	0,030407912
FOLR2	8,855861593	0,000803	0,030407912
ALDH1A1	5,995752369	0,000807	0,030439129
SLFN12L	7,622639984	0,000814	0,030652233
REPS2	2,996649514	0,000822	0,030698672
SH3TC1	2,315296628	0,000825	0,030698672
MEF2C	5,171800484	0,000825	0,030698672
TMEM236	5,185497938	0,000825	0,030698672
CD93	6,862137044	0,000826	0,030698672
ACSM5	5,738355326	0,000829	0,030742218
CCL4	11,13818141	0,000831	0,030748986
C2	3,661251787	0,000836	0,030817315
S100A8	12,27563458	0,000836	0,030817315
SYT6	3,036895567	0,000848	0,030897635
TRIM14	2,371722289	0,000849	0,030897635
AF127936,1	7,80123506	0,00085	0,030897635
TOX	3,580214252	0,000852	0,030897635

Gene Symbol	logFC	PValue	FDR
SLFN11	4,250513191	0,000857	0,031001335
FCER1A	5,268165762	0,000868	0,031282249
ZNF717	6,729215268	0,000881	0,031556634
SLC45A4	1,297998977	0,000885	0,031617722
AC110995,1	10,91236206	0,000887	0,031617722
GRIN3A	4,653538426	0,000894	0,031800881
ITGB6	5,189717088	0,000908	0,032232175
PLEKHO2	1,832933351	0,00091	0,032256168
TUSC1	10,8766491	0,000913	0,032269722
APBA1	1,645690098	0,000914	0,032269722
PDGFB	2,894617282	0,000925	0,032535272
KCNE4	2,402709671	0,000925	0,032535272
AL034397,3	11,13837074	0,000929	0,032574425
P2RY13	10,81369997	0,00093	0,032574425
MBOAT1	3,267033274	0,000935	0,032692582
SMS	1,185802154	0,000942	0,032819597
TUBGCP5	1,233130142	0,000943	0,032819597
MS4A14	10,77308225	0,000949	0,032919707
L1TD1	3,54614487	0,000949	0,032919707
CCDC144NL-AS1	3,045787206	0,000955	0,032946885
TNFAIP8L2	10,81252064	0,00096	0,032946885
PRRG4	3,369042319	0,000965	0,032946885
FCGR1B	6,255402197	0,000965	0,032946885
SAMSN1	7,116199012	0,00097	0,03306718
TMEM62	2,698466126	0,000973	0,03307506
IRF4	4,863465357	0,000976	0,033114261
MMP9	6,263178518	0,000978	0,033114261
FRK	2,819883521	0,000982	0,033114261
RYR3	3,10859733	0,000982	0,033114261
IL1RN	5,161705228	0,001007	0,033577597
AC079062,1	10,6302316	0,001008	0,033577597
TMEM150B	6,365429938	0,001017	0,033702427
S100Z	3,547966378	0,00102	0,033702427
RENBP	3,997370544	0,001026	0,033702427
HLA-DQA2	10,56207903	0,001028	0,033702427
GM2A	1,885507153	0,001028	0,033702427
HS3ST2	7,805316269	0,001028	0,033702427

Gene Symbol	logFC	PValue	FDR
LRRK2	4,054104604	0,001029	0,033702427
MS4A4E	3,458710932	0,001033	0,03373871
PCDH12	7,215214285	0,00104	0,03373871
HTATSF1P2	3,201813462	0,00104	0,03373871
TUBA4A	2,280434359	0,00105	0,033972011
LYL1	3,365617624	0,001052	0,033972011
NCEH1	4,733357903	0,001054	0,033972011
GPR82	10,58157366	0,001055	0,033972011
GABRA3	5,844797366	0,001069	0,034295774
TLR6	2,839620085	0,001073	0,034368114
FAM20A	2,978357025	0,001077	0,034424809
MAP2	1,933533242	0,00108	0,034454506
GPR183	5,968924216	0,001082	0,034454506
SDS	7,17473598	0,001084	0,034454506
SLC29A3	2,930344723	0,001086	0,034454506
HK3	10,73597853	0,001111	0,035091575
RGL1	1,297203489	0,001112	0,035091575
ADA2	4,965079929	0,001114	0,035091575
CNDP2	0,982476627	0,001119	0,035187874
C5AR2	10,57670405	0,001124	0,035307573
FPR3	7,657348483	0,001128	0,035336313
EMILIN2	2,758365841	0,001133	0,035336313
IFI44L	6,362072082	0,001133	0,035336313
ANKRD44	1,997364291	0,001144	0,035596115
XAF1	3,632228796	0,001147	0,035622768
C3AR1	7,04221699	0,001157	0,035852074
DES	4,70851437	0,001158	0,035852074
ZNF132	4,517214265	0,001165	0,035940247
SGO2	1,27951601	0,001167	0,035948546
TMEM140	4,111858604	0,001178	0,036184444
CAMK1	1,967127899	0,001192	0,036385878
LRRC4	2,046966121	0,001207	0,036770963
TRG-AS1	5,705760082	0,001209	0,036770963
LRRTM1	2,605742078	0,001211	0,036770963
FPGT	10,4622403	0,001227	0,037057877
EVI2A	7,303023424	0,001233	0,037057877
CARD9	3,318039353	0,001235	0,037057877
TAP2	1,65586481	0,001235	0,037057877

Gene Symbol	logFC	PValue	FDR
ERMN	3,432902487	0,001237	0,037074357
HAVCR2	7,774399707	0,001243	0,037104609
GPR141	10,40539867	0,001247	0,037162737
SNX20	10,60437411	0,001253	0,037243484
HMOX1	2,32642439	0,001254	0,037243484
CNTRL	1,392961857	0,001274	0,037598525
DOK2	10,41416224	0,001278	0,037610419
C18orf54	1,469536132	0,001279	0,037610419
TUBB1	5,731643415	0,001281	0,037610419
LGR5	2,599186509	0,001285	0,037661847
YWHAH	0,933236626	0,001292	0,037677331
HSD17B4	1,154175243	0,001294	0,037677331
CASP1	2,589465464	0,001296	0,037677331
GPR150	3,990460329	0,001298	0,037677331
GRHL2	4,35134866	0,001311	0,037925178
CEP128	1,967303064	0,001318	0,038014983
MYO5A	1,159350862	0,00132	0,038014983
TCTA	2,458638198	0,001323	0,038014983
PIK3R6	10,44490551	0,001325	0,038014983
DMXL2	1,328816762	0,001326	0,038014983
DENND1C	4,554282238	0,001332	0,038014983
ADGRE1	10,59808144	0,001332	0,038014983
CYSLTR1	4,251561	0,001333	0,038014983
VPS37B	1,517757685	0,001335	0,038014983
CD68	3,080635405	0,00134	0,038014983
MRC1	10,79013078	0,001343	0,038027627
LILRB2	10,18090198	0,001346	0,038046831
CCDC144NL	3,461425106	0,001358	0,038298237
CCR5	10,20579563	0,001359	0,038298237
PTPRJ	1,092327392	0,001363	0,038343581
CTSD	1,713011325	0,00137	0,038431613
ZNF710	1,431221358	0,001376	0,038491443
CLDN6	2,663680149	0,001379	0,038491443
CTSW	4,271464512	0,001379	0,038491443
ARMT1	1,202429077	0,001385	0,038550718
RAB39A	3,405482396	0,00139	0,038598252
LRRC3B	3,763521114	0,001393	0,038598252
TNFSF12	3,028491266	0,001394	0,038598252

Gene Symbol	logFC	PValue	FDR
UNC93B1	1,907785511	0,001398	0,038648217
ZNF366	4,131224499	0,001402	0,038711083
AC079612,1	2,700738699	0,001407	0,038783884
VSIR	1,641153622	0,001421	0,039060832
TLR7	7,846423616	0,001425	0,039092492
FABP5	2,147589059	0,001463	0,039908122
AC138207,4	2,451746188	0,001475	0,039998079
CCL4L2	7,882454543	0,001478	0,039998079
EPHX1	1,600347557	0,00148	0,039998079
NINJ1	1,344746727	0,00148	0,039998079
ST6GAL1	2,765314261	0,00152	0,041017182
LGMNP1	2,832336079	0,001534	0,04130682
LAP3	1,059740714	0,001545	0,041500613
HTR7	3,000654364	0,001557	0,041653643
PLA2G7	9,015182722	0,001569	0,041653643
RUNX3	6,044661884	0,001574	0,041693819
ZNF829	2,359843564	0,001591	0,041889655
ARRDC2	2,824201157	0,001605	0,041994956
MEFV	3,699126192	0,001607	0,041994956
GPR160	4,204371862	0,00161	0,041994956
SLC43A3	1,906568067	0,001612	0,041994956
CCL3L1	10,30089182	0,001631	0,042450705
LINC01088	2,903748765	0,001643	0,042699018
SLC28A1	5,452091392	0,001658	0,042917832
GRK3	2,150099072	0,001662	0,042917832
ANKRD34B	2,632688922	0,001666	0,042917832
LILRA6	9,956105304	0,00169	0,043440863
ASNSP1	5,893820322	0,001693	0,043440863
LTC4S	4,252356809	0,001694	0,043440863
TSPAN18	0,881473299	0,001707	0,043604706
AC116563,1	5,257704294	0,001711	0,043604706
TMEM26	2,515345413	0,001713	0,043604706
GPBAR1	3,03287435	0,001713	0,043604706
ZNF267	1,155644783	0,001716	0,043614851
ABI3	9,884529636	0,001727	0,043775392
ADGRE5	4,631347895	0,001731	0,043815848
ASPG	4,487545079	0,001743	0,043945985
KCNK13	9,753005099	0,001747	0,043945985

Gene Symbol	logFC	PValue	FDR
SOCS2-AS1	2,912195017	0,001749	0,043945985
AC009948,1	1,874390206	0,00175	0,043945985
GABRP	5,883696774	0,001751	0,043945985
CTNNA2	4,384754768	0,001768	0,044177814
ANKRD22	5,300910706	0,001777	0,044271318
APOL4	7,282256591	0,001781	0,044271318
FMN1	5,599296575	0,001788	0,044271318
FLOT1	1,152368652	0,001789	0,044271318
STAP1	4,70030232	0,001789	0,044271318
LILRB3	9,675878826	0,001795	0,044347741
PCED1B-AS1	9,728012445	0,001798	0,044347741
AL078590,3	6,720327526	0,0018	0,044347741
GALNT3	2,976368498	0,001812	0,044552982
TMEM200C	4,123304433	0,001815	0,044552982
LRMDA	4,484713907	0,001818	0,044552982
COL23A1	4,483580696	0,00184	0,044995195
LAMB3	2,563568206	0,00185	0,045132438
ATG4C	1,363699539	0,001884	0,045776188
RGS19	1,85047706	0,001911	0,046248396
MR1	1,896810893	0,001929	0,046377272
TDRD9	5,332259383	0,00193	0,046377272
GINS4	1,933907026	0,001947	0,046480764
ALPK2	1,067581374	0,001958	0,046480764
MNS1	9,5565706	0,00196	0,046480764
GRAP	3,82937061	0,00196	0,046480764
CENPE	1,263621279	0,001985	0,046863059
GUCY1A1	0,971312525	0,002005	0,047259956
AC097634,1	3,044015971	0,002007	0,047259956
SFMBT2	2,727962648	0,002017	0,047273586
RBL1	1,708695362	0,002017	0,047273586
HCLS1	5,719680621	0,002026	0,047273586
AL132655,1	4,754039801	0,002028	0,047273586
PPP1R16B	4,392830083	0,002029	0,047273586
TNFSF10	5,532386209	0,002043	0,04754697
ITGB7	2,785532256	0,002046	0,04755088
NRP1	1,96022921	0,002062	0,047843537
ADPRH	2,721037182	0,002069	0,04788685
TRIP13	1,490591803	0,002092	0,048201539

Gene Symbol	logFC	PValue	FDR
SH3D21	1,45879163	0,002098	0,048201539
ZNF501	9,355808916	0,002098	0,048201539
KNL1	1,751005164	0,0021	0,048201539
AL133371,2	9,35883424	0,002101	0,048201539
ZC3H12D	2,824678153	0,002122	0,048603122
LINC00504	3,250792907	0,002132	0,048701463
CCRL2	7,18325274	0,002137	0,048770649
PLCL2	2,638877643	0,002146	0,048893849
PRSS36	5,521657319	0,002176	0,049401728
TNNI2	4,006308489	0,002197	0,049746289
SCD5	1,337406101	0,002203	0,04982133

Protocol 1 CS52-C9n6-M microglia
165 Down-regulated DEGs in ALS vs Isogenic:

Gene Symbol	logFC	PValue	FDR
SLPI	-5,8690612	5,88E-06	0,00500038
PCSK6	-2,6021871	2,07E-05	0,0082903
FOXQ1	-5,216174	2,12E-05	0,0082903
DNAJC6	-2,5015069	3,11E-05	0,00843483
CLDN11	-3,6216467	4,73E-05	0,00870769
SCIN	-3,6853064	6,03E-05	0,00912218
NCR3LG1	-1,8511028	7,17E-05	0,01034592
RETREG1	-2,3789047	8,79E-05	0,01163832
SEMA3B	-2,978888	9,53E-05	0,01179755
SLC14A1	-4,7542153	0,0001	0,01186354
DDB2	-2,2140719	0,000162	0,01513256
ISYNA1	-1,7814959	0,000171	0,01556652
PEG10	-2,0741048	0,000174	0,01560023
PURPL	-4,6841289	0,000181	0,01599359
PIP5K1B	-2,3071125	0,00019	0,01638537
LINC01164	-4,6513734	0,000201	0,01679915
EFNA1	-2,8451967	0,000209	0,01720314
PCDHGB6	-1,9275035	0,000217	0,01720578
PHLDA3	-1,6612076	0,000248	0,01800773
GPC1	-1,3815186	0,000269	0,01868504
CCNG1	-1,6756171	0,000272	0,01879665
SSXP10	-4,1954614	0,000278	0,01883707
PTGES	-1,731315	0,000284	0,01907006
PLA2G4A	-1,660227	0,000292	0,01907006
FAS	-3,0047992	0,000302	0,01930552
LRRC1	-2,063611	0,000319	0,01955979
C1orf226	-2,2083282	0,000323	0,01955979
AK4	-1,5855874	0,000324	0,01955979
PKIA	-1,6379407	0,000325	0,01955979
RPRM	-3,0840138	0,000349	0,02007164
LNCTAM34A	-3,9791775	0,000351	0,0201641
MDM2	-1,8322391	0,000356	0,0202887
TP53	-2,5888618	0,000403	0,02154776
RAB27B	-2,4551812	0,000367	0,02069303
SLC6A20	-3,4788262	0,000374	0,02092719

Gene Symbol	logFC	PValue	FDR
FZD4	-1,2836584	0,000378	0,02102466
CD82	-1,7187713	0,000382	0,02110157
FAM160A1	-2,7972036	0,000388	0,02129054
LIF	-2,0951342	0,000406	0,02158489
PPM1H	-2,1789333	0,000416	0,02177171
UPK3B	-4,8485449	0,000423	0,02177171
PDGFD	-2,0414644	0,000423	0,02177171
USP53	-1,3479655	0,000426	0,02177171
ZMAT3	-2,9006654	0,00044	0,02235067
CYBRD1	-1,6186326	0,000461	0,02309529
DHCR7	-1,2128148	0,000464	0,02314456
ELOVL6	-1,5088472	0,000476	0,02360452
USP2	-2,0047275	0,000488	0,02385475
KCNIP1	-3,160396	0,000533	0,02514874
MIR34AHG	-2,5841716	0,000496	0,02401696
LINC00113	-6,6568238	0,000545	0,02547335
NAT8L	-2,5945359	0,000583	0,02652904
FDXR	-2,5835209	0,000589	0,02652904
PLP2	-1,26738	0,000605	0,02693214
BBC3	-1,8603384	0,000666	0,02804722
PPM1D	-1,2869713	0,000673	0,02827992
CTNNA3	-2,4562153	0,00068	0,02850351
NPW	-3,3615536	0,000693	0,02877855
VIPR2	-4,3108423	0,000711	0,02911595
SLC7A14	-2,8361318	0,000714	0,02911595
CD55	-1,7858233	0,000953	0,03294688
OPN3	-1,3856305	0,000747	0,029775
SHROOM2	-2,5283718	0,000749	0,029775
GPR143	-3,33397	0,000751	0,02979643
ANXA8	-3,8651629	0,000787	0,03039941
IL18	-3,7522621	0,000793	0,03039941
PTCHD4	-3,4760663	0,000804	0,03040791
SEMA3C	-1,2807768	0,000824	0,03069867
LRRN4	-3,8670682	0,000845	0,03089764
ADAM15	-1,2220691	0,000846	0,03089764
RPS27L	-1,6745215	0,000849	0,03089764
HLA-A	-1,6477974	0,000852	0,03089764
IER5L	-1,5493228	0,000867	0,03128225

Gene Symbol	logFC	PValue	FDR
AC124798.1	-1,8268152	0,00087	0,03128225
CXADR	-2,3832604	0,000877	0,03147821
LYNX1	-2,3141006	0,000788	0,03039941
PRKCI	-1,5349889	0,000956	0,03294688
BIRC3	-1,9237387	0,000962	0,03294688
CPNE7	-3,0992821	0,000964	0,03294688
MUC19	-5,4407323	0,000983	0,03311426
OGFRL1	-1,9177681	0,000985	0,03311426
SERPINB9	-1,42177	0,000998	0,03348983
SLC2A1	-1,5971852	0,001001	0,03353339
EDA2R	-4,3715354	0,001008	0,0335776
MTSS1L	-1,1279265	0,001018	0,03370243
FAM19A3	-2,5247693	0,001028	0,03370243
JUND	-1,1419014	0,001036	0,03373871
DAB1	-2,528126	0,001039	0,03373871
TP53INP1	-1,4662202	0,001067	0,03429577
YPEL2	-1,3377555	0,001098	0,03477345
WWC2	-1,4724703	0,001131	0,03533631
SLC16A7	-1,092197	0,001165	0,03594025
SLC45A2	-3,1907402	0,001179	0,03618444
BDKRB1	-2,2489569	0,001181	0,03619273
DTX4	-1,7651754	0,001183	0,03619273
ZNF219	-1,2645984	0,001214	0,03682189
GLIS2	-1,1937969	0,001231	0,03705788
LRP6	-0,9711096	0,001234	0,03705788
LRRC42	-0,9555911	0,00124	0,03707717
RAB11FIP5	-1,2738186	0,00126	0,0372952
NIPAL1	-1,7356302	0,00126	0,0372952
CMBL	-2,2243377	0,001268	0,03747032
GCNT2	-3,2169081	0,001294	0,03767733
CDKN1A	-4,1693228	0,001297	0,03767733
DUSP4	-1,7359743	0,001304	0,03777056
AC068700,1	-2,0922073	0,001339	0,03801498
PPARA	-0,9491585	0,001339	0,03801498
PARD6G	-1,2070359	0,001365	0,03834358
NOS1AP	-1,6910918	0,001381	0,03849144
ARMCX1	-1,0533802	0,001419	0,03904645
DOK4	-1,0161105	0,001454	0,039843

Gene Symbol	logFC	PValue	FDR
VAV2	-1,1395856	0,001461	0,03990812
PARD6B	-1,4334601	0,001463	0,03990812
FAM177A1	-0,9977007	0,001466	0,0399199
SEZ6L2	-0,9157779	0,00148	0,03999808
ITGA3	-2,6616239	0,001545	0,04150061
KAZN	-1,2257727	0,001551	0,04158488
BMP4	-1,6562286	0,001562	0,04165364
ADAMTS7	-1,6463991	0,001566	0,04165364
ANKRD18B	-2,1894018	0,001566	0,04165364
CAPN2	-1,1484579	0,00157	0,04165364
FASN	-0,9331989	0,00157	0,04165364
LGALS3BP	-1,234761	0,001579	0,04173854
SEMA3E	-2,9913286	0,001582	0,04173854
STX3	-1,0177371	0,001583	0,04173854
AL109976.1	-2,9931379	0,001604	0,04199496
AL157394.1	-3,075598	0,001611	0,04199496
PRKCB	-1,9060871	0,001611	0,04199496
PLEKHH2	-1,6722857	0,001651	0,04283229
KCNK15	-3,7850906	0,00166	0,04291783
WDR63	-2,144281	0,001666	0,04291783
CTSH	-1,6040411	0,001707	0,04360471
DIXDC1	-1,5858651	0,001726	0,04377539
KCNC4	-2,0010626	0,001743	0,04394598
TFAP2A	-2,858413	0,001765	0,04417781
FOXN3	-0,9950902	0,001767	0,04417781
MSANTD3	-1,0901172	0,00178	0,04427132
SPRY1	-2,0378117	0,001786	0,04427132
FLNC	-1,6574358	0,001818	0,04455298
BCL2L1	-1,3033397	0,001842	0,04499519
MYO10	-1,1161106	0,001858	0,04527539
NKX2-3	-3,661559	0,001866	0,04540433
GABRB2	-1,3472585	0,001892	0,04590334
SUSD5	-1,280602	0,00191	0,0462484
TMEM253	-2,4028764	0,001923	0,04637727
SCG5	-2,3688132	0,001924	0,04637727
UBXN4	-0,9579643	0,001927	0,04637727
COL6A6	-1,3476407	0,001938	0,04646901
PYGO1	-0,9034533	0,001939	0,04646901

Gene Symbol	logFC	PValue	FDR
NSG1	-1,6813673	0,001949	0,04648076
HLA-H	-1,5350241	0,001949	0,04648076
HEG1	-1,1059422	0,001952	0,04648076
EPHA2	-2,4640319	0,001959	0,04648076
ALDH1L1	-3,6794031	0,001973	0,04670825
SYTL5	-2,0229963	0,001977	0,04675358
PDP2	-1,1487957	0,002009	0,04725996
PDPN	-1,4027042	0,002026	0,04727359
WNT9A	-2,6799966	0,002027	0,04727359
DBP	-1,8178775	0,002064	0,04784354
KIAA1522	-1,4430363	0,002083	0,04815313
MMP15	-2,1493372	0,002099	0,04820154
AASS	-1,3159357	0,002126	0,04863272
PHLPP2	-0,9833219	0,002148	0,04889385
SPRR2F	-5,5540771	0,002163	0,04916504
NYNRIN	-1,2410707	0,002179	0,04940349

Protocol 1 CS29-C9n1-M microglia
53 Up-regulated DEGs in ALS vs Isogenic:

Gene Symbol	logFC	PValue	FDR
ARMCX6	3,73554071	2,12E-09	2,09E-05
ZNF677	10,2766946	3,15E-09	2,09E-05
ZNF667	9,33509832	3,69E-09	2,09E-05
ARMCX2	2,32511841	6,39E-09	2,47E-05
ZNF829	7,85736454	8,23E-09	2,47E-05
PUS7L	8,59660574	8,74E-09	2,47E-05
INPP5F	2,02762133	1,06E-08	2,56E-05
UTY	8,85314819	1,32E-08	2,80E-05
AC016747,1	7,30267625	1,78E-08	3,36E-05
ZNF595	7,65821819	4,31E-08	6,95E-05
ZNF229	8,79676897	4,51E-08	6,95E-05
IRAK4	8,25854142	6,47E-08	9,14E-05
SVIL-AS1	5,93133391	7,86E-08	0,00010244
CEBPZOS	6,23315721	1,07E-07	0,00012804
EDNRB	3,43823106	1,13E-07	0,00012804
FPGT	7,71767834	1,25E-07	0,0001321
ACOT2	4,59197823	1,62E-07	0,00016177
ZNF667-AS1	11,2431612	2,74E-07	0,00025836
ZNF471	11,8811204	5,83E-07	0,00051982
ZNF568	2,88603398	1,17E-06	0,00098919
ZNF528	3,30209582	2,71E-06	0,00218505
ZNF736	6,42770385	5,25E-06	0,00396629
S100B	1,43504389	5,38E-06	0,00396629
IL7R	1,70299429	7,44E-06	0,00525754
ZNF717	8,95335909	8,26E-06	0,0055986
ACOT1	5,53958939	9,44E-06	0,00615209
GNA15	1,15027139	1,70E-05	0,00995616
ZNF300	2,3107979	1,87E-05	0,01035857
ZNF880	2,52267651	1,89E-05	0,01035857
FNBP1P1	4,48210312	2,36E-05	0,01144262
CCDC68	5,13888276	2,54E-05	0,01190875
EHHADH	2,64318148	2,60E-05	0,01190875
ZNF790-AS1	8,10739599	2,70E-05	0,01202874
HAS1	2,70015482	3,48E-05	0,01511654
S100A9	1,49288773	5,71E-05	0,02185176
MAP3K13	3,68404423	5,82E-05	0,02185176
ASNSP1	9,0220488	5,90E-05	0,02185176
GNAS-AS1	4,19572779	6,76E-05	0,02338464
MAP3K8	0,94244272	7,40E-05	0,02508529
CXCL5	4,1886755	7,73E-05	0,02515331
HLA-DQA2	2,93217879	7,87E-05	0,02515331
SLAMF8	1,43287238	8,14E-05	0,02554722
ARHGAP22	1,59540547	8,68E-05	0,02567491
MNS1	2,24159349	8,90E-05	0,02567491
SPINK6	2,33587057	8,93E-05	0,02567491
CD14	1,21486709	9,69E-05	0,02691427
ZNF350	1,04603569	0,00011118	0,02991252
VSIG4	1,23178888	0,00011716	0,03102925
PCDHA2	4,52930948	0,0001272	0,03223946
ZNF311	3,1864568	0,00016366	0,03834396
TRIM4	1,12489349	0,0001739	0,0398323
RDH10	0,60896556	0,00018158	0,03997152
CRIM1	1,02284527	0,00020186	0,04277336

Protocol 1 CS29-C9n1-M microglia
28 Down-regulated DEGs in ALS vs Isogenic:

Gene Symbol	logFC	PValue	FDR
KCNC3	-3,1035518	1,35E-05	0,00824914
CYP26B1	-3,6487665	1,36E-05	0,00824914
SLC4A4	-1,9306216	2,07E-05	0,01093811
CDH7	-1,1333779	2,13E-05	0,01093811
KIAA1024	-1,2321403	2,21E-05	0,0110014
IGDCC3	-1,6099504	5,37E-05	0,02185176
PLPPR4	-1,3782634	5,68E-05	0,02185176
HAS2	-1,5973863	5,76E-05	0,02185176
ADGRL3	-0,8278532	5,93E-05	0,02185176
TCIM	-2,2365363	6,12E-05	0,02208489
TMEM100	-2,1454053	6,30E-05	0,02226429
PTCHD1	-2,8799393	7,58E-05	0,02515331
MYH14	-6,0890449	8,52E-05	0,02567491
SLC1A6	-5,56486	8,94E-05	0,02567491
ARHGAP40	-5,5153471	9,11E-05	0,0257218
GPM6B	-1,2958553	0,00011044	0,02991252
SLN	-3,9122932	0,00012555	0,03223946
PCDHB17P	-2,010803	0,00012825	0,03223946
SLITRK6	-2,9332349	0,00012934	0,03223946
SOX8	-1,4985249	0,00013695	0,03364131
PCDH9	-2,2589927	0,00013919	0,03370493
FBXL21	-4,6644319	0,00014445	0,03448418
EPCAM	-3,8542727	0,00016514	0,03834396
TMEM30B	-5,2833933	0,00017671	0,03993718
ITGB6	-3,4076359	0,00018023	0,03997152
PLAGL1	-0,8682668	0,0001904	0,04137638
AC110619,1	-3,6098137	0,00020188	0,04277336
LEFTY2	-2,3588747	0,00022922	0,04796637

Protocol 2 CS52-C9n6-M microglia
890 Up-regulated DEGs in ALS vs Isogenic:

Gene Symbol	logFC	PValue	FDR
POSTN	5,62793007	1,17E-08	0,00019159
MGP	5,66165737	9,62E-08	0,00044193
AKAP12	3,70117551	1,15E-07	0,00044193
TNC	5,85305143	1,18E-07	0,00044193
PXDN	3,61663147	1,61E-07	0,00044193
PLS3	3,63971961	2,09E-07	0,00044193
KRT8	3,41004873	2,15E-07	0,00044193
FBN1	3,4343672	2,17E-07	0,00044193
NRK	4,88489934	2,47E-07	0,00044699
FRAS1	5,21024956	2,85E-07	0,00046446
CDH11	3,24080683	3,13E-07	0,00046446
IGFBP5	3,14680551	3,45E-07	0,00046649
FN1	2,96683666	3,72E-07	0,00046649
MAP1B	3,54382884	4,83E-07	0,00056283
KRT18	3,13916669	6,24E-07	0,00061867
LAMB1	3,28792984	6,31E-07	0,00061867
FLNC	3,7169792	6,70E-07	0,00061867
BGN	4,36354559	7,18E-07	0,00061867
CDH2	2,92652211	7,20E-07	0,00061867
CYR61	3,79822541	7,93E-07	0,00064722
SERPINE2	2,77635004	1,00E-06	0,00076031
VCAN	3,05633757	1,03E-06	0,00076031
CAVIN1	2,75572745	1,10E-06	0,00078355
ITGA11	3,51837112	1,21E-06	0,0008239
FBLN5	4,99946417	1,41E-06	0,00091137
CCDC80	2,66486069	1,45E-06	0,00091137
LIMCH1	4,43213246	2,05E-06	0,00113287
PODXL	5,15326697	2,09E-06	0,00113287
SEMA3D	5,99779922	2,09E-06	0,00113287
PARVA	3,46667102	2,20E-06	0,00113287
PAPPA	2,86595721	2,21E-06	0,00113287
CCND2	2,24201269	2,22E-06	0,00113287
COL4A5	4,33605356	2,46E-06	0,00121712
LOXL2	3,63114051	2,60E-06	0,0012461
ITGA1	3,01353661	2,75E-06	0,00124992

Gene Symbol	logFC	PValue	FDR
CRABP2	4,39520247	2,76E-06	0,00124992
SCUBE3	4,69958292	2,94E-06	0,00127894
DSG2	3,19845832	2,98E-06	0,00127894
ZFHX4	3,90826717	3,23E-06	0,0013298
NOTCH3	4,09300425	3,40E-06	0,0013298
ENC1	2,49768659	3,44E-06	0,0013298
MFAP2	4,46216857	3,57E-06	0,0013298
COL18A1	2,54699592	3,59E-06	0,0013298
SYNPO	2,7943679	3,60E-06	0,0013298
FAT1	2,87447432	3,67E-06	0,0013298
LTBP1	4,12658391	3,81E-06	0,00135242
HMCN1	3,18519003	4,22E-06	0,00146347
TGM2	2,35639531	4,70E-06	0,00157008
HEPH	4,6785346	4,80E-06	0,00157008
KRT7	4,64588417	4,85E-06	0,00157008
FRMD6	2,93993621	5,05E-06	0,00157008
NFIB	4,39567704	5,08E-06	0,00157008
CDKN2B	2,23329846	5,21E-06	0,00157008
CTTN	2,73211402	5,27E-06	0,00157008
COL25A1	4,15952664	5,29E-06	0,00157008
IGFBP3	2,47824717	5,45E-06	0,00158901
SVIL	3,49887473	5,92E-06	0,00169388
KIRREL1	3,78878046	6,12E-06	0,00172064
MYLK	4,05322474	6,32E-06	0,001747
OLFML1	6,88618359	6,99E-06	0,00190095
PTPRF	3,31299516	7,32E-06	0,0019577
EDIL3	3,43387818	7,88E-06	0,00207276
TPM1	2,90591911	8,02E-06	0,00207647
PTGIS	4,60209523	8,18E-06	0,0020845
MOXD1	5,6027083	8,52E-06	0,00211356
RNF150	4,58165755	8,55E-06	0,00211356
NAV2	3,43102846	8,91E-06	0,00216932
KRT19	5,18229741	9,33E-06	0,00223233
NFIX	5,2309138	9,70E-06	0,00223233
FKBP10	2,89950425	9,82E-06	0,00223233
FSTL1	3,2934297	9,97E-06	0,00223233
CALD1	3,02445463	1,02E-05	0,00223233
WWTR1	3,29045467	1,02E-05	0,00223233

Gene Symbol	logFC	PValue	FDR
SFRP1	5,92331062	1,04E-05	0,00223233
MSRB3	3,92803217	1,05E-05	0,00223233
CLSTN2	2,69739244	1,05E-05	0,00223233
FHL1	3,28135543	1,30E-05	0,00262458
ST6GAL2	4,86367419	1,31E-05	0,00262458
CPXM1	5,41845693	1,31E-05	0,00262458
PDE5A	4,49066515	1,32E-05	0,00262458
ANXA3	3,2818645	1,33E-05	0,00262458
TEAD2	4,4183559	1,34E-05	0,00262458
LAMA4	7,02047731	1,35E-05	0,00262458
GFRA1	3,4497071	1,40E-05	0,0026599
PAWR	3,59825308	1,40E-05	0,0026599
TINAGL1	3,63039111	1,51E-05	0,00277508
THSD4	5,41814085	1,52E-05	0,00277508
IGFBP2	3,9418353	1,52E-05	0,00277508
AMOTL2	3,32360111	1,53E-05	0,00277508
EFNB2	3,01259488	1,55E-05	0,00278219
EXT1	2,30072962	1,59E-05	0,00282184
CAPN2	2,63308512	1,69E-05	0,00292582
AFAP1	2,44439944	1,71E-05	0,00293317
MID1	3,71304831	1,89E-05	0,00320787
CPE	3,75337306	1,93E-05	0,00323879
CAV1	3,36300727	1,96E-05	0,00323882
LAMA5	2,73574518	2,01E-05	0,00323882
GATA6	3,56233095	2,01E-05	0,00323882
EPHB4	3,8697655	2,03E-05	0,00323882
FAM171A1	3,58664567	2,03E-05	0,00323882
MEX3A	3,72254155	2,10E-05	0,00329035
TTN	3,90766696	2,12E-05	0,00329671
DSP	2,84553933	2,25E-05	0,00346323
SFRP4	8,13284458	2,28E-05	0,0034798
NYNRIN	3,31824311	2,31E-05	0,00349474
MCAM	3,6034729	2,45E-05	0,00361328
GDF6	3,00801027	2,50E-05	0,00361328
SYTL5	4,66974519	2,50E-05	0,00361328
EHD2	3,21031704	2,51E-05	0,00361328
SLC16A9	5,47463891	2,51E-05	0,00361328
FAP	3,81305994	2,52E-05	0,00361328

Gene Symbol	logFC	PValue	FDR
MYL9	2,80347118	2,56E-05	0,00363809
DMD	3,02603293	2,62E-05	0,00367863
PFN2	2,46136738	2,66E-05	0,00367863
NCKAP1	3,15604027	2,66E-05	0,00367863
MET	4,52907568	2,81E-05	0,00385287
SALL2	5,61260783	2,88E-05	0,00391256
ENAH	2,64134979	2,98E-05	0,00398885
TSPAN2	9,94494649	3,01E-05	0,00398885
DCHS1	3,58823441	3,01E-05	0,00398885
SPARC	3,15014887	3,17E-05	0,00407349
SHROOM2	4,95651082	3,18E-05	0,00407349
PCSK9	6,45610181	3,20E-05	0,00407349
JAG1	1,82057087	3,21E-05	0,00407349
AASS	3,62077896	3,24E-05	0,00407349
MAP1A	3,19199032	3,26E-05	0,00407349
NLGN4X	5,9551986	3,29E-05	0,00407349
NNMT	2,79091227	3,31E-05	0,00407349
MYOCD	3,9990452	3,31E-05	0,00407349
YAP1	3,80247196	3,32E-05	0,00407349
DCLK1	5,93221053	3,36E-05	0,00407551
LRRC17	4,1471346	3,37E-05	0,00407551
GJC1	3,55713143	3,41E-05	0,00409246
DSC3	2,63039653	3,46E-05	0,00410988
PRKG1	5,28395229	3,48E-05	0,00410988
BMP1	2,21171684	3,51E-05	0,00412553
DLC1	3,13847892	3,55E-05	0,00413476
SPOCK1	2,96839388	3,73E-05	0,00426087
ERBB3	5,18430307	3,73E-05	0,00426087
PEG10	1,93143919	3,81E-05	0,00431732
NOX4	4,5152473	3,92E-05	0,00439273
DAB2IP	3,50914637	3,93E-05	0,00439273
DOCK9	3,29956891	3,99E-05	0,0044347
CHST3	3,96031875	4,14E-05	0,00452889
ANO4	4,60209898	4,17E-05	0,00452889
TFPI	3,09513351	4,18E-05	0,00452889
FAT4	3,7628206	4,22E-05	0,00452889
ERRFI1	2,08275435	4,23E-05	0,00452889
IL33	6,96075307	4,25E-05	0,00452889

Gene Symbol	logFC	PValue	FDR
KIF5C	5,85631358	4,44E-05	0,00468254
CDC42EP1	3,5462462	4,50E-05	0,00468254
MIR100HG	3,48058877	4,51E-05	0,00468254
B3GALT2	5,03130787	4,53E-05	0,00468254
CNTN1	4,73240123	4,56E-05	0,00468254
TEAD3	3,53103032	4,57E-05	0,00468254
ITGA3	2,42789859	4,59E-05	0,00468254
CAP2	3,25369202	4,70E-05	0,00476579
OLFM4	6,43585522	4,83E-05	0,00486743
VANGL2	4,06748489	4,90E-05	0,00490026
COL26A1	5,01914031	4,98E-05	0,00490026
INHBA	2,53445359	4,98E-05	0,00490026
MAP3K20	1,64056119	5,09E-05	0,00491114
PLCE1	4,33546541	5,13E-05	0,00491114
GABRB3	4,44507841	5,16E-05	0,00491114
TYRO3	4,4023041	5,17E-05	0,00491114
SGCD	3,89345612	5,17E-05	0,00491114
ADGRL3	3,62842366	5,18E-05	0,00491114
ARSJ	4,25647365	5,21E-05	0,0049115
AJUBA	3,15581908	5,24E-05	0,0049115
CACNA1H	6,15214165	5,43E-05	0,00503901
PTPN14	2,91386136	5,43E-05	0,00503901
LAYN	4,15102379	5,52E-05	0,00507911
SMOC2	4,53667813	5,57E-05	0,00507911
ERG	4,80685284	5,57E-05	0,00507911
GBP1	4,04569061	5,74E-05	0,00517761
TNS1	2,23400627	5,74E-05	0,00517761
PKP2	2,81294666	5,93E-05	0,00530985
RBPMS	3,37207724	5,99E-05	0,00530985
SYT10	9,73894626	5,99E-05	0,00530985
LMCD1	2,69872382	6,10E-05	0,00538302
ADGRL2	4,03882148	6,23E-05	0,00544237
LIMS2	5,24769785	6,24E-05	0,00544237
PTPRD	4,4020317	6,35E-05	0,00550752
LINC00632	3,70854939	6,38E-05	0,00550752
TFPI2	2,73162801	6,59E-05	0,00563175
ANK3	3,35837382	6,59E-05	0,00563175
SLC37A3	2,48913051	6,64E-05	0,00564254

Gene Symbol	logFC	PValue	FDR
MDFI	4,37533487	6,70E-05	0,00566787
SAMD5	4,90380766	6,82E-05	0,00570514
NPR3	5,8512363	6,83E-05	0,00570514
CCDC3	5,05646363	6,85E-05	0,00570514
PRKD1	3,85675903	7,13E-05	0,0059073
TSPAN9	3,34342839	7,20E-05	0,00591124
CD248	5,84522962	7,22E-05	0,00591124
GPC6	3,52109136	7,26E-05	0,00591124
ITGA2	2,56361972	7,28E-05	0,00591124
NEBL	4,48325147	7,57E-05	0,00611701
CAV2	3,15468615	7,61E-05	0,00611746
ZNF521	4,19686916	7,69E-05	0,00615454
CILP2	5,98552738	7,76E-05	0,00615911
GAS2	3,94542374	7,80E-05	0,00615911
BCAR1	2,98032293	7,83E-05	0,00615911
KRT17	5,0990418	7,86E-05	0,00615911
SLITRK5	5,40324369	7,89E-05	0,00615911
NHSL2	4,63026759	7,98E-05	0,00620207
HDGFL3	2,17245352	8,06E-05	0,00621427
ASPN	7,16424037	8,07E-05	0,00621427
RARB	4,63099664	8,14E-05	0,00622757
AEBP1	4,2761636	8,19E-05	0,00622757
PCDH7	3,88325569	8,21E-05	0,00622757
TUSC3	3,36535715	8,44E-05	0,0063123
ZFPM2	4,04917023	8,45E-05	0,0063123
COL2A1	5,66675148	8,50E-05	0,0063123
SYNPO2	3,18846131	8,51E-05	0,0063123
NR2F2	3,3547018	8,51E-05	0,0063123
F2R	1,92750756	8,59E-05	0,00634357
RAB34	2,80051267	8,71E-05	0,00636972
LTBP2	2,09087613	8,73E-05	0,00636972
ARHGAP23	3,75387475	8,74E-05	0,00636972
SERPINH1	1,536873	8,81E-05	0,00639223
C1orf198	1,39536574	8,93E-05	0,00645034
PHLDB2	3,37475802	9,10E-05	0,00651593
PALM	3,91402613	9,26E-05	0,00660196
VCL	1,77603373	9,35E-05	0,00663124
NTRK2	5,9085843	9,51E-05	0,00670474

Gene Symbol	logFC	PValue	FDR
EPHA4	3,9190569	9,53E-05	0,00670474
TET1	3,81153627	9,57E-05	0,00670474
JAM3	3,57570671	9,63E-05	0,00671863
CACNA2D1	3,18155535	9,68E-05	0,00671926
S100A10	1,66280611	9,77E-05	0,00675755
TEAD1	1,72839882	9,94E-05	0,00682425
FILIP1	3,51366759	0,00010035	0,00682425
LAMC2	3,44029893	0,00010036	0,00682425
PATJ	4,21706564	0,00010037	0,00682425
DACT1	3,6831958	0,0001011	0,00684558
SERPING1	3,31217958	0,00010209	0,0068838
MEIS3	4,02677769	0,00010419	0,00699693
PLN	4,16354486	0,00010724	0,00703509
EHD3	3,66123991	0,00010725	0,00703509
BAMBI	2,69541873	0,00010782	0,00703509
LEFTY2	8,95691347	0,00010789	0,00703509
ROBO2	4,17661665	0,0001085	0,00703509
IL17RD	3,94019526	0,00010884	0,00703509
MXRA8	3,03002161	0,00010927	0,00703509
LYPD6	6,38797276	0,00010969	0,00703509
CDH3	4,02370351	0,00010974	0,00703509
PSAT1	1,52058114	0,00010991	0,00703509
GREB1L	6,38691979	0,00011026	0,00703509
ADAMTS7	4,55047346	0,00011038	0,00703509
KCTD15	3,43379802	0,00011057	0,00703509
ISLR	4,83137158	0,0001108	0,00703509
VASH2	6,58082163	0,00011206	0,00708044
PDE3A	3,42683071	0,00011238	0,00708044
FIGN	4,84601335	0,00011332	0,00711194
SHROOM3	3,42865794	0,0001144	0,00712591
GLI2	3,95159044	0,00011441	0,00712591
ADAMTS16	4,41710598	0,00011808	0,00728666
C7	6,25295571	0,00011815	0,00728666
SH3BP4	2,33766593	0,00011833	0,00728666
PPP2R2B	5,39360242	0,00012108	0,0073581
MYH11	4,90442643	0,00012144	0,0073581
SCARA3	4,30931219	0,00012145	0,0073581
SERTAD4	3,82352428	0,00012177	0,0073581

Gene Symbol	logFC	PValue	FDR
JUP	1,43505791	0,00012217	0,0073581
OSMR	3,08355888	0,00012266	0,0073581
TSPAN18	4,09326494	0,0001227	0,0073581
ANXA1	1,31960304	0,0001231	0,0073581
NEXN	2,87434343	0,00012462	0,00736317
PXYLP1	4,35536814	0,00012463	0,00736317
SEMA6A	3,88108679	0,0001253	0,00736317
GPX8	3,87618997	0,0001256	0,00736317
TSHZ2	6,6603899	0,0001269	0,00736317
CNTNAP2	5,19448606	0,00012695	0,00736317
KLF12	3,65841958	0,00012749	0,00736317
CACNG4	6,32660629	0,00012755	0,00736317
GABBR1	2,90426216	0,00012778	0,00736317
SORBS1	3,09518933	0,00012805	0,00736317
TPM2	2,29868776	0,00012827	0,00736317
VLDLR	3,14014712	0,0001286	0,00736317
HOXA3	8,83362734	0,00012924	0,00737395
MDK	3,53623096	0,00013282	0,00751823
PDGFRB	3,60891418	0,00013357	0,00751823
MPPED2	4,58085199	0,00013401	0,00751823
TMEM98	3,4992593	0,00013428	0,00751823
CDH6	3,59271063	0,00013453	0,00751823
LRCH2	3,68674369	0,00013513	0,00752554
FNDC5	4,95329104	0,00013587	0,00754119
COL12A1	4,82122791	0,00013953	0,00770283
ZDHHC8P1	6,15945667	0,00013985	0,00770283
AMOTL1	3,3203486	0,0001402	0,00770283
ITGB6	4,62374724	0,0001413	0,00771669
CSRP2	2,63390525	0,0001414	0,00771669
UNC5C	8,78511295	0,00014249	0,00775075
COL9A3	3,13759441	0,00014346	0,00776913
BMP4	3,29080314	0,00014378	0,00776913
NCALD	3,89864996	0,00014652	0,00787431
EFEMP2	2,05440158	0,0001467	0,00787431
CLIP3	2,76558965	0,00015171	0,00809257
SNAI2	3,48899951	0,00015206	0,00809257
LOXL1	3,23409142	0,00015225	0,00809257
FBN3	8,73294865	0,00015299	0,00810573

Gene Symbol	logFC	PValue	FDR
CNTN4	3,76915262	0,00015495	0,00813153
SLITRK6	4,96153735	0,00015509	0,00813153
TDRP	3,74066405	0,00015547	0,00813153
SEMA5A	2,31298481	0,00015729	0,00820017
FLT1	1,67264145	0,00015841	0,00823233
ID4	4,02179427	0,00016002	0,00828979
CKAP4	2,94960764	0,0001616	0,00833944
TGFB1I1	3,02796148	0,00016201	0,00833944
RASGRF2	4,04650098	0,00016863	0,00865318
PTPRZ1	5,44842222	0,00017074	0,00873197
DCBLD2	1,51656667	0,00017235	0,00873197
CLDN6	5,19395549	0,00017296	0,00873197
DMKN	4,80182048	0,00017302	0,00873197
CRIP2	4,44977028	0,00017338	0,00873197
SMAD9	5,60586532	0,000174	0,00873655
FAT3	4,42227689	0,00017615	0,00881703
PLEKHG3	3,37572215	0,00017785	0,00887518
ADAMTS20	4,2658637	0,00018072	0,00898179
MYH10	3,78619668	0,00018158	0,00898179
NR2F1-AS1	4,80930867	0,00018164	0,00898179
AL390729,1	2,94901731	0,0001855	0,00914503
ELAVL2	5,97079281	0,00018849	0,00926452
PTPN21	2,92948522	0,00018917	0,00927004
OSBPL10	3,2011327	0,00019038	0,00930141
CREB3L1	2,55342403	0,00019177	0,00934103
ADAMTS5	3,03442582	0,00019457	0,00944209
GXYLT2	2,93015468	0,000195	0,00944209
RBMS3	2,41199692	0,00019703	0,00950844
HSPG2	1,8669018	0,00019753	0,00950844
ERBB2	2,15137752	0,00020042	0,00961912
ADAMTS1	2,4909257	0,00020129	0,0096322
MATN2	3,24157025	0,00020226	0,00965073
PDLIM1	2,93725724	0,00020713	0,00977984
SIM2	5,95101241	0,00020768	0,00977984
PALLD	0,99382134	0,00020795	0,00977984
IGF2	3,87197409	0,00020821	0,00977984
ADGRA2	3,43664376	0,00020882	0,00977984
GPR161	2,42819135	0,00020903	0,00977984

Gene Symbol	logFC	PValue	FDR
NCAM2	4,6304112	0,00020917	0,00977984
PCDH1	4,04241074	0,00021053	0,00981532
TNFRSF19	4,92642436	0,00021346	0,00991126
ADGRB2	4,04783718	0,0002138	0,00991126
IGFBP7	2,68573996	0,00021551	0,00993417
DENND2A	5,52409468	0,00021644	0,00994872
FST	3,01891799	0,00021741	0,00996556
TPD52L1	3,13866813	0,00021845	0,00998504
BAHCC1	4,96949892	0,00021968	0,0100128
CGAS	1,90172964	0,00022063	0,0100128
APBB2	1,41931294	0,0002209	0,0100128
TTLL7	3,28632535	0,00022259	0,01006176
TPBG	2,84112338	0,00022427	0,01009605
GALNT5	3,51451425	0,00022503	0,01009605
KIAA1522	2,5120657	0,00022521	0,01009605
PAK3	4,30727633	0,00023003	0,01025868
SPOCK3	5,82638821	0,00023009	0,01025868
CNN3	3,61280659	0,00023886	0,01060849
ISL1	5,97481864	0,00023924	0,01060849
EPDR1	3,33315252	0,00024089	0,01065282
NTNG1	5,23300279	0,00024241	0,01066496
BOC	5,0444872	0,00024263	0,01066496
TC2N	6,38582947	0,00024313	0,01066496
KCNT2	8,4062595	0,0002443	0,01068784
SGMS2	3,53800316	0,00024671	0,01075964
LHFPL6	3,54495555	0,00024726	0,01075964
AIF1L	4,45674288	0,00025192	0,01093318
GPSM1	2,92598499	0,00025328	0,01096285
FGFR3	5,7809185	0,00025925	0,01113872
LINC01614	4,54736409	0,00025939	0,01113872
PTCHD1	4,79051393	0,00026258	0,01121989
MARK1	3,39465913	0,00026265	0,01121989
ADAMTSL1	4,63613008	0,00026422	0,01124752
ARSI	4,3250599	0,00026481	0,01124752
HOXC6	4,53491675	0,00026537	0,01124752
ADAMTS6	3,49328129	0,00026725	0,01128672
CARMN	3,2782835	0,00026768	0,01128672
PPP1R3C	4,37082922	0,00027037	0,01137106

Gene Symbol	logFC	PValue	FDR
CGNL1	3,09405292	0,00027197	0,01140883
ABCC9	5,72262084	0,00027436	0,01146515
P4HA2	2,1185859	0,00027489	0,01146515
KLHL4	4,49128068	0,00027554	0,01146515
VASN	3,84996326	0,00027621	0,01146515
TMEM56	4,82395539	0,00027683	0,01146515
PID1	3,09184226	0,00027927	0,01153684
MEGF6	2,26388512	0,00029063	0,01196343
KDELR3	2,53176409	0,00029156	0,01196343
FERMT1	4,56263207	0,000292	0,01196343
BMP6	2,53406549	0,00029252	0,01196343
SYT11	2,16591059	0,00029575	0,012065
EFNA1	5,63760282	0,00030107	0,01225156
GPR173	5,22758112	0,00030285	0,0122763
C6orf132	4,07597974	0,00030318	0,0122763
ITIH3	3,49089673	0,00030401	0,01227944
VGLL3	3,45878627	0,00030593	0,01232258
ID1	3,09591303	0,00030659	0,01232258
TEX15	8,18840722	0,00030795	0,01234664
FBXL7	2,42598016	0,00031066	0,01240101
MN1	2,97554998	0,00031082	0,01240101
PTGER3	4,10961287	0,00031239	0,01243332
FGF13	3,72814994	0,00031488	0,0124714
PLSCR4	3,7151565	0,00031958	0,01262693
SSC5D	3,66875811	0,00032066	0,01263895
TMEM47	3,28195131	0,0003238	0,0127193
EFHD1	4,43375696	0,00032426	0,0127193
LILRB2	1,93367162	0,00032614	0,01276262
NFATC4	3,95264487	0,00032897	0,0128423
ARHGEF17	1,91664494	0,00033234	0,01293984
B4GALNT4	4,74675298	0,00033305	0,01293984
HOXB3	3,45624075	0,00033855	0,01312205
ASAP3	3,34353829	0,00033956	0,01313002
SYDE1	3,09930829	0,00034088	0,01315001
IFITM3	1,73088608	0,00034246	0,01315695
GATA3	8,31877932	0,00034346	0,01315695
SPOCK2	3,84898274	0,00034391	0,01315695
TNFRSF21	2,05227518	0,00034778	0,01324575

Gene Symbol	logFC	PValue	FDR
RCAN2	4,18736152	0,0003487	0,01324575
PRICKLE2	2,92395953	0,00034948	0,01324575
S100A16	2,69846368	0,00034985	0,01324575
SERPINE1	2,05191754	0,00035199	0,01325271
RASL10B	4,30606773	0,00035219	0,01325271
ZNF558	3,34444808	0,00035252	0,01325271
PDE10A	3,45797765	0,00035329	0,01325271
PCDH17	4,12416718	0,00035457	0,01327043
ROR1	3,37067587	0,00035987	0,01342882
CHN1	2,75469555	0,00036045	0,01342882
RGS5	3,59835837	0,00036664	0,01362831
RAB3D	3,80635214	0,00036767	0,01363554
AFF3	2,43605412	0,00036912	0,01365834
SLCO2A1	4,39565219	0,00037318	0,01377594
PPFIBP1	1,33182417	0,00037399	0,01377594
KIAA1671	2,83244736	0,00037568	0,01380724
DAAM2	2,02604846	0,00037932	0,01389165
GLT8D2	3,94238441	0,00037971	0,01389165
NOVA1	4,11990218	0,00038135	0,01389165
PLTP	1,49471345	0,00038139	0,01389165
SBK1	3,28658615	0,00038266	0,01389395
GPR176	2,3763582	0,00038386	0,01389395
FLNA	1,41456967	0,000384	0,01389395
GATA6-AS1	4,79355463	0,00038848	0,01402485
SYT1	3,52224317	0,00039374	0,01415436
FIBIN	4,35056704	0,00039467	0,01415436
MMP16	3,15462828	0,00039801	0,01420481
GLIS3	2,76994829	0,00039851	0,01420481
SOX11	2,89325517	0,00039869	0,01420481
P3H4	2,03881662	0,00040743	0,01446357
TUB	3,98856693	0,00040943	0,01446357
LOXL4	3,70551375	0,00040966	0,01446357
MEDAG	4,20459331	0,00040977	0,01446357
PLXNB1	3,02987061	0,00041043	0,01446357
CEBPZOS	1,19871306	0,00041127	0,01446357
ARHGEF25	4,85735812	0,0004138	0,01448922
SIX4	3,53007054	0,00041451	0,01448922
TEK	2,83896349	0,00041466	0,01448922

Gene Symbol	logFC	PValue	FDR
DPYSL5	7,9560214	0,00041652	0,01452306
PLA2R1	3,00634535	0,00042659	0,01480172
LRRN4	4,04625187	0,00042723	0,01480172
CASC10	3,73922165	0,00043123	0,01490865
HIF3A	5,07108337	0,00043385	0,01496751
BAIAP2L1	2,97965594	0,00043805	0,01508035
RBP1	3,71471918	0,00044261	0,01520519
IGLON5	3,09639957	0,00044891	0,01538726
FNDC1	4,07785837	0,00044986	0,01538726
KIAA1549	2,52797924	0,00045168	0,01538726
TLN2	1,85583206	0,00045361	0,0154209
CPT1C	3,7799308	0,0004548	0,01542913
LY6E	1,65205417	0,0004574	0,01543663
TENM3	4,43047019	0,00045831	0,01543663
CRIM1	0,97699139	0,00045862	0,01543663
LTBP3	1,8700515	0,0004588	0,01543663
FGL2	0,91987865	0,00046092	0,01544413
TCEAL7	8,00462069	0,00046479	0,015542
FAM110C	4,74373704	0,00046835	0,0156085
KALRN	3,05183579	0,0004687	0,0156085
TRIL	3,86881023	0,00047201	0,01566214
AC098864,1	3,6400364	0,00047223	0,01566214
FHOD3	2,53434232	0,00047408	0,01569169
NNAT	5,47426498	0,00048388	0,01595657
GRID2	5,58721388	0,00048479	0,01595657
FCGR3A	1,97134181	0,00048501	0,01595657
SLC16A2	4,08341117	0,00049331	0,01619674
MPP7	3,41340906	0,00050148	0,01642191
MBOAT2	1,68863237	0,00050218	0,01642191
GLRB	3,75822559	0,0005038	0,01642766
ADAMTS18	7,81842281	0,00050437	0,01642766
MAMDC2	3,2420475	0,00051826	0,01677954
MIAT	2,12399246	0,00052129	0,01684424
TMEM246	3,5358553	0,00052344	0,01688035
KIAA1755	7,78781126	0,00053374	0,01716596
SCRG1	4,39991876	0,00053461	0,01716596
FAM69B	4,41407637	0,00053723	0,01716596
FRMD5	3,64574284	0,00053753	0,01716596

Gene Symbol	logFC	PValue	FDR
CHRM3	3,37881185	0,00053755	0,01716596
WNK2	4,15010061	0,00054315	0,01731066
RAI2	6,03288592	0,00054758	0,01740931
EMILIN1	3,11631908	0,00054838	0,01740931
ANGPT1	4,13825735	0,00054992	0,01742436
LARP6	3,56850258	0,00055139	0,01743727
AHNAK2	2,24109727	0,00055846	0,01756203
PCDH19	2,90167007	0,00055945	0,01756203
SLC6A9	2,9388951	0,00055945	0,01756203
PKIA	3,02448656	0,00056029	0,01756203
APELA	7,78409661	0,00056072	0,01756203
NALCN	4,16833143	0,00056316	0,01757266
ARFGEF3	3,24121519	0,00056321	0,01757266
DSEL	2,13591817	0,0005695	0,01768865
ERVMER34-1	5,48314522	0,0005699	0,01768865
COL5A1	3,0941451	0,00057018	0,01768865
IL11	3,18128731	0,00057291	0,01771636
CHRNA1	2,37737025	0,00057325	0,01771636
SNHG18	3,56858542	0,00057874	0,01785245
DNAJA4	3,39056666	0,00058431	0,01795901
ACTG2	3,16575244	0,0005844	0,01795901
NEFM	5,73014605	0,0005858	0,01796829
SH3D19	1,7393613	0,0005901	0,01800291
PROM1	4,70086113	0,00059063	0,01800291
ASAP2	1,22763116	0,00059135	0,01800291
DOC2B	7,81613996	0,0005918	0,01800291
EGF	7,69416688	0,00059245	0,01800291
GULP1	3,22160803	0,00059586	0,01802221
LRP5	2,74583131	0,00059593	0,01802221
LSAMP	4,10882481	0,00059779	0,01802221
LMOD1	2,60402822	0,00059805	0,01802221
LRRTM4	4,15748591	0,00059942	0,01802221
ADGRA3	1,72807447	0,00059971	0,01802221
KIF7	3,13645753	0,00060196	0,01802899
ZDHHC15	5,24687261	0,0006026	0,01802899
CTGF	2,85850675	0,00060325	0,01802899
PPL	4,20342569	0,0006084	0,01813334
LRRC4B	3,55116246	0,00062148	0,01847241

Gene Symbol	logFC	PValue	FDR
CALB1	5,43258241	0,00062322	0,01849037
FHL2	2,18821836	0,00062513	0,01851329
DACH1	3,82469015	0,00062859	0,018582
HOXA13	3,81996627	0,00063692	0,01879437
SHROOM4	2,95980443	0,00064228	0,01891834
FBLN2	2,09157191	0,00064369	0,01892566
KCNN2	3,67287192	0,00064777	0,01901137
EMCN	3,77541595	0,00065269	0,01904187
SHISA9	4,15201067	0,00065369	0,01904187
EPHA7	3,90531954	0,00065395	0,01904187
PCYT1B	4,59648186	0,00065428	0,01904187
DLG3	3,95619598	0,00065464	0,01904187
HOXA-AS2	5,47958152	0,00065941	0,01914622
CSPG4	2,38912774	0,00066336	0,01922679
SNTB2	1,97279927	0,00066501	0,01924049
COL4A2	2,77049735	0,0006679	0,0192845
FLRT3	4,18036584	0,00066889	0,0192845
HOXA10	3,92319662	0,00067261	0,01935752
TGFB2	1,68927468	0,00067699	0,01942105
BEND7	2,92567318	0,0006772	0,01942105
PLBD1	0,92374479	0,00068312	0,01949726
SEMA3F	3,36881319	0,00068406	0,01949726
SGCE	2,23784286	0,00068416	0,01949726
DKK3	2,91182719	0,00068464	0,01949726
JAK3	1,12165845	0,00068978	0,01960938
THBS2	3,9561092	0,00069558	0,01974007
LGR5	3,52413998	0,00070151	0,01987359
STC2	1,73995742	0,0007095	0,02006534
EFNA5	3,35320209	0,00071227	0,02010862
EGFR	2,86950675	0,00071365	0,02011275
ALPK2	2,30590875	0,00071779	0,02019462
PENK	5,4289204	0,00072825	0,02044776
FZD4	2,01828639	0,00072929	0,02044776
ETS1	1,4182536	0,00073422	0,02055058
IGFBP6	3,55415974	0,00073638	0,02055838
PERP	2,28161818	0,00073702	0,02055838
B3GALT1	7,59818304	0,00074551	0,02075975
RWDD2B	1,68339014	0,00074937	0,02080862

Gene Symbol	logFC	PValue	FDR
CSDC2	4,24814309	0,00075502	0,02091746
RFLNA	5,18618711	0,00075844	0,0209636
BDNF	2,73531182	0,00075925	0,0209636
ZNF736	5,17290319	0,00076409	0,02106147
OAS2	1,91327695	0,00076677	0,02109973
RBPMS2	4,31952868	0,00076971	0,02114508
CEMIP2	0,88079018	0,00077153	0,02115932
DIXDC1	1,98589332	0,00077644	0,02122754
LIN7A	4,79279239	0,00077774	0,02122754
CYFIP1	0,79062623	0,00077792	0,02122754
NES	3,00334505	0,0007826	0,02131954
IQCJ-SCHIP1	7,51651321	0,00078851	0,02144473
AFAP1L2	3,38202483	0,00079327	0,02150251
TCF7L1	2,30137393	0,000802	0,0217033
FOXO6	7,53811968	0,00080654	0,02178981
NIPAL4	4,28831234	0,00080949	0,02183336
PLPPR3	3,37645994	0,00081979	0,02207472
ST6GALNAC3	4,09995899	0,00082598	0,02220482
SIGLEC1	1,94939243	0,00082782	0,02221783
ZNF248	3,06192378	0,00083259	0,02230896
OLFML2A	4,25926596	0,00083816	0,02242145
ZNF532	1,04177818	0,00084016	0,02243827
HOXA11	4,49147737	0,00084316	0,0224816
NDNF	5,31106462	0,00084973	0,02260666
ATP8B2	1,47141699	0,00085062	0,02260666
RNF207	1,59007915	0,00085334	0,02264208
DPP10	3,28267956	0,0008574	0,0227128
IFI44L	2,61130085	0,00086086	0,02273269
PDZD2	4,44156293	0,00086129	0,02273269
FIRRE	5,25414333	0,00086233	0,02273269
DSTN	0,79812436	0,00086945	0,02284642
PTPRM	1,84834432	0,00087329	0,02291056
GFPT2	2,36267601	0,00087503	0,02291922
PAM	1,17987784	0,00088037	0,02302229
TMEM132A	1,58992082	0,00088364	0,02307066
FCGR2B	1,6492713	0,00088858	0,02316277
CTXN1	3,83345508	0,00089092	0,02318669
ABCG1	1,68714438	0,00089648	0,02329411

Gene Symbol	logFC	PValue	FDR
EPHA3	3,91692141	0,00089926	0,02332941
CCDC144NL-AS1	1,79014916	0,00090466	0,02337845
CLIC4	0,8459027	0,00090523	0,02337845
FGD1	3,10514002	0,00090647	0,02337845
HOTAIR	5,7631551	0,00090689	0,02337845
APLP1	2,37725152	0,00090906	0,02339748
CCNJL	2,83585038	0,00091225	0,02340573
SALL1	3,54677652	0,00091823	0,02352217
AGRN	1,83554326	0,0009212	0,02356136
DOK6	6,03635914	0,00093039	0,02375915
ECE1	2,17124734	0,00093708	0,02389266
PEAR1	3,52920802	0,00093879	0,02389889
SLC2A1	1,5429346	0,00094322	0,02397431
BEND4	7,38999959	0,00094937	0,02404585
KANK2	1,50265692	0,00095046	0,02404585
SRPX	1,93267081	0,00095285	0,02406606
PRUNE2	3,46409094	0,00095421	0,02406606
EVA1A	3,31829428	0,00097383	0,02451529
LIFR	2,99959249	0,00097502	0,02451529
EGLN3	2,67263424	0,0009774	0,02453721
ZNF502	3,25600175	0,00098171	0,02460751
ADAMTS12	2,55382019	0,00098452	0,02464021
HOXA5	5,13617195	0,00098709	0,02465578
EFS	5,82318046	0,00098817	0,02465578
SULF1	3,9710595	0,00099605	0,02481453
AC145098,2	3,1005715	0,00100111	0,02490271
ARHGEF28	3,53396576	0,0010033	0,02491899
DCC	4,46069074	0,00101088	0,0250692
NEGR1	2,88476959	0,00101356	0,02509757
NUDT11	2,94962991	0,0010234	0,02530132
WNT5B	2,36584704	0,00102489	0,02530132
ZNF423	5,53737378	0,00102851	0,02535224
DIRAS3	4,27645919	0,00103839	0,02555735
PROSER2	3,42796065	0,00104136	0,02559162
SYNC	2,23602295	0,00105037	0,02576565
TSPYL5	2,45709154	0,00105159	0,02576565
TUBB6	1,60484217	0,00105643	0,02580717

Gene Symbol	logFC	PValue	FDR
CAVIN2	3,96261906	0,00105683	0,02580717
CYP26B1	4,69312869	0,00105813	0,02580717
RBFOX2	1,04774914	0,00105962	0,02580717
DZIP1	2,5128537	0,00106432	0,02588323
SLC27A6	4,36669896	0,00106665	0,02590118
TRNP1	4,00450745	0,0010843	0,02629058
KCNMA1	2,0672627	0,00109669	0,0265336
IFI44	1,85148291	0,00109757	0,0265336
SAMD9L	1,0586216	0,00110861	0,02673356
LAMB2	1,52489768	0,00110912	0,02673356
ST6GALNAC5	2,60879251	0,00111321	0,02677567
EXPH5	3,18945767	0,00111415	0,02677567
AADAT	2,88149166	0,0011161	0,02678322
TEAD4	4,74189019	0,00112738	0,0270141
FAM169A	4,16069741	0,00113947	0,02726375
SOX4	1,21295998	0,00114977	0,02746997
C14orf132	3,13397609	0,00115603	0,02757908
LINC00607	5,07353448	0,00116089	0,02765145
CYB5R2	2,44391655	0,00116321	0,02765145
PRSS12	3,22868989	0,00116415	0,02765145
MAL2	2,93926644	0,00116992	0,02768503
DOK4	1,95445665	0,00117057	0,02768503
DMRTA1	3,99474577	0,00117065	0,02768503
LRRC49	2,99406855	0,00117984	0,02786187
ARHGAP29	3,57059902	0,00118583	0,02796295
ZNF677	1,74681732	0,00119194	0,0280318
CBX2	2,44460587	0,00119218	0,0280318
TRPC4	3,45612963	0,00119978	0,02816976
FBN2	0,70295554	0,00120811	0,02832455
RAB3C	2,18499359	0,00121263	0,02838987
SRSF12	3,20550353	0,00121541	0,02839641
FAM155A	2,82083535	0,00121767	0,02839641
AP000662,1	5,17284355	0,00121813	0,02839641
SV2A	2,85905687	0,00122574	0,02849236
AKAP5	2,217509	0,00123422	0,02864859
INA	2,21053786	0,00124114	0,02873956
SLC40A1	1,54879621	0,00124166	0,02873956
AK4	3,01545941	0,00125326	0,02893721

Gene Symbol	logFC	PValue	FDR
MAGEL2	5,01080957	0,00125374	0,02893721
SLC1A1	4,48708511	0,00125691	0,0289577
GRIN2A	7,23287398	0,00125818	0,0289577
TCHH	7,18459196	0,00126767	0,02913489
SDC1	1,96861463	0,00127304	0,02921723
PLCB1	2,03969147	0,00127591	0,02924202
RASSF9	7,23353897	0,00128203	0,02934099
RBM20	4,07795786	0,00128891	0,02943411
MGAT3	2,64223377	0,0012897	0,02943411
CAVIN3	2,93357888	0,00129332	0,0294754
GCNT4	3,70609111	0,00130305	0,02962928
GLI3	1,97743789	0,0013037	0,02962928
KCNQ1OT1	1,45394593	0,00132196	0,02999113
LRRC7	2,64596712	0,0013233	0,02999113
GLIS2	3,97209915	0,00133952	0,03029967
ZNF528-AS1	4,42110023	0,00134063	0,03029967
HOXA9	4,71025302	0,00135049	0,0304663
MID2	4,82801742	0,0013536	0,0304663
AL513534,1	4,82801742	0,0013536	0,0304663
PKNOX2	3,28882443	0,00136428	0,03065051
DAG1	1,20611075	0,00136554	0,03065051
ANXA2	0,71500289	0,00136907	0,03065209
VPS37B	0,97570631	0,00136937	0,03065209
CCDC85A	7,15488091	0,00140007	0,03125362
ELOVL2	3,0471122	0,00140273	0,03127023
RNF152	3,10960838	0,00140629	0,03127358
AMIGO2	1,33893343	0,00140749	0,03127358
SNAP25	3,48694064	0,00140863	0,03127358
FERMT2	2,33454398	0,00141298	0,03132743
FPGT	4,10171086	0,00141823	0,03137588
AC103702,2	7,15569224	0,00142454	0,03142223
FRMPD4	7,16300455	0,00142496	0,03142223
NECAB1	7,10694735	0,00142986	0,03148789
RAP1GAP	7,11041424	0,00143702	0,03160288
KCNIP4	7,11186655	0,00144709	0,03173868
RCN1	1,74534282	0,00145324	0,0318308
JPH2	2,93539261	0,00146577	0,03206229
GNG11	2,58107343	0,00147148	0,03210748
SPTBN2	2,14019966	0,00147177	0,03210748
CHST9	3,94274578	0,00147407	0,03211456
SEL1L3	0,80267386	0,0014782	0,03214573
AC092807,3	2,62706244	0,00147944	0,03214573
AC125807,2	3,45661733	0,00148192	0,03215678
PTPRU	2,95049993	0,0014839	0,03215699
DDAH1	3,01785266	0,00149027	0,03223683
DSC2	1,22803693	0,00149153	0,03223683
LEF1	2,6329596	0,00149443	0,03225678
PLEKHA7	2,00892311	0,00150437	0,03234147
PCDH10	1,71494858	0,00150466	0,03234147
PCDH9	3,53645417	0,00150483	0,03234147
TMEM132D	7,09194387	0,00151365	0,03245686
RUNX1T1	1,74976897	0,00151913	0,03245859
OPCML	3,43506608	0,00151969	0,03245859
ADCY2	4,24609389	0,00152869	0,03260803
ABHD17C	3,3875925	0,00154581	0,03280168
DOCK6	3,30109211	0,00155395	0,03293172
C4orf19	4,76486114	0,00156146	0,0330478
ANTXR2	0,78837963	0,00157443	0,03327915
PHGDH	1,49877228	0,00158914	0,03346013
PAMR1	4,23961282	0,00160143	0,03367547
ARHGAP28	3,80058058	0,0016115	0,03377265
ANGPTL2	4,50231225	0,00161226	0,03377265
ITGAV	0,70661947	0,00161579	0,03380307
COL4A6	4,25334988	0,00162078	0,03386013
GUCY1A1	1,6772821	0,00162266	0,03386013
EPSTI1	0,98244105	0,00162504	0,03386635
SYT14	7,02274968	0,00162814	0,03388774
LRRN1	4,54434928	0,00164706	0,03423797
AKT3	1,19217102	0,00166315	0,03444128
IAH1	3,51686164	0,00166318	0,03444128
AC102945,2	7,04487335	0,00167363	0,03461381
SEPT8	1,75298436	0,00168138	0,0347301
CTNND2	2,87306218	0,00168643	0,03475652
TBX2	3,44827084	0,00168692	0,03475652
CTSF	4,18632908	0,00174216	0,03584933
F2RL2	2,77786536	0,0017472	0,03588127

Gene Symbol	logFC	PValue	FDR
LURAP1L	3,71056643	0,00174943	0,03588127
PITPNM2	4,35219353	0,00175031	0,03588127
ABLIM1	1,34164457	0,00175478	0,03589068
MYO5B	2,82751868	0,00175879	0,03589068
GABRQ	6,98326175	0,00175956	0,03589068
JCAD	2,53360343	0,00177244	0,03606328
NUMBL	1,55481107	0,00177511	0,03607256
CCDC8	1,3834619	0,00178196	0,03616661
ITM2A	5,17231395	0,00180156	0,03648523
ITGB1	0,69586906	0,00180213	0,03648523
HEG1	0,69152316	0,00181062	0,03661184
ADAMTS15	2,3230055	0,00181502	0,03665535
SCN2A	3,27288977	0,00181827	0,0366707
GBP4	2,64924865	0,00182228	0,0366707
FKBP14	0,85431639	0,00182252	0,0366707
AL590004,3	3,23078029	0,00184109	0,03699874
ECHDC2	0,90796491	0,00184512	0,03703397
GNA14	4,68037356	0,00186422	0,03732567
STMN3	2,29759503	0,00189402	0,03768425
PRDM8	3,81673237	0,00189413	0,03768425
FZD7	1,26726938	0,0018949	0,03768425
HOXB6	4,44282277	0,00189599	0,03768425
AC027290,2	1,20246125	0,00190588	0,03781405
GAS6	1,0041024	0,00190716	0,03781405
CD163	0,71630952	0,00193115	0,0382434
ARNT2	2,8167157	0,00195508	0,03859685
KAZN	2,83130138	0,00195595	0,03859685
CAT	0,72259655	0,0019561	0,03859685
PIANP	3,81717106	0,00196014	0,03863
FLNB	0,78971518	0,00197467	0,03886942
KIAA1549L	3,08994313	0,00199726	0,03926666
RHOD	2,80867469	0,00200053	0,03928362
HHIP	2,672772	0,00200615	0,03933635
NFIA	2,60953135	0,00200804	0,03933635
BICC1	1,39667116	0,00201085	0,03934428
PLIN2	0,88884377	0,00201328	0,0393445
KCNK1	4,87631716	0,00203927	0,03980484
TMEM178B	3,0109359	0,0020438	0,03984557

Gene Symbol	logFC	PValue	FDR
ITGA8	4,69877908	0,00204793	0,03985138
UACA	2,98964496	0,00204898	0,03985138
FAM114A1	1,25394092	0,00206749	0,04016351
CSMD2	4,77026446	0,00207082	0,04018038
ZNF568	1,88353438	0,00207545	0,04022225
STOX2	2,84923316	0,00208497	0,0403437
TMEM178A	2,25855919	0,00208666	0,0403437
JPH1	3,24148538	0,00209507	0,04045836
SETBP1	2,39827312	0,00210261	0,04055607
GPR37	2,57605278	0,00210891	0,04062946
MMP24	2,02241972	0,00212361	0,04079429
ZNF844	2,82701689	0,00212418	0,04079429
ZBTB8B	2,8943884	0,00212518	0,04079429
KLF5	1,81686001	0,00212746	0,04079429
GPX7	1,75245765	0,00215675	0,04125899
ADCYAP1	6,8380029	0,00216721	0,04141048
DRP2	2,66261611	0,002193	0,04180217
FAM229B	2,51423615	0,0021954	0,04180217
GPRC5A	3,10080215	0,00220164	0,04187217
KLHL29	2,41921527	0,00220975	0,04197756
LINGO1	3,453021	0,00221806	0,04208635
SH2D4A	3,93900121	0,00223098	0,04223589
SH3BGRL2	2,0541685	0,00223149	0,04223589
PPP1R12B	0,9007716	0,00223377	0,04223589
SOX5	3,07847763	0,00223629	0,04223589
NID2	4,3679036	0,00224605	0,04237116
GRB10	1,51191186	0,00225835	0,04248369
IFFO2	1,65916853	0,00225881	0,04248369
TUSC1	3,16658464	0,00225983	0,04248369
MAGI2	2,37581792	0,00228958	0,0429935
C11orf87	4,83374538	0,00229442	0,04303489
ZBED9	3,06139502	0,00229868	0,04306536
ZNF391	3,07336969	0,00231738	0,04331607
SAMD12	2,74739001	0,00232935	0,04333855
MYH14	3,22574328	0,00232983	0,04333855
TWIST1	3,50336897	0,00233079	0,04333855
CHCHD2	4,29893413	0,00233153	0,04333855
MIR4697HG	4,70073691	0,00235997	0,0438111

Gene Symbol	logFC	PValue	FDR
ISPD	6,77776776	0,00240703	0,04463403
GALNT13	2,51902565	0,00241545	0,04473905
QPCT	1,45003984	0,00242021	0,04473905
FZD8	2,17384144	0,00242092	0,04473905
AL353746,1	4,85908553	0,00242452	0,0447548
FGFR1	3,86231803	0,00243478	0,04481804
FSTL3	1,31413513	0,00243618	0,04481804
PDZD4	3,66048028	0,00244107	0,04483548
ZNF829	1,85940951	0,00244482	0,04483548
ADAMTS3	3,11170923	0,00244537	0,04483548
NOV	4,47890426	0,00248409	0,04544331
HSPA7	1,27805697	0,00248823	0,04546793
PEG3	2,01779573	0,00249673	0,04548583
ANOS2P	2,69494604	0,00249724	0,04548583
ITGA9	1,53680071	0,00249757	0,04548583
RHOJ	3,38554264	0,00252107	0,04583619
TENM1	4,69400887	0,00252242	0,04583619
NEFL	3,50515611	0,00252954	0,04591445
WNT11	6,73592625	0,00253949	0,04595398
CYP27C1	3,37437251	0,00254057	0,04595398
CELSR1	2,47856131	0,00254299	0,04595398
DPP7	0,75823498	0,00254993	0,0460285
GRB7	6,74020571	0,00256031	0,04616483
ADAMTS14	3,68214751	0,00256416	0,04618309
GLIPR2	0,99421524	0,00257509	0,04632883
SOX9	3,56657224	0,00260145	0,04670014
TENM2	3,58043953	0,00262571	0,0470043
COL4A4	2,73708961	0,00262653	0,0470043
FOXC2	2,72340125	0,00262703	0,0470043
CCKBR	6,72358534	0,00263555	0,04705346
CRMP1	3,41232374	0,00263927	0,04706838
BMP2	1,71261984	0,00265164	0,04723743
MYH7	2,88260439	0,00267836	0,04761081
LINC00665	2,06536537	0,0027132	0,04812397
MAB21L2	2,62474648	0,00272684	0,04823998
COL5A3	3,64354121	0,00272837	0,04823998
MRAP2	3,24052493	0,00272861	0,04823998
OLFML2B	1,53329563	0,00274005	0,04833737

Gene Symbol	logFC	PValue	FDR
LAMC3	4,2792012	0,00275637	0,04855593
PRDM6	3,17292256	0,00275839	0,04855593
ST8SIA2	4,53659615	0,00277922	0,04881283
FOXP2	5,81755518	0,00278324	0,04881283
ADGRG6	1,75621853	0,00278442	0,04881283
KDELC2	0,91897688	0,00278495	0,04881283
OAS3	1,20965939	0,00279035	0,04885511
WNK4	3,01114786	0,00280009	0,04896013
TBL1X	1,07994192	0,0028067	0,04896013
ALPK3	1,5920317	0,00280835	0,04896013
B3GAT1	4,00393693	0,00283448	0,04931031
CLUL1	3,26433676	0,00284552	0,04944957
MME	1,32924232	0,00285407	0,04954539
GNAO1	0,81473677	0,00286222	0,0496341
ETNK2	3,75826054	0,00286974	0,04963598
SBSPON	4,45914557	0,00287008	0,04963598
COL4A1	2,8700364	0,00287164	0,04963598
HECW2	1,94339623	0,00287449	0,04963598
DKK1	2,07308927	0,0028891	0,04983553
CERCAM	1,31781276	0,00289887	0,04995114
KITLG	2,17338291	0,00292819	0,05040311

Protocol 2 CS52-C9n6-M microglia
57 Down-regulated DEGs in ALS vs Isogenic:

Gene Symbol	logFC	PValue	FDR
TP53	-2,0395296	9,51E-06	0,00223233
ZMAT3	-1,4601453	1,66E-05	0,00291182
TMIGD3	-1,7155918	2,04E-05	0,00323882
FCGBP	-1,4196541	3,67E-05	0,00424382
CD14	-1,2215031	4,93E-05	0,00490026
EDA2R	-2,4091162	9,08E-05	0,00651593
GYPC	-1,1322863	0,00013404	0,00751823
RPS4Y1	-1,0749932	0,00015398	0,00813153
PURPL	-2,4506855	0,00017173	0,00873197
MELTF	-1,3633055	0,00021502	0,00993417
LGALS12	-1,2368676	0,00025595	0,01104923
INSIG1	-1,1274886	0,00031325	0,01243707
LRRC37A11P	-3,4451048	0,00034428	0,01315695
FAS	-1,8357617	0,00039459	0,01415436
MIR34AHG	-1,4247353	0,00042394	0,01475034
AC079949,2	-2,302171	0,00045152	0,01538726
STAB1	-1,1008273	0,00046006	0,01544413
CH25H	-2,4437038	0,00050594	0,01644612
LIMK1	-0,9570942	0,00051641	0,01675297
WNT5A	-1,0306891	0,00060896	0,01813334
VSIG4	-0,763918	0,00074981	0,02080862
IGF2BP2	-0,900232	0,00079146	0,02148934
LRRC39	-1,138293	0,00086836	0,02284642
DDB2	-1,1852896	0,0009113	0,02340573
MAFF	-0,8984777	0,00122372	0,02848606
USP6NL	-0,7495463	0,00094908	0,02404585
RPS27L	-0,9627611	0,0013747	0,03072918
CD83	-0,8255031	0,00141901	0,03137588
LYPD1	-1,372381	0,0014428	0,03168716
GPRC5D-AS1	-4,4284663	0,00150628	0,03234147
SLC1A3	-0,6718046	0,00151793	0,03245859
ZNF385B	-1,25534	0,00153693	0,03269299
FCER1A	-0,9390745	0,00153829	0,03269299
C5AR1	-0,7402643	0,00153868	0,03269299
RTTN	-0,7468991	0,00157699	0,03329023
CD28	-0,8989028	0,00158097	0,03333117
AC025569,1	-1,6678842	0,0016056	0,03371966
GNA15	-0,7258881	0,00165032	0,03426193
FUT7	-2,8933833	0,00175648	0,03589068
MDM2	-0,8529706	0,00176614	0,03597985
C1orf162	-1,0089264	0,00184934	0,03707315
LAT2	-1,0310737	0,00187947	0,03758473
TNFSF15	-0,8206931	0,00188553	0,03765987
TNFRSF10B	-0,7059582	0,00214065	0,04099894
RAB3IL1	-0,7919098	0,00217421	0,04149557
OSBPL3	-0,6729282	0,00231581	0,04331607
ANKRD19P	-2,8330892	0,00233186	0,04333855
GAS6-AS1	-0,7390427	0,00243015	0,04480806
LRRC58	-0,6532281	0,0024815	0,04544331
C9orf139	-1,9006456	0,00253326	0,04593084
PAX8-AS1	-0,9045469	0,00259776	0,04668538
OGDHL	-1,3986823	0,00263252	0,04705096
ITGAL	-0,6180683	0,00267844	0,04761081
KCNAB2	-0,6113955	0,00270437	0,04801949
HTRA1	-0,9101869	0,00273475	0,04829623
IPCEF1	-0,6341936	0,00280816	0,04896013
SSXP10	-1,4074533	0,00282591	0,04921361

Protocol 3 CS29-C9n1-M microglia
3 Up-regulated DEGs in ALS vs Isogenic:

Gene Symbol	logFC	PValue	FDR
MAP3K13	4,04887668	1,64E-06	0,01933636
IRAK4	8,39368305	2,65E-06	0,01933636
FPGT	9,12546704	3,49E-06	0,01933636

Protocol 3 CS29-C9n1-M microglia
1 Down-regulated DEGs in ALS vs Isogenic:

Gene Symbol	logFC	PValue	FDR
SFTA1P	-4,1509267	1,07E-05	0,04432069
